# The PENGUIN approach to reconstruct protein interactions at enhancer-promoter regions and its application to prostate cancer

**DOI:** 10.1038/s41467-023-43767-1

**Published:** 2023-12-06

**Authors:** Alexandros Armaos, François Serra, Iker Núñez-Carpintero, Ji-Heui Seo, Sylvan C. Baca, Stefano Gustincich, Alfonso Valencia, Matthew L. Freedman, Davide Cirillo, Claudia Giambartolomei, Gian Gaetano Tartaglia

**Affiliations:** 1 Istituto Italiano di Tecnologia, CHT@Erzelli, Via Enrico Melen 83, Building B, 7th floor, 16152 Genova, Italy; 2 Barcelona Supercomputing Center, Plaça Eusebi Güell, 1-3, 08034 Barcelona, Spain; 3 Department of Medical Oncology, The Center for Functional Cancer Epigenetics, Dana Farber Cancer Institute, Boston, MA 02215 USA; 4 ICREA - Institució Catalana de Recerca I Estudis Avançats, Pg. Lluís Companys 23, 08010 Barcelona, Spain; 5 Eli and Edythe L. Broad Institute, 415 Main St., Cambridge, MA 02142 USA; 6 Health Data Science Centre, Human Technopole, Milan, Italy; 7 Istituto Italiano di Tecnologia, CNLS@Sapienza, Viale Regina Elena, 00161 Rome, Italy; 8 Present Address: Josep Carreras Leukaemia Research Institute, Ctra de Can Ruti, Camí de les Escoles, 08916 Badalona, Barcelona Spain

**Keywords:** Genome informatics, Cancer genetics

## Abstract

We introduce Promoter-Enhancer-Guided Interaction Networks (PENGUIN), a method for studying protein-protein interaction (PPI) networks within enhancer-promoter interactions. PENGUIN integrates H3K27ac-HiChIP data with tissue-specific PPIs to define enhancer-promoter PPI networks (EPINs). We validated PENGUIN using cancer (LNCaP) and benign (LHSAR) prostate cell lines. Our analysis detected EPIN clusters enriched with the architectural protein CTCF, a regulator of enhancer-promoter interactions. CTCF presence was coupled with the prevalence of prostate cancer (PrCa) single nucleotide polymorphisms (SNPs) within the same EPIN clusters, suggesting functional implications in PrCa. Within the EPINs displaying enrichments in both CTCF and PrCa SNPs, we also show enrichment in oncogenes. We substantiated our identified SNPs through CRISPR/Cas9 knockout and RNAi screens experiments. Here we show that PENGUIN provides insights into the intricate interplay between enhancer-promoter interactions and PPI networks, which are crucial for identifying key genes and potential intervention targets. A dedicated server is available at https://penguin.life.bsc.es/.

## Abstract (web summary)

The authors reconstruct high fidelity networks of protein-protein interactions between promoters and enhancers in prostate cancer and demonstrate the potential of such an analytical framework to obtain actionable insights into the disease and potential therapeutic targets.

## Introduction

Enhancer-promoter (E-P) interactions play a crucial role in orchestrating gene expression and ensuring the proper regulation of cellular processes. DNA-binding proteins (DBPs), including transcription factors (TFs), act as key players in this regulatory network by binding to enhancers and bridging additional protein interactions between enhancers and promoters. In this work we define Enhancers-Promoter protein-protein Interaction Network (EPIN) as the local interactome connecting a single promoter with all its interacting enhancers. EPIN interactions are facilitated by various types of intermediate proteins, such as co-activators (e.g., mediators), chromatin structural proteins (e.g., cohesin), and noncoding RNA-binding proteins.

While protein-protein interactions (PPIs) have been extensively studied^1,2^, the integration of chromatin architecture information, specifically through chromosome conformation capture (3C-like) techniques, with PPI analysis is still in its early stages. Joint investigations of chromatin loops and PPIs are crucial for prioritizing functional interactions^3^. However, it is important to note that many of these studies often lack the necessary biological context at various levels.

As of today, the characterization of context specific intermediate PPIs involved in disease pathways and their association with DBPs remains largely unanswered^4^. Previous studies have highlighted the significance of disrupted E-P loops in several human disorders^5–7^. In cancer, enhancers are frequently subject to sequence and structural variations, leading to the dysregulation of TFs and chromatin modifiers, which contribute to oncogenesis^8^. Consequently, targeting these enhancer-driven mechanisms holds great promise for therapeutic interventions in cases such as Prostate Cancer (PrCa)^9^. In this context, advanced techniques such as HiC and its derivative HiChIP^10^, in combination with ChIP-seq, could enable the identification and characterization of specific chromatin interactions between enhancers and promoters. In particular, H3K27ac-HiChIP has emerged as a powerful tool designed to detect and amplify E-P interactions and has been successfully employed to uncover susceptibility genes associated with cancer, including PrCa^11^.

To characterize protein interactions that take place at the E-P contacts, we developed the Promoter-ENhancer-GUided Interaction Networks (PENGUIN) approach. For each promoter annotated in the genome and covered by at least one HiChIP interaction, PENGUIN builds an EPIN by integrating several sources of information: (1) high-resolution chromatin interaction maps enriched for a marker of active E-P activity (H3K27ac-HiChiP); (2) tissue-specific physical nuclear PPIs; (3) high-quality curated binding motifs of protein-DNA interactions; (4) tissue specific gene expression.

To prove the usefulness of our PENGUIN approach, we applied it to uncover EPINs in a PrCA cell line, androgen-sensitive human prostate adenocarcinoma cells (LNCaP), and validate our findings in comparison to a benign prostate epithelial cell line (LHSAR). PrCa is the 2nd most common cancer in men^12^. Its distinct hormone-dependent nature is characterized by high expression and frequent genetic amplification of *AR*. AR is a regulator of homeostasis and proteases transcription, such as *KLK3* encoding PSA (Prostate-Specific Antigen). *AR* gene is also a principal therapeutically targeted oncogene in PrCa^13^. Increased genetic instability resulting in chromosomal rearrangements and high frequency of mutations are deemed indicative of PrCa aggressiveness^14^ for which there is need of ad hoc treatments^15^. Recurrent mutations in *FOXA1*, involved in prostate organogenesis and regulator of *AR* transcription, have been observed in several populations^16,17^. Hundreds of PrCa-associated single nucleotide polymorphisms (SNPs) have been identified by genome-wide association studies (GWAS), including genomic regions within tumor suppressor genes and oncogenes, such as *MYC*^18^. However, the functional relationship between most of these SNPs and PrCa pathophysiology is unknown. This missing part of the picture, together with the growing evidence of abnormal transcriptional programs driven by genetic instability, led us to investigate the role of chromatin architecture in PrCa. In particular, we focused on the nuclear proteins potentially involved in transcriptional regulation through the interaction of promoters and non-coding regulatory elements, enhancers.

By clustering together promoters with similar EPIN structures with PENGUIN, we identified 273 promoters whose genes are enriched in PrCa fine-mapped SNPs, known PrCa oncogenes, and ChIP-Seq-validated binding sites of transcriptional repressor CTCF. The proteins that populate such EPINs constitute putative PrCa-related factors, some of which have not been previously described to be associated with PrCa SNPs or oncogenes. Moreover, the EPINs detected by PENGUIN enable the characterization of distinct molecular cascades enriched in PrCa SNPs at E-P contacts. These represent potential molecular targets in PrCa that cannot be identified through conventional analytical procedures, such as E-P contacts and GWAS overlap. To explore our results we made a dedicated server available at https://penguin.life.bsc.es/.

Our methodology, focusing at the specific EPIN resolution level, reveals a relation between 3D genome conformation and disease phenotype. This relation allows PENGUIN to propose new directions in the molecular characterization of chromatin interactions as well as in the definition of potential targets for molecular screening towards disease treatment.

## Results

### The PENGUIN framework

**Fig. 1:**
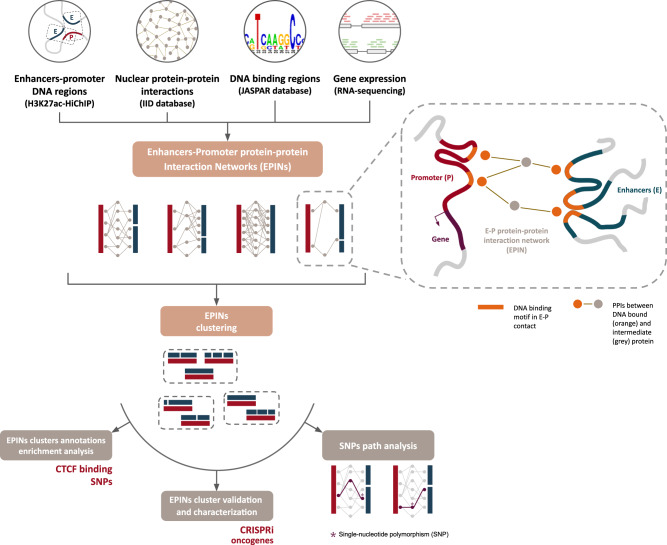
General overview of the PENGUIN workflow and downstream analyses. PENGUIN input consists of HiChIP data (in this work, H3K27ac in LNCaP or LHSAR cell lines), tissue-specific nuclear protein-protein interactions, PPIs (in this work, cancer and normal prostate PPIs from IID database), curated DNA-binding motifs (in this work, motifs from JASPAR database), and gene expression profiles (in this work, RNA-sequencing data in LNCaP or LHSAR cell line). PENGUIN output consists of Enhancer-Promoter protein-protein Interaction Networks (EPINs). Downstream analyses are designed to address specific questions related to prostate cancer (PrCa), namely the identification of clusters of promoters based on EPIN similarity, their enrichment in distinct annotations (CTCF binding from ChIP-seq peaks, PrCa-associated SNPs, and PrCa oncogenes), and finally the formulation of mechanistic hypothesis based on SNPs path analysis. In the inset, we report a schematic representation of an enhancer-promoter protein-protein interaction network (EPIN) reconstructed with PENGUIN for a given E-P contact detected by H3K27ac-HiChIP. Promoter and enhancer DNA binding motifs found in HiChIP regions after enhancer prioritization and the corresponding bound proteins are indicated in orange; their physical interactions with other factors of the EPIN (in gray) are represented as gray lines.

PENGUIN leverages multiple sources of information to build EPINs by grouping enhancers interacting with the same promoter Then, it populates these EPINs with intermediate PPIs and finally clusters the EPINs by structural similarity. The input datasets include (1) high-resolution chromatin interaction maps that capture active promoter-enhancer interactions, highlighting the dynamic nature of gene regulation; (2) tissue-specific physical nuclear protein-protein interactions (PPIs), enabling the exploration of molecular associations within the nucleus; (3) curated binding motifs of protein-DNA interactions, providing insights into the specific interactions between proteins and DNA and (4) gene expression levels, identifying active elements with the interaction networks (Fig. 1). Each EPIN consists of three distinct types of nodes: promoter-bound nodes, encompassing proteins with DNA binding motifs present in the promoter region; enhancer-bound nodes, comprising proteins with DNA binding motifs in the enhancer sequences; and intermediate nodes, representing proteins that interact with either the promoter-bound or enhancer-bound nodes but lack direct DNA binding motifs on the promoter or enhancers. By integrating diverse information, PENGUIN provides a holistic view of the intricate molecular landscape within EPINs. This approach enables the exploration of the interplay between DNA-binding proteins, enhancers, and intermediate proteins, shedding light on the regulatory mechanisms that shape gene expression and ultimately influence cellular functions.

### EPIN composition and properties

In this work, we used 24,547 E-P contacts (30,416 after refinement and prioritization, Methods; Supplementary Fig. S1) identified using H3K27ac-HiChIP data in LNCaP, 810 binding motifs from 639 DNA-binding proteins, and 31,944 prostate-specific, experimentally validated, physical and nuclear PPIs (filtering out proteins from unexpressed genes, Methods; Supplementary Fig. S2) to construct 4,314 EPINs. Each EPIN is centered around one promoter that we found to be contacted by a median of 4 enhancers, with a maximum of 93 enhancers for the promoter of the gene *CRNDE* (Supplementary Data 1). Altogether, the 4,314 EPINs contain a total of 8,215 interactions (edges) among a total of 885 proteins (nodes) that are expressed in LNCaP. A mean of 36% proteins found in these EPINs are encoded by differentially expressed genes in LNCaP versus LHSAR (Methods and Supplementary Data 1).

Overall, 751 out of the 885 proteins represent intermediate nodes, with 127 of them acting both as intermediate and as DNA-bound nodes in different EPINs (Supplementary Data 2). 261 unique DNA-binding proteins have predicted binding sites in at least one of the anchors of enhancers and promoters. A mean of 32.8 (s.d. 11.5) distinct DBPs were identified per promoter anchor with SP1, EGR1, SP2 being the most represented; and a mean of 24.8 (s.d. 7.69) were predicted per enhancer anchor with SP1, IRF1 and TFAP2A being the most represented.

**Fig. 2:**
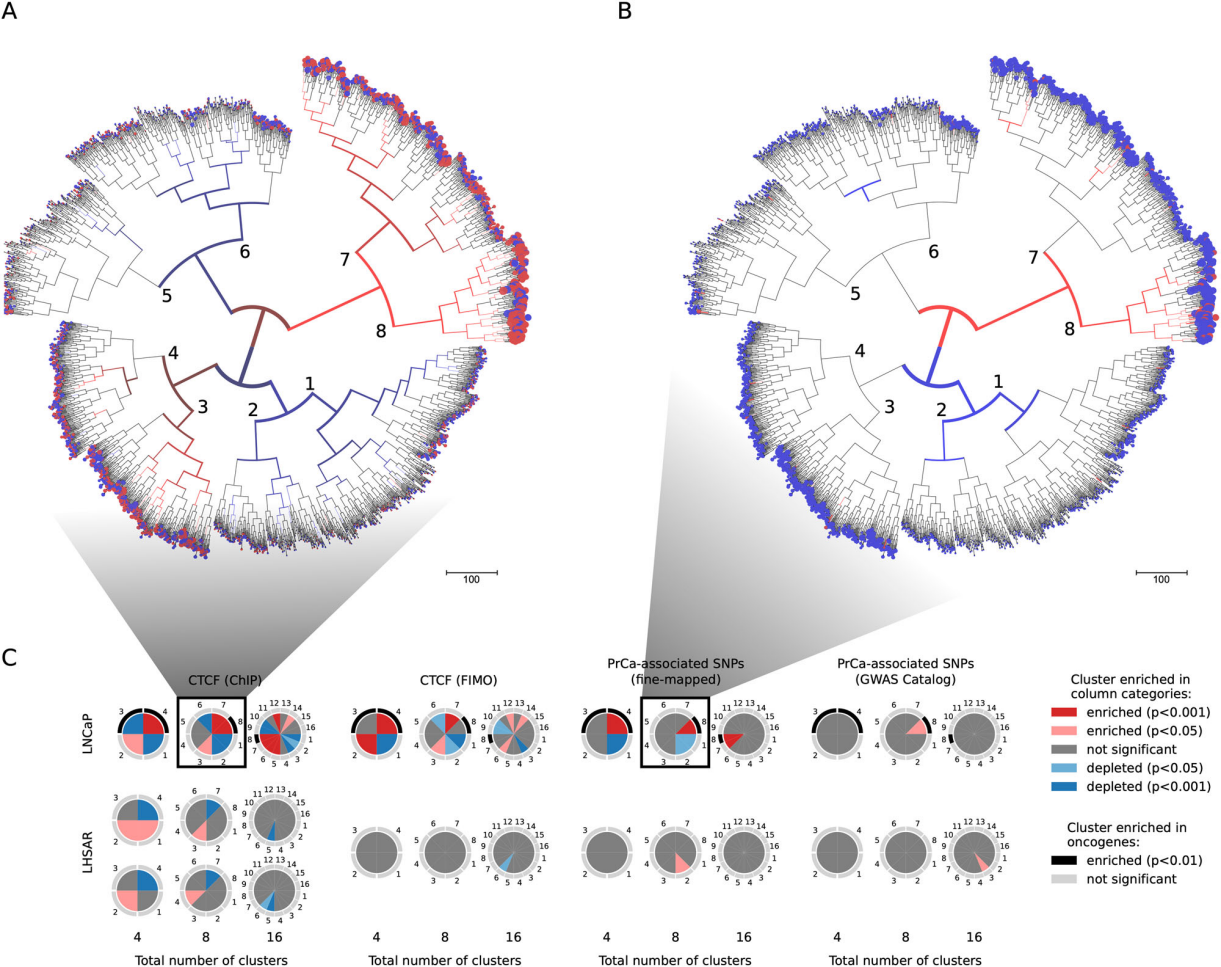
Clustering of the promoters originating the PENGUIN reconstructed EPINs. Clustering is based on edge composition of the EPINs. Leaf radius is proportional to network size. Color code (two-sided Fisher’s exact test): red, enriched; blue, depleted; The figure is generated using ETE3^72^. **A** Enrichment of PrCa SNPs in enhancers. We identified one PrCa SNP enriched cluster (GWAS+; cluster 8), and multiple PrCa SNP depleted (GWAS-; clusters 1, 2) and neutral (GWAS = ; clusters 3, 4, 5, 6, 7) clusters. **B** Enrichment of CTCF ChIP-seq binding sites. We identified multiple CTCF enriched (CTCF+; clusters 3, 7, 8), depleted (CTCF-; clusters 1, 2, 6) and neutral (CTCF=; clusters 4, 5) clusters. **C** Clustering analysis on LNCaP (Top) and LHSAR (bottom) reconstructed EPINs. Pie-charts represent clustering results for a distinct total number of clusters used to partition the hierarchical clustering tree (4, 8, 16). Numbered pie-slices represent the different clusters and their color gradients encode the significance of enrichment (shades of red), depletion (shades of blue) or neutral (gray) of the overlap with distinct annotations (ChIP-Seq CTCF peaks, predicted CTCF binding sites by FIMO, PrCa-associated SNPs from fine-mapping and GWAS). Clusters significantly enriched with previously known oncogenes are annotated with black arcs. All enrichments have been estimated using two-sided Fisher’s exact test. Source data are provided as a Source Data file and Supplementary Data 3B.

A mean of 1.43 (normalized) promoters (0.88 s.d.) are shared among enhancers, with a maximum of 15 promoters for the same enhancer. To identify communalities and differences among the 4,314 EPINs in LNCaP, we performed an unsupervised, hierarchical clustering based on edge composition (Ward’s linkage method, Methods, Fig. 2).

### Characterization of PrCa clusters identified by PENGUIN

As illustrated in Fig. 1, we proceeded to identify, through analysis of annotations, the clusters that show the highest relevance to our disease of interest, PrCa.

Since CTCF is a major actor in the formation and maintenance of transcriptionally productive E-P interactions^19,20^, we searched for enrichment in CTCF binding among PENGUIN clustering of EPINs. For this analysis we used CTCF ChIP-seq peaks, from the same cell line (LNCaP), from the ENCODE^21^ project instead of predictions based on DNA-binding motifs (Methods). We found that the most significantly enriched (*p* value < 0.001) interactions with CTCF peaks concentrate in clusters 7 and 8, which we call *CTCF*+ (Fig. 2A: red branches corresponding to significant enrichment in a two-sided Fisher’s exact test, Methods). This suggests that the presence of CTCF in chromatin interactions results in the formation of characteristic PPI networks between the promoter and its enhancers. Note that this significant enrichment starts when dividing the EPINs in 4 clusters and continues in descendant clusters (dividing in 8 and 16 clusters, see Fig. 2C and Supplementary Data 3).

Beside CTCF enrichment we observed a significant enrichment in PrCa SNPs. This enrichment is most visible in cluster 8 (Fig. 2B) that we call *GWAS*+. We used the previously described 95% credible set of SNPs (henceforth referred to as PrCa SNPs) across 137 PrCa-associated regions fine-mapped from the largest publicly available GWAS summary statistics (*N* = 79,148 cases and 61,106 controls^22^).The result of the PrCa SNP enrichment is displayed in Fig. 2B in blue/red for all possible partitions of our clustering (two-sided Fisher’s exact test, Methods). It should be emphasized that our findings demonstrate consistent results also when employing PrCa-associated SNPs from the GWAS catalog, in which case we also identified GWAS+ cluster as significantly enriched (Methods, Supplementary Data 3).

Interestingly the observed PrCa SNPs enrichment in the GWAS+ cluster is exclusively due to SNPs in enhancers (Table 1). Our results show that E-P interactions containing PrCa SNPs are clustered together (red branches in Fig. 2B) indicating that they have similar characteristics in the way their PPI networks are wired. We found that most pairwise interactions (67.5%, or 5550 out of 8215 edges) are found in all clusters but their final topologies are different.

**Table 1:** Enrichment of PrCa SNPs in cluster 8 (GWAS+) when considering SNPs overlapping enhancers, promoters, either or both

PrCa SNPs overlaps	Odds Ratio (OR)	*p*-value
Only enhancers	11.329	1.80E−12
Only promoters	1.139	0.6
Either enhancers or promoter	8.551	2.68E−11
Both enhancers and promoter	0	1

See also Supplementary Data 3A.

GWAS+ cluster is also enriched in oncogenes. Indeed, among the 273 promoters in GWAS+ cluster, 11 belong to known oncogenes, *FOXA1, ZFHX3, CDKN1B, KDM6A, BRCA2, CDH1, CCND1, NKX3-1, BAG4, MYC, GATA2* (Methods). It is important to emphasize that incorporating intermediate proteins into our reconstructed networks significantly improves the enrichment of functional annotations specifically related to PrCa. By incorporating these intermediate proteins, we were able to increase the number of oncogenes related to PrCa in the GWAS+ cluster from 6 to 11. This also led to a higher level of statistical significance in the enrichment analysis, thereby improving the specificity of our results (see Supplementary Data 4 for details).

We also compared our findings to a simpler approach that relies solely on the overlap between genomic regions of E-P contacts and known oncogene promoters. This information is summarized in Supplementary Data 1, which also includes overlaps between E-P contacts and CTCF peaks, as well as PrCa-related SNPs located within enhancers. Using this simpler overlap approach, only 30 promoters were identified that overlap both PrCa SNPs and CTCF peaks. Among these, just 12 were part of the GWAS+ cluster, and only 3 were promoters of known oncogenes. Notably, only one of these oncogenes, *ZMYM3*, is not already present in the GWAS+ cluster.

We further analyzed properties of GWAS+ cluster. This cluster is enriched in the *Hippo signaling pathway* (KEGG:04390) (Bonferroni-corrected *p*-value = 1.56e−3), *WNT Signaling Pathway* (KEGG:04310) (Bonferroni-corrected *p*-value = 9.57e−3) and *Pathways in cancer* (KEGG:05200) with genes such as *BCL2L1, MYC, FOS* (Bonferroni-corrected *p*-value = 0.047) (Methods, Supplementary Data 5). Interestingly GWAS+ cluster, or any other cluster, did not significantly stand out in terms of overall expression level (Supplementary Fig. S2) or, notably, in terms of fraction of differentially expressed genes (Supplementary Fig. S2).

We identified the protein interactions that are enriched in each cluster and estimated the significance of overrepresentation of each edge in a cluster compared to all others (Methods). GWAS+ cluster exhibits the lowest number of promoters and distinctive network characteristics (Supplementary Data 3A, Supplementary Fig. S3). Nonetheless, per promoter, it displays the largest number of edges (*p*-value < 1e−16) and intermediate nodes (*p*-value < 1e−16), in line with its greater number of enhancers per promoter (*p*-value < 1e−16), see Supplementary Fig. S4.

We also assessed whether PENGUIN clustering was influenced by super-enhancer-like regions sharing target promoters in given clusters. Although the distribution of enhancers per hotspots is similar among our 8 clusters (Supplementary Fig. S4), GWAS+ cluster has fewer single enhancers (enhancer at more than 15 kb from any other enhancer). The average number of promoters targeted by each hotspot for all our 3,752 defined enhancer hotspots was 1.83 promoters targeted per hotspot. When measured considering only the promoters in given EPIN clusters, the values were: 1.29 for cluster 1, 1.28 for cluster 2, 1.25 for cluster 3, 1.24 for cluster 4, 1.22 for cluster 5, 1.21 for cluster 6, 1.34 for cluster 7 and 1.27 for cluster 8. In this case, values were very similar between EPIN clusters.

We note that the EPINs of GWAS+ cluster have the lowest values of node-level centrality measures, namely betweenness and degree (Supplementary Fig. S3). The degree of a node measures the amount of connections it has, while the betweenness centrality measures the amounts of shortest paths that pass through it. Low values of betweenness and degree indicate a lower amount of connections among different nodes of the network. Betweenness and degree are significantly different across clusters (Kruskall–Wallis test *p*-value < 1e−16), but not with respect to the ensemble of all EPINs, which indicates that, despite the high number of shared pairwise interactions (67.5% of edges), the wiring of the cluster-specific EPINs are distinctive.

Finally, to explore the potential connection between our clustering approach and the presence of trans-eQTLs, we used the trans-eQTLs reported from the largest eQTL study available (large-scale meta-analysis in up to 31,684 blood samples from 37 eQTLGen Consortium cohorts in Whole Blood^23^,) and defined a region an ‘eQTL hotspot’ when associated to more than 3 genes (Methods). We observed an enrichment of eQTL hotspots across all clusters (Supplementary Fig. S5, empirical *p*-value < 0.0001), but not specifically for cluster GWAS+ (Supplementary Fig. S5F).

In conclusion, PENGUIN enabled the identification of a cluster of E-P contacts whose EPINs are uniquely enriched in ChIP-seq CTCF peaks, PrCa SNPs and number of oncogenes (Fig. 2 and Table 2). Most importantly, the CTCF and GWAS enrichment, coupled with the highest number of oncogenes, becomes evident when the dataset is partitioned into 8 clusters.

**Table 2:** Enrichment of PrCa SNPs, CTCF ChIP-seq binding sites (“CTCF” in the header), and other PrCa annotations (oncogene promoters and PrCa SNPs from GWAS Catalog) across the eight clusters identified by PENGUIN

Cluster	Number of genes	CTCF	OR	*P*-value	PrCa SNPs	OR	*P*-value	Number of oncogene promoters	OR	*P*-value
			CTCF	CTCF		PrCa SNPs	PrCa SNPs		oncogenes	oncogenes
1	825	−	0.617	1.91E-09	−	0.28	2.46E-02	8	1.17	0.67
2	399	−	0.613	9.65E-06	−	0	2.00E-02	5	1.54	0.38
3	544	+	1.348	1.35E-03	=	0.8	8.27E-01	2	0.39	0.31
4	491	=	1.084	4.09E-01	=	0.51	3.60E-01	4	0.94	1
5	465	=	0.841	9.12E-02	=	0.75	8.14E-01	1	0.23	0.17
6	641	−	0.664	4.24E-06	=	0.38	1.03E-01	1	0.16	0.03
7	676	+	1.655	2.12E-09	=	1.42	3.18E-01	5	0.84	1
8	273	+	3.287	3.64E-20	+	11.33	1.80E-12	11	6.48	1.04E-05

Cluster 8 is enriched in CTCF binding, PrCa SNPs, and oncogenes. Symbols code: + enriched, − depleted, = neutral. OR and *P*-value: two-sided Fisher’s exact test Odds Ratio and *P*-value. See also Supplementary Data 3B.

### Assessment of PENGUIN specificity

To explore the cell and disease-specificity of our results, we applied PENGUIN on LHSAR, a benign prostate epithelial cell line. We performed H3K27Ac HiChIP experimental data and applied the PPI clustering procedure to explore functional relationships within the clusters. We then proceeded to apply PENGUIN to identify clusters of EPINs based on their edges (Methods). As the selection of an exact number of clusters in a given tree could be considered an important variable in our analysis, we examined various cluster numbers (4, 8, 16). We investigated the presence of cluster enrichment in GWAS and CTCF (Supplementary Data 3B). Our analysis did not reveal any cluster enrichment in GWAS and CTCF within the benign prostate control LHSAR. Moreover, we did not observe a significant increase in the number of identified oncogenes in LHSAR (Fig. 2B). These results lead us to conclude that PENGUIN, along with the integration of intermediate PPI networks, significantly enhances the identification of candidate PrCa-related SNPs affecting key elements in chromatin architecture.

Despite the high similarity in PPIs between LHSAR and LNCaP cells (Jaccard index of 0.85), their clustering based on H3K27Ac HiChIP data revealed distinct EPINs (Fig. 2B). This finding highlights the sensitivity of our method in capturing subtle differences within EPINs. To further validate this, we conducted additional statistical analyses on PPIs across different cancer cell types. By examining the overlap between PPI networks, we discovered significant variations that were highly specific to each cell type (Supplementary Fig. S6). This observation not only reinforces the reliability of the differences found in LHSAR and LNCaP cells but also suggests that our results can be expected in other cellular contexts provided the required H3K27ac-HiChIP information, which is currently unavailable in most cases.

To further investigate the significance of intermediate PPI networks, we conducted clustering analysis exclusively based on HiChIP interactions. Specifically, we utilized the list of enhancer IDs, denoted by their genomic coordinates, within each EPIN (Supplementary Fig. S7). Our findings unequivocally demonstrate that the exclusion of intermediate PPI networks substantially diminishes the number of identified oncogenes. This outcome strongly suggests that the information conveyed by the PPI network plays a crucial role in the classification of EPINs and their correlation with phenotypic traits.

### Involvement of E-P protein interactomes in tumor-related functional processes

We next analyzed the functional enrichment of the set of 885 proteins composing the universe of nodes used in the EPINs of LNCaP. 43 out of these 885 proteins are encoded by one of the 122 known PrCa oncogenes (32 intermediates, 7 DBPs among which MGA, ETV4, ETV1, GATA2, ETV3, ERF, NKX3-1, and 4 of both types among which TP53, MYC, FOXA1, AR; see Methods and Supplementary Data 2). In total, 11 out of 885 have been targeted by PrCa-specific drugs (source: DrugBank; protein targets: ESR2, ESRRA, AR, PARP1, NFKB2, NFKB1, NCOA2, NCOA1, AKT1, TOP2A, TOP2B; drugs: Estramustine, Genistein, Flutamide, Nilutamide, Bicalutamide, Enzalutamide, Olaparib, Custirsen, Amonafide); and 190 out of 885 are targets of non-prostate drugs indicating the possibility of re-purposing.

Considering the genes encoding for 477 out of 751 intermediate proteins with annotations for KEGG pathways retrieved using g:Profiler^24^, 41 were annotated in the prostate cancer pathway (KEGG:05215) (adjusted *p*-value = 3.62E−24), which annotates a total of 97 genes (Methods and Supplementary Data 7). We next studied specific protein enrichments in the nodes of the EPINs of each identified cluster (Supplementary Data 8). Although intermediates are ubiquitous and generally shared among all clusters, we could identify 22 significantly specific proteins enriched in the GWAS+ cluster (Methods). Functional enrichment analysis of these 22 proteins revealed significant relationships with tumorigenic processes (Supplementary Data 9). KEGG *Prostate cancer pathway* (KEGG:05215) appears to be highly enriched (adjusted *p*-value = 1.27e−2) together with other pathways related to tumors such as *Colorectal cancer* (KEGG:05210, adjusted *p*-value = 3.20e−5) *Pancreatic cancer* (KEGG:05212, adjusted *p*-value = 9.54e−4) and *Breast cancer* (KEGG:05224, adjusted *p*-value = 7.06e−4). KEGG pathway KEGG:04919 (*Thyroid hormone signaling pathway*) is an additional highly enriched pathway (adjusted *p*-value = 2.57e−4). Thyroid hormones have been previously described as modulators of prostate cancer risk^25–28^. Pathway KEGG:05200 (called *Pathways in cancer*) appears as the fourth most enriched KEGG concept (adjusted *p*-value = 3.63e−4). Other classical tumorigenic pathways, such as *Wnt signaling pathway* (KEGG:04310, adjusted *p*-value = 1.27e−2) and *TGF-beta signaling pathway* (KEGG:04350, adjusted *p*-value = 8.21e−4) appear to be enriched. In this regard, recent studies analyzed the involvement of Wnt signaling in the proliferation of prostate cancer cells^29,30^, as well as the involvement and TGF-beta signaling^31,32^.

Furthermore, we examined the functional enrichment of significantly central proteins across all other clusters. This analysis was conducted to facilitate functional comparisons across different clusters (Methods ‘Functional gene set enrichment analysis’). This analysis revealed no enrichments for clusters 1, 2, 4, 5, and 6 (cluster 5 does not have significantly central proteins). This observation can be attributed to the higher number of central proteins in these clusters (365 in cluster 1, 283 in cluster 2, and 318 in cluster 6) compared to the other clusters (3 in cluster 3, 7 in cluster 7, and 22 in cluster 8). Despite having a similar number of significantly central proteins to cluster 8 (30 proteins), cluster 4 does not show any enrichment.

Moreover, of the clusters presenting enrichments (i.e., clusters 3 and 7), only cluster 7 presents enrichments related to those observed in cluster 8 (for example, KEGG *prostate cancer pathway* is enriched, adjusted *p*-value = 2.041e−2; Supplementary Fig. S8). As commented, cluster 7 presents only 7 significantly central intermediate proteins (CREBBP, CTNNB1, GSK3B, KAT5, MAPK1, PIN1, SMAD2), out of which, 6 overlap with those significantly central in cluster 8 (only PIN1 is absent).

### SNPs path analysis in the E-P protein interactomes

**Fig. 3:**
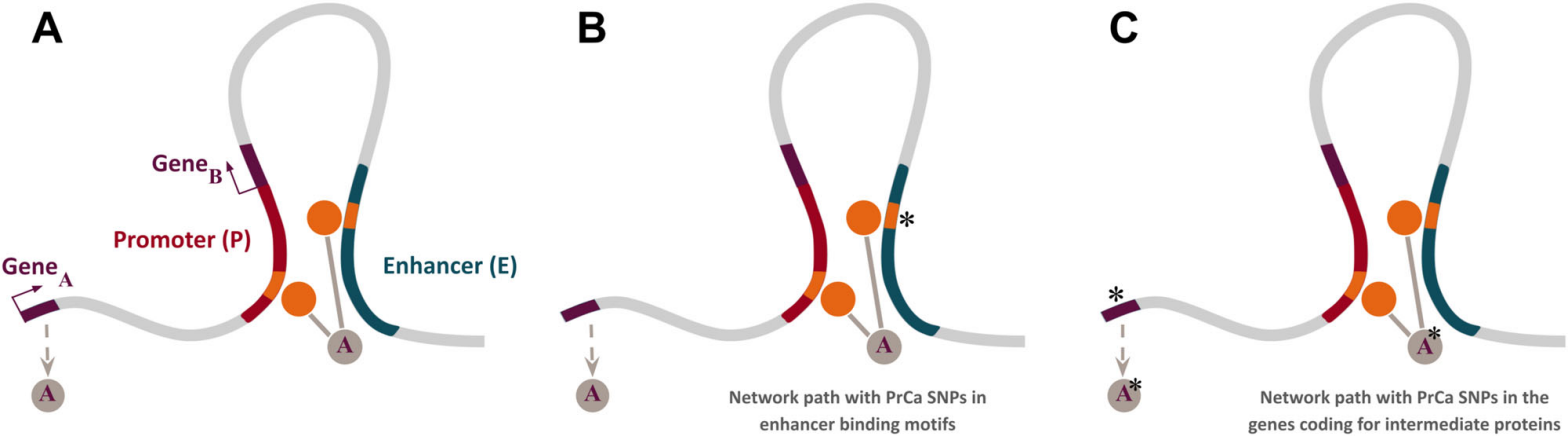
Schematic representation of different types of network paths found in the EPINs reconstructed by PENGUIN. In general, a network path is defined by an intermediate protein (gray circle), encoded by a gene (dark red line; Gene_A_), that interacts with DBPs (orange circles) with binding motifs (orange lines) on the enhancer (green line) and the promoter (red line) of another gene (dark red line; Gene_B_) (**A**). If a PrCa SNP (asterisk) falls in the enhancer binding motif, the interaction between the DBP and the enhancer may be disrupted and possibly its interactions (**B**). If a PrCa SNP (asterisk) falls in the gene that encodes for the intermediate protein, the gene product could be affected and possibly its interactions **(C)**. Colors are consistent with Fig. 1.

Next, we sought to perform an analysis of the SNPs found along the paths within each EPIN (Methods). In this analysis, a path in a network is a sequence of edges joining a sequence of nodes connecting the promoter and the enhancers of an EPIN (Fig. 3A). We distinguish between two possible scenarios based on the location of the SNPs within the paths: (1) PrCa SNPs fall in the DNA binding motifs found in enhancers, indicating a possible dysregulation of TFs binding and activity (Fig. 3B); (2) PrCa SNPs in the genomic regions of the genes that encode for the intermediate nodes of the EPINs, indicating a possible alteration of the PPIs (Fig. 3C). The first analysis aims to identify the location of enhancers that could be targeted by genetic perturbation techniques such as CRISPR/Cas9. The second analysis aims to identify the proteins that are potentially affected by mutations so as to enhance our understanding of prostate cancer biology. Overall, we characterized all PrCa SNPs falling within any path that connects enhancers to a promoter (rs4962419 was found in both scenarios analyzed). In the following, we discuss the two scenarios and report on the *MYC, CASC11 and GATA2* promoters as illustrative examples.

### Network paths with PrCa SNPs in enhancer binding motifs

We sought to detect SNPs located in the DNA binding motifs found in the enhancers of the EPINs. Based on previous evidence^33,34^, our hypothesis is that SNPs in enhancers could disrupt the binding of proteins such as TFs having an impact on their interactome.

In Supplementary Data 10, we list the 36 PrCa SNPs falling within 60 DBP motifs in enhancer regions linking 34 different promoters whose EPINs include 5184 edges. Among these, we identified 17 PrCa SNPs falling within 16 EPINs (1894 edges) belonging to the *GWAS+ cluster* that had at least one PrCa SNP in their enhancers. Several of these EPINs have promoters of differentially expressed genes (such as *DLL1, STOM and SEC11C* in the GEPIA tumor/normal dataset; *ID2, RPS27, SEC11C, CASZ1, CRTC2, C5 and STOM* in the LNCaP/LHSAR dataset; see Methods, Differential Gene Expression).

**Fig. 4:**
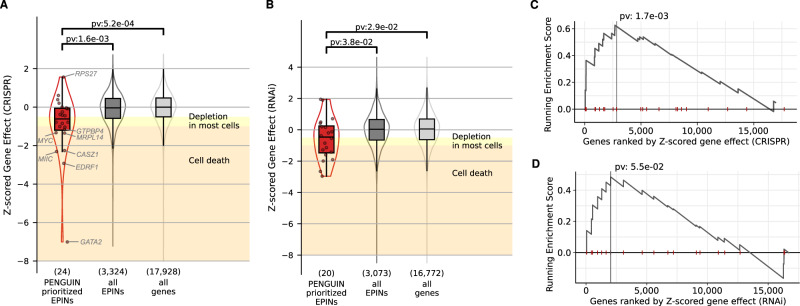
Validation of SNPs prioritized by PENGUIN. CRISPR/Cas9 knockout and RNAi screens provide Z-scores to quantify the relevance of a specific gene network to proliferation of LNCaP cells. **A** CRISPR/Cas9 knockout analysis indicates that intermediate SNPs prioritized by PENGUIN (red) occur in genes essential for LNCaP. Genes with the strongest effect are labeled. **B** RNAi analysis shows milder but significantly consistent results with CRISPR/Cas9 knockout. **A**, **B** The boxes show the interquartile range (IQR), the central line represents the median, the whiskers add 1.5 times the IQR to the 75 percentile (box upper limit) and subtract 1.5 times the IQR from the 25 percentile (box lower limit). Significance calculated with two-sided Mann–Whitney test. **C** Gene Set Enrichment Analysis (GSEA) indicates that SNPs prioritized by PENGUIN occur in the most essential genes identified by CRISPR/Cas9 knockouts. **D** GSEA indicates that SNPs prioritized by PENGUIN occur in the most essential ones based on the RNAi screen. for C and D, the statistical significance of the enrichment of a gene set within the ranked gene list is reported. **C**, **D** Significance is calculated using a two-sided hypergeometric test. Source data are provided as a Source Data file and Supplementary Data 10.

To establish the biological significance of the identified SNPs, we leveraged data from previous pooled genome-wide CRISPR/Cas9 knockout and RNAi screens conducted in prostate cancer LNCaP cells, available in the DepMap database (https://depmap.org/, DepMap ID: ACH-000977). These screens provide essentiality scores, which quantify the relevance of specific gene networks to the proliferation of LNCaP cells. In our analysis, we retrieved essentiality scores for genes in prostate tissue from DepMap and compared three distinct gene sets: (1) the genes (EPIN promoters) prioritized in Supplementary Data 10, (2) all genes (EPIN promoters) included in our analysis, and (3) all genes available in the DepMap database. Remarkably, we observed significant differences in the essentiality scores (Z-scores) among these sets, with lower Z-scores indicating a higher degree of gene essentiality (Fig. 4A). This analysis aligns with the RNAi findings, demonstrating a significant decrease in essential scores for genes containing the SNPs listed in Supplementary Data 10 (Fig. 4B). Furthermore, the GSEA analysis unveiled a noteworthy enrichment (*p*-value = 0.0017) for these EPIN promoters that harbor intermediate nodes with SNPs at their genomic location (as indicated in the supplementary Supplementary Data 10) (Fig. 4C). Among the top essential genes, the CRISPR/Cas9 and RNAi screens prioritize the following ones: GATA2-AS1, CASZ1, MYC, KRT8, GTPBP4-AS1, MFN2, CTBP2, and ID2.

In order to assess the usability of PENGUIN in the absence of HiChIP data, we compared our results with Activity-By-Contact (ABC) scores^35^. We overlapped the fine-mapped GWAS SNPs with the enhancers reported in ABC. 17 out of the 36 SNP-gene links reported in Supplementary Data 10 overlap an enhancer linked to the same promoter in the ABC score. Three SNP-gene links have high support from the ABC model (ABC score ≥0.022), rs55958994-KRT8/KRT18 and rs143499963-DLL1/FAM120B and rs10818488-C5, while rs10090154-MYC/CASC11 have low support (0.0284). Overall these results show a partial overlap when using HiChIP experiments in the PENGUIN approach as opposed to the computational predictions of enhancer-promoter functional contacts to explain the association between SNPs and disease.

Additionally, we used the SNP-Gene-Disease linking strategy cS2G from Gazal and colleagues^36^, to identify 8 SNP-Gene-Disease links with 4 genes (*CTBP2, MYC, ID2, KRT18*) that were also considered in Supplementary Data 10, which could represent links with support from multiple epigenetic information across different cell lines.

Finally, at the level of intermediate proteins, we also found some encoded by genes reported to be differentially expressed. We observed that the mean proportion of intermediates that are differentially expressed is on average 40% (Supplementary Fig. S4). We tested whether promoters belonging to the GWAS+ cluster were significantly enriched for intermediate protein encoding for differentially expressed genes (Methods). Among the 16 EPINs belonging to the GWAS+ cluster that had at least one PrCa SNP in their enhancers, 11 contain expression data to study potential direct effects of the SNPs. In this subset we found 4 EPINs differentially expressed in promoters (3 also differentially expressed in intermediates: *CASZ1, ID2, SEC11C*), and 4 EPINs only differentially expressed in intermediates: *MIIP, MRPL14, MYC, TMEM63B* (Supplementary Data 1). The differential expression of intermediates makes it easier to identify interesting and potentially novel cases. For instance, MYC is not differentially expressed but it has differentially expressed intermediates.

### Network paths with PrCa SNPs in the genes coding for EPIN nodes

In this analysis, we identify EPINs with PrCa SNPs falling within genes that encode either for intermediate or anchor bound nodes (Supplementary Data 11), indicating a potential alteration of PPIs involved in E-P contacts. We found that the GWAS+ cluster has the highest proportion of PrCa SNPs in these nodes compared to all other clusters (mean = 53.2, SE = 18.0, *p*-value ≤ 0.01, Supplementary Data 12). The EPINs of *STK40* and *GATA2* promoters in GWAS+ cluster display the highest fraction of EPIN nodes with PrCa SNPs in their corresponding genes encoding them (Supplementary Data 1).

We used the SNP paths to link 172 PrCa SNPs falling within the gene bodies of 26 genes of which 7 are known oncogenes (*MAP2K1, CHD3, AR, SETDB1, ATM, CDKN1B, USP28*). We identify edges that are most enriched in our GWAS+ cluster which could be pointing to essential links between the gene encoding for the node and containing a PrCa predisposing SNP at a particular EPIN. For example, we identify the link between *MDM4* containing SNP rs35946963 (PrCa *p*-value 1e−24) and TP53^37^ and between *KDM2A* containing SNP rs12790261 (PrCa *p*-value 1e−7) and BCL6^38^ and *ARNT* continuing SNP rs139885151 (PrCa *p*-value 3e−13) and HIF1A^39^.

We integrated information from pQTL associations between the 172 PrCa SNPs and protein levels (Methods). Two intermediate proteins (CREB3L4, MAP2K1) were associated with PrCa SNPs falling within the gene encoding for them (*p*-value of association with proteins were 7.75e−86 for CREB3L4 and 2.40e−5 for MAP2K1). We identified 3 out of 26 promoter EPINs (*TRIM26, MEIS1, POU2F2*) with suggestive evidence (*p*-value < 1e−5) of association between the PrCa SNP with the PENGUIN-linked promoter EPIN, pointing to the cancer promoting mechanistic action of these variants: gene with SNPs in *POU2F2* linked to the EPIN promoter of gene *PHGDH* (SNP with lowest *p*-value rs113631324 = 3.80e−8); gene with SNPs in *TRIM26* and EPIN promoter of gene *RRM2* (SNP with lowest *p*-value rs2517606 = 2.69e−7); gene with SNPs in *MEIS1* and EPIN promoter of gene *STOM* (SNP with lowest *p*-value rs116172829 = 8.19e−6).

We note that, unlike SNPs in enhancers, whose effect can be directly assessed by CRISPR/Cas9 or RNAi assays, the impact of SNPs on intermediate nodes is more complicated to estimate due to their shared involvement in multiple gene networks. In fact, it is worth mentioning that among the 885 proteins identified by PENGUIN, 751 serve as intermediate nodes (section PENGUIN identifies PrCa clusters of protein interactions based on chromatin contacts). This overlapping functionality further complicates the prediction of SNP effects on these intermediate nodes.

### Examples: SNPs path analysis of *MYC*, *CASC11* and *GATA2* promoters

**Fig. 5:**
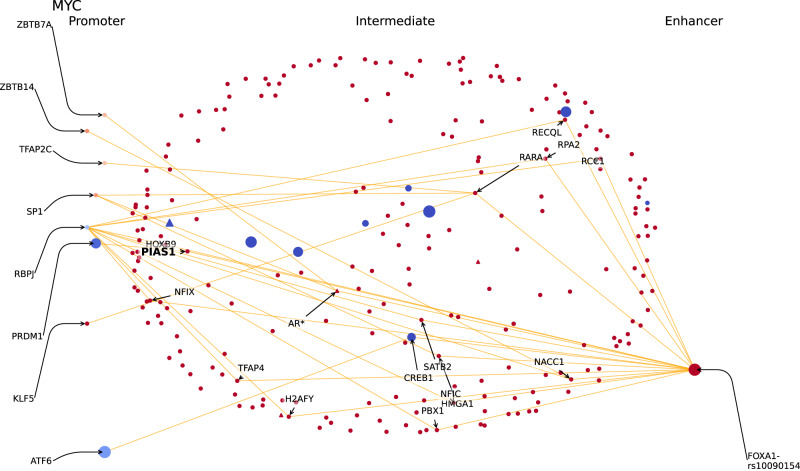
Reconstructed protein interactions between *MYC* promoter and its enhancers. DBPs with binding motifs on the promoter region are aligned on the left, while those with binding motifs on the enhancers are aligned on the right. In the middle, proteins that connect DBPs through a shortest path. Each dot represents a protein. Color, size and shape codes are explained in the Tutorial section of the PENGUIN web service at https://penguin.life.bsc.es/. In this figure, only the edges of network paths with PrCa SNPs in enhancer binding motif are represented (orange lines). Such PrCa SNPs are indicated beside the name of the enhancer-bound DBP (e.g. FOXA1-rs10090154); PrCa SNPs in intermediate proteins are indicated with an asterisk (e.g. AR); the proteins found to be enriched in the GWAS+ cluster are highlighted in bold (e.g. PIAS1); druggable proteins from DrugBank are indicated as triangles.

From HiChIP data, the *MYC* promoter (chr8:128747814-128748813) is in contact with 73 enhancer regions among which one holds the SNP rs10090154 (*p*-value of association with PrCa = 1.4e−188). This SNP is located in the binding motif of the transcription factor FOXA1 on the *MYC* EPIN enhancer. The integration of PrCa SNPs information highlights paths in the EPIN of *MYC* that are particularly compelling in the context of the disease (red line in Fig. 5; Supplementary Fig. S9). The promoter region of *MYC* binds 8 proteins TFAP2C, KLF5, RBPJ, SP1, ZBTB14, ATF6, ZBTB7A, PRDM1 and contains 17 protein interactors (dots in Fig. 4) that might be affected by the possible disruption of its binding motif, namely, HMGA1, RCC1, TFAP4, NFIC, PBX1, HOXB9, NFIX, NACC1, RARA, PIAS1, RPA2, H2AFY, RECQL, SATB2, CREB1, AR. The gene encoding for *FOXA1* is differentially expressed, along with others of its interactors (Supplementary Data 10; Methods). Interestingly, 24 PrCa SNPs fall within the genomic region of *AR* (marked by an asterisk next to the gene name), all with p-values of association with PrCa below 1e-11 (Supplementary Data 11). AR is targeted by several drugs used in the treatment of prostatic neoplasms, such as apalutamide, bicalutamide, diethylstilbestrol, enzalutamide, flutamide, and nilutamide (triangle in the Fig. 4A, source: DrugBank). Notably, mutations in *FOXA1* enhancers were previously shown to alter TF bindings in primary prostate tumors^34^. And, also in line with our observations, *FOXA1* enhancer region has been previously reported to be coupled to *MYC*^40^ and has been shown to have a strong binding of AR^41^.

We report two additional examples, the EPINs for the promoters of *CASC11* (Supplementary Fig. S10A) and *GATA2* (Supplementary Fig. S10B). The EPIN of *CASC11* promoter is also affected by variant rs10090154, the same well known variant associated with risk of developing prostate carcinoma that we introduced with *MYC* EPIN^42,43^ (Supplementary Data 10). Interestingly, *CASC11* is known to enhance prostate cancer aggressiveness and is regulated by C-MYC^44^, while being close to the *MYC* gene on chromosome 8. The promoter binds 6 proteins: TFAP2C, SP3, SP1, PKNOX1, NR2C2 and KLF5. Potentially affected protein interactors of the EPIN include: HMGA1, PIAS1, AR, RARA, and PBX1. GATA2 is an interesting case given its essentiality score from DepMap (Z-score = −7.01). Its EPIN presents up to 11 intermediates affected by PrCa related SNPs, namely TCF4, CTBP2, AR, ARNT, TCF7L2, CDKN2A, NEDD9, ANKRD17, MEIS1, MDM4 and CHD3. The role of GATA2 as mediator of AR signaling in AR-dependent prostate cancer, as well as its role as a potential target for treatment development^45^ has been previously described, as silencing of the gene is known to affect other relevant genes such as *C-MYC* and *AURKA*. Proteins bound to the promoter region include: ZBTB7A, ZBTB33, TCF3, SF1, NR2C2, KLF3, EGR1, E2F1 and CREB1, but most importantly, the EPIN presents AR bound to the enhancer region, which, as we pointed out with *MYC* EPIN, is the target of several PrCa treatments.

## Discussion

Here we introduced the PENGUIN approach that operates on the premise that the EPIN network structure connecting a promoter and its enhancers can serve as a distinctive signature associated with specific functional profiles and diseases. Our assumption is grounded in earlier research that has demonstrated the correlation between 3D loop topology and chromatin state or gene expression^46^. We propose PENGUIN as a molecular approach to study variations in structural characteristics of chromatin loops, establishing a direct link to disease-related phenomena. By integrating the PPI network information, the method offers valuable insights into the underlying mechanisms driving these distinctive features and their relevance to disease progression.

Previous computational approaches have linked enhancers to gene^35^. In this work, PENGUIN uses information on enhancer-promoter interactions using the HiCHIP experiment. There are other ways that this link could be identified. For example, Activity-By-Contact (ABC), a computational prediction method linking an enhancer region to its supposed target gene^35^, could be used instead of HiCHIP observations. We leave this for future explorations. As we note from Results, as well as for HiChIP interactions, PENGUIN is able to complete and extend the information given by solely using enhancer-promoter interactions. In a recent study, Dey et al.^47^ demonstrated the benefits of employing strategies that capture both distal and proximal gene regulation in prioritizing autoimmune-disease related genes. Similarly to our findings, the authors found that incorporating enhancer-gene links (including the ABC score from Fulco et al.^35^), and PPI networks are important to link SNPs-to-gene^47^.

Other previous computation approaches had the goal of linking SNP-Gene-Disease (cS2G from Gazal and colleagues^36^) by combining information across different cell lines. Additionally, previous studies have incorporated PPI networks with GWAS hits to enhance their analysis^48^. Alternative methods have amalgamated information from 3D chromatin interactions and GWAS SNPs to establish connections between intergenic SNPs and gene regulation in cancer contexts^3,49,50^. These approaches have contributed to unraveling the relationship between genetic variations, chromatin organization, and disease. In contrast, our method takes a unique approach by being completely agnostic to the presence of SNPs. It combines information from PPI networks and enhancer-promoter interactions in a cell-specific context derived from H3K27ac-HiChIP data within a unified framework. This integrative approach allows us to leverage both the protein interaction landscape and the regulatory interactions between enhancers and promoters, leading to a comprehensive understanding of the molecular mechanisms underlying disease.

By utilizing PPI networks, we were able to reveal a distinct set of genes associated with PrCa that would have remained undiscovered using other methods. Notably, the intermediate nodes within this PPI network possess intrinsic properties that can be leveraged for the classification and characterization of E-P chromatin loops. Thus, our study demonstrates the capability of PENGUIN to group genes based on their involvement in PrCa, even in the absence of any prior information. This breakthrough opens up an uncharted avenue towards comprehending and identifying unsuspected biological markers in disease. In particular, the genes identified within the cluster exhibiting the highest enrichment in SNPs associated with PrCa (the GWAS+ cluster) can be considered promising candidate oncogenes or potential partners of oncogenes. It is conceivable that these genes may share “onco-enhancers,” which are enhancers contributing to tumorigenic activity. For instance, PENGUIN can be used to identify *trans-*acting factors (e.g., interaction cascades of TFs and chromatin regulators) that could be targeted by drugs, or *cis-*acting factors (e.g., DBPs with binding motifs in regulatory elements) whose DNA binding affinity could be modified through knock-outs via CRISPR for therapeutic intervention. Moreover, unlike traditional TF enrichment analysis which detects general enrichments of particular proteins, PENGUIN can help identify the specific protein cascade potentially disrupted at enhancer loci for the disease under study. Overall, our findings highlight the potential of PENGUIN in unveiling previously unknown gene networks and provide valuable insights into the identification and characterization of biomarkers in various diseases, including PrCa.

To validate our findings, we have used cell-line specific datasets, androgen-sensitive human prostate adenocarcinoma cells (LNCaP) or a normal prostate epithelial cell-line (LHSAR). Each of the sources of information could be directly or indirectly related to the specific cell-lines used in this study: (1) H3K27ac-HiChiP in LNCaP and in LHSAR, (2) prostate-specific PPIs and (3) DNA binding motifs extracted from publicly available datasets but filtered by our cell-type specific interacting 1 kb promoter-enhancer regions and (4) gene expression on cell-line for filtering PPI networks. The comparison of the results in cancer cell-line (LNCaP) to the results in a benign cell line (LHSAR) support our PrCa cell-specific findings. In LHSAR we found a significant association between the obtained clusters and the presence of CTCF, pointing towards the correct classification of EPINs into biologically relevant categories. Strikingly, this same clustering in the benign LHSAR cell-line did not reveal any association to PrCA, neither at the level of PrCa-SNPs, nor at the level of specific oncogenes. Future analyses could explore the use of clustering E-P loops with PENGUIN using other methods and sources for each of these layers. For example, we have used as input enhancer-promoter loops cell-specific H3K27Ac HiChIP experiments (strict calling of loops and prioritization), to maximize our true positives in the input data. The input for the PENGUIN clustering approach can also be constituted by enhancer-promoter links measured from other experimental methods aside from HiChIP or even using computational methods. We leave this for subsequent analyses.

In this work, we use a targeted approach and use the information on association of SNPs from fine mapping as an annotation to our clusters. Specifically, we identify potential SNP paths from defined PrCa associated regions. SNP paths link genes in a network through a path that either starts from TF binding sites in enhancers or passes through proteins from the intermediate EPIN network that would have SNP in their gene bodies. This approach adds a new dimension in the contextualization of GWAS-associated SNPs using the EPIN looping realm.

It is important to mention our primary objective was to shed light on specific links that could be disrupted by PrCa-predisposing variants, such as CTCF bindings that connect promoters to their enhancers, or intermediate structural proteins that play a role in the E-P network. Further investigation is required to gain a comprehensive understanding of the biology and mechanisms underlying these crucial links. For this purpose, and to facilitate the exploration of SNP pathways associated with prostate cancer, we developed a user-friendly web interface accessible at https://penguin.life.bsc.es/. This platform serves as a tool for convenient investigation into the pathways influenced by SNPs in the context of prostate cancer. It is also intriguing to observe that, while PENGUIN successfully identifies clusters of EPINs significantly associated with PrCA, the gene expression analysis did not reveal any significant trends. At first glance, this observation may appear contradictory to our definitions of EPIN clusters and the core concept of EPIN itself. However, considering the evidence presented by our analysis, we believe that PENGUIN enables the detection of cancer associations with heightened sensitivity compared to traditional differential expression analyses. The ability of PENGUIN to capture intricate associations between EPINs and cancer surpasses the limitations of relying solely on gene expression changes, offering a more comprehensive understanding of the underlying molecular mechanisms involved in cancer development and progression.

Our analysis comes with some caveats to keep in mind. Firstly, we relied on data from the HiChIP technique for capturing enhancer-promoter (E-P) interactions, protein-DNA interactions from FIMO, and tissue-specific protein-protein interactions from the integrated interactions database (IID). The comprehensiveness of these datasets is inherently limited by the scope and constraints of the underlying databases and methodologies employed. Furthermore, our approach focuses on networks involving proteins with known edges, resulting in a consideration of only those proteins. Additionally, for the purpose of visualization, we have condensed the number of reported proteins and have presented only one intermediate protein (expanded one edge away). Moreover, it is worth mentioning that our study focuses on E-P interactions within a stable environment (LNCaP cells), representing a snapshot in time. While this field is still undergoing active research and further exploration, existing literature suggests that E-P interactions can exhibit minimal and quantitatively small changes in these conditions. Thus, while interpreting our findings, it is essential to consider the limitations of the utilized databases and methodologies, the specific protein selection, the condensed visualization approach, and the stable cellular context in which the E-P interactions were examined.

In conclusion, the PENGUIN approach employed in this study to investigate PrCa in LNCaP cells has the potential to be applied to the study of other human diseases, given the availability of similar data. This approach can be extended to explore different scenarios, such as different cell types or combinations of GWAS data, offering a promising avenue for future investigations. For instance, utilizing E-P dataset from another prostate cancer cell line would allow the identification of target genes regulated by enhancers from diverse cell types. These target genes can be prioritized using a genome-wide collection of disease-specific risk SNPs. The networks generated by PENGUIN provide a molecular understanding of the associations involved in cancer-related chromatin dynamics, making them well-suited for training advanced machine learning models like graph neural networks (GNNs). We propose potential intermediates in PrCa that engage in E-P networks within cancer cells and present opportunities for therapeutic intervention. High-throughput functional studies could validate the impact of genetic perturbations on thousands of enhancers simultaneously. As shown in our analysis, leveraging CRISPR-Cas9 technology would enable precise editing of specific genomic regions, facilitating targeted investigations and further elucidating the functional consequences of these genetic perturbations.

## Methods

### Experimental methods

#### HICHIP

Trypsinized 10 million cells were fixed with 1% formaldehyde at room temperature for 10 min and quenched. Sample was lysed in HiChIP lysis buffer and digested with MboI (NEB) for 4 h. After 1 h of biotin incorporation with biotin dATP, the sample was ligated with T4 DNA ligase for 4 h and chipped with H3K27ac antibody (DiAGenode, C1541019) after chromatin. Reverse-crossed IP sample was pulled down with streptavidin C1 beads (Life Technologies) treated with Transposase (Illumina) and was amplified with reasonable cycle numbers based on the qPCR with 5-cycle pre-amplified library. Library was sequenced with 150-base pair end reads on the Illumina platform HiSeq® 2500 (Novogene).

#### ChIP

10 million cells were fixed with 1% formaldehyde at room temperature for 10 min and quenched. Cells were collected in lysis buffer (1% NP-40, 0.5% sodium deoxycholate, 0.1% SDS and protease inhibitor [#11873580001, Roche] in PBS).43 Chromatin was sonicated to 300–800 bp with Covaris E220 sonicator (140PIP, 5% duty cycle, 200 cycle burst). H3K27ac antibody (C15410196, Diagenode, 1:600 ratio) was incubated with 40 μL of Dynabeads protein A/G (Invitrogen) for at least 6 h before immunoprecipitation with the sonicated chromatin overnight. Chromatin was washed with LiCl wash buffer (100 mM Tris [pH 7.5], 500 mM LiCl, 1% NP-40, 1% sodium deoxycholate) six times for 10 min each time. Eluted sample DNA was prepared as the sequencing libraries with the ThruPLEX-FD Prep Kit (Rubicon Genomics). Libraries were sequenced with 150-base pair end reads on the Illumina platform HiSeq® 2500 at Novogene.

#### RNA seq

RNA from 5 million cells was extracted using the miRNeasy Micro Kit (#217084, Qiagen) coupled with on-column DNAse I treatment (#79254, Qiagen). RNA sample concentration and its trace were evaluated using a Bioanalyzer RNA 6000 Nano kit (#5067-1511, Agilent) to submit them to Novogene RNA seq dept which prepped RNA library. The library was for RNA seq sequenced to a target depth of 40 M reads on an Illumina platform HiSeq® 2500 with pair-end 150 bp reads. Read alignment, quality control and data analyses were performed using VIPER 57.

### Conformation capture and E-P interactions

We used Hi-C followed by chromatin immunoprecipitation (HiChIP) targeting H3K27Ac in LNCaP cells (androgen-sensitive prostatic carcinoma cell line) across 5 biological replicates including 1 billion reads as previously described^11^ (Table 3). As a comparison, we also performed H3K27Ac HiChIP on LHSAR (Prostate epithelial cells overexpressing androgen receptor), across three replicates including 309 million reads. HiChIP, an efficient protein-mediated chromatin-conformation assay, was performed following the procedure described^10^. The alignment, processing and loop calling from raw fastq files (paired-end data) was performed as previously described^11^. Briefly, HiC-Pro^51^ was used to map the HiCHiP trimmed reads and extract unique interactions; FitHiChIP^52^ was used to identify significant interactions with a predefined set of peaks from H3K27ac ChIP-seq in LNCaP to refine accurate anchor ranges. We used *q*-value < 0.01 and a 5 kb resolution and considered only interactions between 5 kb and 3 Mb as previously described^11^. In this analysis, we restricted to a stringent global background estimation to reduce as much as possible the number of false-positive interactions. The corresponding FitHiChIP specifications used were “IntType=3” (the peak-to-all) for the foreground, meaning at least one anchor to be in the H3K27 peak, and “UseP2PBackgrnd = 1” (the peak-to-peak (stringent)) for the global background estimation of expected counts and contact probabilities for each genomic distance for learning the background and spline fitting. We identified 49,565 significant interactions (FitHiChIP, FDR < 0.01) for LNCaP, and 12,053 for LHSAR.

**Table 3:** Genomic datasets used in the work

	LNCaP	LHSAR
HiChIP H3K27ac	5 replicates (ref. ^11^)	3 replicates (this study)
RNA-seq	2 replicates (Ref. ^71^)	2 replicates (ref. ^71^)
ChIP-seq H3K27ac	1 replicate (Ref. ^11^)	2 replicates (this study)
ChIP-seq CTCF	2 replicates (Ref. ^22^)	2 replicates^a^ (ref. ^22^)

^a^Not from LHSAR but from human epithelial cells of the prostate.

We categorized interactions by overlapping anchors with transcription start sites (TSS) and enhancers identified by H3K27ac ChIP-seq as previously described^11^. Briefly, we first extended anchors by 5 kb on either side; we defined promoter regions around the TSS (+/−500 bases)^53^ using RefSeq hg19; we defined enhancer regions using regions from H3K27ac ChipSeq in the same cell. Specifically, these were 49,638 and 53,561 enhancer regions, respectively from H3K27ac LNCaP in regular media (union of narrow and broad peaks) and from H3K27ac LHSAR. We note that the enhancer anchors at this stage of the analysis are of length 15 kb, due to 5 kb resolution of the HiChIP data analysis and additional 5 kb padding added to anchors on either side. We labeled the promoters and enhancer regions that overlap either right or left anchors, and considered E-P if only one anchor overlaps a promoter and the other an enhancer region. For LNCaP, out of the 49,565 significant interactions, we considered 18,151 E-P interactions. For LHSAR, out of the 12,052 significant interactions, we considered 5435 E-P interactions. It is important to emphasize that our study relies solely on enhancers defined by our own HiChIP experiments, rather than relying on annotated enhancers or external definitions from ENCODE. We further prioritized E-P interactions to 1 kb regions and discarded from enhancers the 1 kb bins with fewer HiChIP interactions with the promoter (see *E-P HiChIP prioritization* section). We obtain 30,416 and 4,497 E-P interactions of 1 kb each for LNCaP and LHSAR respectively. The 15 kb original E-P interactions dataset contained a mean of 1.6 (1.3 s.d.) promoter anchors per enhancer anchor (after prioritization of enhancer anchor to 1 kb region, mean of 1.4 (0.9 s.d.) promoters per enhancer). There were 11,127 (17,683 prioritized 1 kb regions) enhancer anchors in total; 7341 (12,385 prioritized 1 kb regions) enhancer anchors are contacted by one promoter anchor with a maximum of 21 promoter anchors (15 using prioritized enhancer regions) sharing the same enhancer.

### E-P HiChIP prioritization

In order to reduce experimental artifacts in the context of our EPINs, we developed a specific prioritization method. This prioritization start by normalizing the data assuming, as most used capture-C normalizations (ICE^54^, Vanilla, or KR^55^) that all biases (e.g. GC content, number of restriction sites, mappability, or in the case of HiChIP, immunoprecipitation bias) can be corrected together. For this normalization step, we assume that there is a specific bias per any 1 kb genomic loci (β_x_ for loci x; see Supplementary Fig. S1A, B). This bias causes the difference between a theoretical expected number of interactions (E_XY_ between loci *X* and *Y*) and the observed number of interactions (O_XY_ between loci *X* and *Y*). In this representation we can define a system of 9 equations involving three 1 kb loci in the promoter (exactly from TSS −1 kb to TSS + 2 kb) and fifteen 1 kb loci on the enhancer side. This system of equations is then solved using Sequential Quadratic Programming (SQP)^56^. The procedure is repeated in an overlapping window manner along the 15 kb of the enhancer, always against the target 1 kb of the promoter and its two 1 kb neighboring loci. Before the normalization step, we observed a different interaction pattern for interactions below 10 kb (Supplementary Fig. S1C) due, in part, to the contiguity of restriction-enzyme fragments or chromatin persistence length. As these interactions may also be a source of bias in the construction of a PPI network, we removed them from our study. We applied the normalization to the remaining interactions and observed a better correlation between genomic distance and interaction count (Supplementary Fig. S1D).

In order to compare with standard normalization procedure we applied the ICE normalization^54^ to our dataset (using TADbit^57^ 1 kb resolution; filtering bins with less than 100 di-tags − 75% of the genome lost even using a threshold 10 times below the recommended^55^). Because of the sparsity of the genomic matrix the normalization did not fully converge (ICE was not able to completely balance the average di-tag counts per bin^54^). Next we applied the following normalization to our loops dataset, with few modification in order to rescue as much signal as possible: 1- in the promoter site, as our definition of promoter is exact (TSS to TSS + 1 kb), we corrected using the average of the two bins spanning over this 1 kb region 2- on the enhancer site, as most of the 1 kb loci were excluded by the normalization filter we also averaged the ICE bias over the whole region. Even with these modifications, only half the original data was recovered. However, the correlation between genomic distance and number of interactions was significantly improved with respect to raw data. Overall, the correlation value observed with ICE was similar to the one measured for our normalization (Supplementary Fig. S1E). We believe however that, for this dataset and for our methodology, our normalization procedure represents an improvement as it considers the exact promoter regions (not partially overlapping 1 kb bins) and minimizes the loss of promoter-enhancer data.

The normalized profile of interactions was finally used to prioritize most interacting 1 kb loci on the 15 kb enhancer (Supplementary Fig. S1F). The selected 1 kb regions are referred to as prioritized enhancer regions.

### DNA binding motifs

DNA binding motifs were retrieved from JASPAR (Fornes et al. 2019), an open-access database of curated, non-redundant binding profiles of DBPs (a.k.a. motifs) stored as position frequency matrices (PFMs). To detect the binding motifs, we used FIMO from the MEME-suite software (https://meme-suite.org/meme/), with *p*-value ≤ 1e−4 and *q*-value ≤ 5e−2 cutoffs. JASPAR contains 810 DNA binding motifs of 640 proteins that overlap the E-P contacts identified with HiChIP.

### Gene expression data

We assayed RNA sequencing (RNA-seq) in the cell line LNCaP and LHSAR for two replicates using the VIPER pipeline as previously described^11^, and fragments per kilobase of transcript per million mapped reads (FPKM) values were calculated for 20,114 RefSEQ genes. Genes with expression levels above the threshold of 0.003 in both replicates were considered in the entire analysis (Supplementary Fig. S2). Depending on the dataset, this expression lower-bound may be modified in different use cases, for instance based on specific insights or based on a differential analysis between conditions. In this work, we used FPKM instead of more direct measures as we set our threshold very low and did not want to enrich our dataset with very long, virtually unexpressed, transcripts.

### Protein-protein interaction network

We obtained protein-protein interactions (PPIs) from the Integrated Interactions Database (IID)^58^. To better contextualize the interactome information, we combined the annotations of the PPIs from IID database with the LNCaP gene expression data. As for the IID annotations, we applied the following selection criteria. First, we selected interactions annotated as “experimental” in the “*evidence type*” field and identified by at least two independent biological assays reported in the “*methods*” field. Then, we filtered only for interactions in the *prostate* or in *prostate cancer* cells and between *nuclear* proteins. Finally, we retain proteins whose gene expression levels were FPKM > 0.003 in both replicates (this cut-off removes ~30% of the genes). In total, 14,221 proteins from a pool of 20,111 human protein coding genes meet the gene expression criteria. The combination of the above filtering criteria (gene expression, using only nuclear, prostate cancer or prostate and experimentally by 2 methods) resulted in an unweighted network of 31,944 prostate-specific nuclear PPIs among 4295 proteins^58^.

Similarly, for the comparison with the LHSAR cell line we reconstructed the PPI interaction networks with PPIs from the same database (IID) having the following annotation criteria: “experimental” in the “*evidence type*” field and identified by at least two independent biological assays reported in the “*methods*” field. Then, we filtered only for interactions in the *prostate* cells and between *nuclear* proteins. Finally, we retain PPIs between proteins whose LHSAR gene expression levels were FPKM > 0.003 in both replicates. In total 29,316 PPIs representing 4363 proteins were used for the EPIN reconstruction in the LHSAR cell line. Jaccard Index between the two resulting PPIs between LNCaP and LHSAR is 0.852.

### The PENGUIN pipeline

We set up graph-based approach, called Promoter-ENhancer-GUided Interaction Networks (PENGUIN), to reconstruct individual networks of protein interactions that might occur between one promoter (P) and its contacting enhancers (E), that we call E-P protein-protein Interaction Networks (EPINs). To reconstruct the EPINs, PENGUIN integrates information about chromatin contacts, protein-DNA binding, and protein-protein interactions (PPIs). For the case under study in this work (prostate cancer, PrCa), chromatin contacts information comes from H3K27Ac HiChIP of LNCaP cells (4314 promoters and 5789 enhancer regions; see “Conformation capture and E-P interactions”), protein-DNA binding information^55,56^ comes from the JASPAR database (810 DNA binding motifs of 640 proteins; see Methods, “DNA binding motifs”), and PPIs information comes from the IID database (31,944 prostate-specific nuclear PPIs among 4295 proteins; see Methods, “Protein-protein interaction network”) further filtered using LNCaP RNA-seq data (see Methods, “Gene expression data”).

The reconstruction of EPINs follows these steps: for each E-P contact, (1) DNA binding motifs are detected in the corresponding sequences of promoter and enhancer regions; (2) a subnetwork of PPIs is selected containing all promoter-bound proteins, all enhancer-bound proteins, and all their intermediate interactors, with a maximum of 1 intermediate node between enhancer and promoter bound DNA binding proteins; (3) intermediate interactors are discarded if they only connect promoter-bound proteins or enhancer-bound proteins. Using the provided PrCa information, PENGUIN reconstructed 4314 EPINs consisting of a total of 9141 PPIs among 885 proteins of which 751 are intermediate proteins linking promoter-bound and enhancer-bound proteins.

### Node centrality measures

In several analyses we employed two measures of node centrality, namely betweenness and degree. *Betweenness* is a measure of centrality in a graph based on shortest paths. For every pair of nodes in a connected graph, there exists at least one shortest path between the vertices such that either the number of edges that the path passes through is minimized. The degree of a node in a network is the number of connections it has to other nodes; the degree distribution is the probability distribution of these degrees over the whole network.

### Clustering EPINs

We defined EPIN clusters by taking into account their edge content. Each edge consists of an individual pairwise PPI as defined previously. We collected the full universe of edges using all existent edges between all promoter EPINs (the union graph). Then we computed the distance between EPINs by counting the number edges shared over the total number of edges in our predefined universe of edges. Finally, we performed clustering using this distance matrix from all possible combinations of EPIN pairs. The clustering was performed using Ward’s linkage method. Each leaf in the obtained cluster represents a promoter EPIN. In order to assess the robustness of this result we applied the *SigClust2* MonteCarlo procedure^59^ on our clustering with the following parameters: *n_min* = *150, alpha* = *0.05*. We found that the first eight partitions of our hierarchical clustering were very robust with the following Normalized p-values (2-means cluster index): 0, 0, 8e−69, 6e−272, 7e−77, 1e−70, 2e−106 and 2e−77 for clusters 1 to 8 respectively (according to the labeling in Fig. 2).

### Identifying enriched functional annotations in EPIN clusters

We performed two-sided Fisher’s exact tests on every single branch of the dendrogram representing the obtained hierarchical clustering. We evaluated the enrichment of any feature (CTCF binding sites by ChIP-seq, PrCa SNPs from curated GWAS, PrCa oncogenes) in the leaves under a branch of interest compared to those in the rest of the tree. For the enrichment in CTCF binding, we used CTCF peaks from an external dataset but in the same cell line (see CTCF ChiP-Seq peaks). We considered an EPIN to be CTCF-positive (CTCF+), if a CTCF peak was found in a 10 kb region around its promoter and around 10 kb of at least one of its enhancer regions.

For the GWAS feature, we require the presence/overlap of a PrCa-associated SNP (see Genome-wide association data) in at least one of the enhancers of an EPIN. Two-sided Fisher’s exact tests were used to calculate the odds ratio (OR) and enrichment p-values for presence of PrCa annotations within the identified clusters.

### Druggability information

We extracted information for target druggability from DrugBank^60^. The use of each drug was obtained from the Therapeutic Target Database^61^. We annotated each protein node that is a target of drugs that are assigned as Approved or under Clinical Trials (Phase 1, 2, 3) or Investigable for Prostate Cancer, as PrCa druggable.

### CTCF ChiP-Seq peaks

CTCF ChIP-seq peaks for LNCaP cell line were retrieved from ENCODE^21^ project (https://www.encodeproject.org/) for the same Genome assembly, hg19 (GEO references: *GSM2827202* and *GSM2827203*). Overlaps of the CTCF binding sites with enhancer and promoter anchors allowed a 10 kb gap between them. Since CTCF ChiP-seq peaks for LHSAR cell line were not available in ENCODE, we retrieved from ChIP Atlas (https://chip-atlas.org/) two distinct sets (GEO references: *GSM2825573* and *GSM2825574*) of CTCF peaks (of same Genome assembly hg19) for prostate epithelial cells at a *q*-value of 1e−10 (Table 3). We used these two sets independently and in concatenation when comparing the clustering results between LNCaP and LHSAR. These narrow peaks were mapped on the enhancer regions using the python package *PyRanges* (see “E-P contacts” section). For both cases, LNCaP and LHSAR, the narrow peaks were considered as the CTCF binding sites.

### PrCa SNPs

To explore enrichment of SNPs associated to PrCa across the identified clusters, and to identify the SNP paths, we used the previously reported 95% credible set^11^ from fine-mapping 137 previously-associated PrCa regions using a Bayesian statistical method PAINTOR^62^ employing the largest PrCa genome-wide association studies (GWAS) (*N* = 79,148 cases and 61,106 controls)^63^. This set was composed of 5412 distinct SNPs (rsid). We will refer to these as PrCa SNPs. Note that this set also includes SNPs that do not reach genome-wide-filters of p-value significance. We illustrate the location of the associated PrCa regions and number of PrCa SNPs in Supplementary Fig. S11. We did not find a significant correlation between the number of PrCa SNPs in the regions and the number of PrCa SNPs we prioritized in this work (Pearson r = 0.2, *p*-value = 0.06 and Pearson r = 0.1, *p*-value = 0.3 for Supplementary data 10 and 11, respectively). We mapped the SNP location to prioritized enhancer regions anchor locations with a window of 10 kb. 518 out of 5,412 overlap our prioritized enhancer regions; 18 of them overlap our promoter regions. In total 218 prioritized enhancers and 14 promoters overlap a PrCa SNP.

### Annotations of PENGUIN prioritized signals using ABC and cS2G scores

The Activity-By-Contact (ABC) model is a computational tool designed to associate an enhancer region with its potential target gene^35^. In this study, we compared the enhancer-promoter associations derived from H3K27ac-HiChIP in PENGUIN against the predictions made by the ABC method. Although a comprehensive comparison between the two was not feasible, we evaluated the ABC scores for enhancer-promoter links that were integrated into PENGUIN against those that were excluded. We obtained ABC scores for candidate element-gene pairs in the LNCaP cell line from https://osf.io/uhnb4/ (file LNCAP.AllPredictions.txt). Notably, one enhancer can map to multiple genes. Among 14,682 “TargetGene” entries linked to 151,071 genome regions and 8,640,476 enhancer-promoter links, 9567 matched PENGUIN links, while 190,175 did not. The matched ABC links had a higher mean ABC score (0.0156 vs. 0.0039 for all). To assess support from ABC scores for our final SNP-gene links in Supplementary Data 10, we overlapped fine-mapped GWAS SNPs with ABC-reported enhancers. Seventeen of the 36 SNP-gene links overlapped enhancers linked to the same promoter in ABC. Three SNP-gene links had strong ABC support (ABC score ≥ 0.022): rs55958994-KRT8/KRT18, rs143499963-DLL1/FAM120B, and rs10818488-C5, while rs10090154-MYC/CASC11 had lower support (0.0284). This suggests potential new insights from the PENGUIN approach in linking SNPs to diseases.

We also used cS2G from Gazal et al. to complement our SNP-Gene-Disease linking results^36^. cS2G utilizes epigenetic data from various cell lines to link SNPs to diseases. We obtained the data linking 236 SNPs to prostate cancer (168 genes) from the NHGRI-EBI GWAS catalog (gwas_catalog_cS2G). cS2G identified 8 SNP-Gene-Disease links, including 4 genes (CTBP2, MYC, ID2, KRT18) matching those in our Supplementary Data 10. For these genes, at least one SNP in cS2G was in linkage disequilibrium (R2 > 0.6) with a SNP in our PENGUIN analysis. However, the remaining 67 SNP-Gene links in Supplementary Data 10 did not match cS2G’s SNP-Gene links to prostate cancer. Conversely, 48 genes prioritized by cS2G were not in PENGUIN’s Supplementary Data 10. This discrepancy may be due to differences in GWAS data (27/70 SNPs linked in cS2G were not in our fine-mapped dataset). It is important to note that cS2G’s scope differs significantly from PENGUIN. cS2G aims to link SNPs to diseases using diverse epigenetic data from various cell lines, while PENGUIN focuses on identifying cell and disease-specific enhancer-promoter CTCF-mediated pathways

### SNP paths (PrCa SNPs in enhancer binding motifs)

A path in a network is a sequence of edges joining a sequence of nodes. We detected PrCa SNPs located in the DNA binding motifs in the enhancers, and identified the corresponding SNP paths (linked edges and nodes) for each EPIN promoter. For SNP paths analyses and the web-browser, we used all PrCa SNPs in the 95% credible set. There were 36 PrCa SNPs falling in enhancer binding motifs across clusters 3, 4, 5, 6, 7, 8. To report the most interesting cases in the Tables and Results, we used the subset of those passing genome-wide significance of p-value for PrCa association <5e−8. There were 15 PrCa SNPs falling in enhancer binding motifs across clusters 3, 5, 6, 7, 8.

### SNP paths (PrCa SNPs in intermediate proteins)

We detected PrCa SNPs falling within genes that encode for intermediate nodes, and identified the corresponding SNP paths (linked edges and nodes) for each EPIN promoter. For SNP paths analyses and the web-browser, we used all PrCa SNPs in the 95% credible set.

### PrCa enrichment using GWAS Catalog and comparison with other diseases

This analysis had two aims: 1) explore whether we could replicate our finding and identify the GWAS enriched cluster using a different source for the PrCa associated SNPs using SNPs extracted from the GWAS catalog instead of fine-mapped SNPs; 2) to compare the GWAS signal for different diseases. We estimated enrichment of SNPs overlapping the enhancers in each of the identified clusters by exploring the NHGRI GWAS Catalog associations^64^. First, we retrieved GWAS data and filtered the traits according to their “umlsSemanticTypeName” as defined in DisGeNet database^65^ to one of the following: “Mental or Behavioral Dysfunction”, “Neoplastic Process”, “Disease or Syndrome”, “Congenital Abnormality; Disease or Syndrome”, “Disease or Syndrome; Congenital Abnormality”, “Disease or Syndrome; Anatomical Abnormality”. We considered only traits with at least 10 genome-wide-significant SNPs (unadjusted *p*-value < 5e−8). We mapped the SNP location to prioritized enhancer anchor locations with a window of 10 kb. 104 diseases had SNPs overlaps and 17 of them have more than 10 SNP overlapping (Supplementary Data 5). For each cluster, we tested enrichment of disease-associated SNPs using Fisher tests and considered significant *p*-value < 0.01 and OR > 1.

### Trans-eQTL hotspots

We retrieved trans-eQTLs reported in the largest meta-analysis with up to 31,684 blood samples from 37 eQTLGen Consortium cohorts in whole blood in^23^. We grouped enhancers by collapsing when they were separated by less than 20 kb, thereby creating ‘enhancer clusters’. To qualify as a trans-eQTL hotspot, the enhancer clusters had to contain a SNP associated with at least 3 different genes. We quantified the normalized mutual information (NMI) between the hotspot-related enhancer clusters and our 8 EPIN clusters. In order to infer deviation from expected by chance and estimate an empirical p-value, we randomized 10 thousand times the association between each enhancer and its corresponding EPIN cluster and computed the NMI between each randomized EPIN clustering and the observed hotspot-related enhancer clustering. Additionally, we checked if a given cluster was significantly enriched in trans-eQTL hotspots. For this purpose we applied a Fisher test to our pool of enhancers comparing the two contingencies, inside/outside a given cluster, and inside/outside a trans-eQTL hotspot.

### Super-enhancer-like regions

We defined enhancer hotspots as groups of enhancers separated by less than 15 kb, and identified 3752 enhancer hotspots using *bedtools cluster*.

### Oncogenes Gene list

We used a previously identified list of 122 Genes (“PrCa_GeneList_Used.csv”) known to be somatically mutated in PrCa oncogenesis (37 out of 4314 promoters considered). As previously described^11^, the 122 oncogenes are a set of prostate cancer–genes curated from three large-scale PrCa studies that show evidence of somatically acquired mutations, at both localized and advanced prostate cancer, known and recurrently altered in localized prostate cancer and metastatic prostate cancer.

### Enriched edges within each cluster

Two-sided Fisher’s exact tests were used to compute odds ratios and *p*-values of the edges and nodes in the eight different clusters. Specifically, each edge or node was tested for presence/absence in a cluster compared to all others. Therefore, one edge or node can be enriched in one or more than one cluster, it cannot be enriched in all clusters.

### Significantly central intermediate nodes within each cluster

We computed protein importance for each cluster in terms of two network centrality measures: betweenness and degree. For each protein we obtain both betweenness and degree specificity ratios in order to equitably quantify internal protein centrality differences between the clusters. For each of the found clusters we independently estimated the specificity of the observed protein centrality measures (“Betweenness” and “Degree”). For a given protein (P_i_) in a particular cluster (C_j_), we define the specificity (S) as the ratio of the mean centrality (C) of P_i_ within the fraction of *n* networks belonging to C_j_ to the mean centrality of P_i_ within the fraction of *m* networks not belonging to C_j_, NC_j_

$$S(P_i,C_j)=\frac{\frac{1}{n} \mathop{\sum}\limits_{j=1}^{n} C(P_i,C_j)}{\frac{1}{m} \mathop{\sum}\limits_{j=1}^{m} C(P_i,NC_j)}$$

We assessed the significance of protein specificity for each cluster based on random cluster subsamplings. Specifically, we performed 1000 random network samplings to produce random network clusters containing the same amount of networks as the original cluster of interest (i.e. if the original cluster contains 100 networks, the random clusters generated will contain 100 random networks out of the 4314 clustered networks). Within each of those 1000 random clusters, we computed the corresponding protein specificity, with the p-value representing the probability of finding the protein specificity to be higher or equal to the value computed for the original cluster of interest.

We also performed Fisher’s tests to assess enrichment for the presence of the node in the cluster (two-sided Fisher’s exact test *p*-value < 0.01). EP300 was excluded from the enrichment test as the presence of that node was not significantly enriched (two-sided Fisher’s exact test *p*-value < 0.01). 22 proteins (SMAD2, KAT5, NCOR2, MAPK8, SMAD4, CREBBP, CTNNB1, PGR, HDAC3, HDAC2, GSK3B, UBA52, UBE2I, JUND, PIAS1, XRCC5, CDK6, XRCC6, MAPK1, FOS, HIF1A and MAPK3) were found to be significantly specific for both betweenness and degree ratios (*p*-value < 0.01 for both centrality measures and over-represented in this cluster using Fisher’s tests) and used as input for the functional gene set enrichment analysis presented as Supplementary Data 9). We provide the full results of the centrality significance analysis for each cluster in github: [https://github.com/bsc-life/penguin_software/tree/main/Protein_Significance_analysis].

### Functional gene set enrichment analysis

Functional enrichment analysis was performed using the g:GOST module from g:Profiler, a web tool to perform simultaneous gene set enrichment analysis across multiple biomedical databases^24^. We query the web service using the R implementation available from gprofiler2 package. g:GOST performs cumulative hypergeometric tests of an input geneset against preprocessed database-specific gene sets.

The code for this analysis is available as a Jupyter Notebook that can be accessed in github [https://github.com/bsc-life/penguin_software/tree/main/gProfiler_GSEA/Supplementary_Tables_5_7_9_and_Significantly_Central_Protein_Enrichment_Analysis.ipynb].

We set alternative backgrounds for the gene set enrichment analysis, depending on the analysis. For the analysis presented as Supplementary Data 5, where we run the web service to test functional enrichment of the genes associated to the promoter networks from cluster 8, the background is set to the 4314 genes associated with the clustered EPINs. For the analysis presented as Supplementary Data 7, where we test for general functional enrichment of all different proteins forming the EPINs, we run the web service considering only annotated genes for the statistical domain scope. Finally, for the analysis presented as Supplementary Data 9, where we test the functional enrichment of the significantly central (*p*-value < 0.01 for both degree and betweenness centrality) proteins of networks from GWAS+ cluster, the background is formed by the very limited set of 751 unique intermediate proteins forming the EPINs. We additionally provide, within the very same Jupyter Notebook, comparative dot plots presenting the functional enrichment analysis of significantly central proteins of each cluster under Supplementary Data 9 setting.

Reported adjusted *p*-values correspond to Benjamini-Hochberg correction for multiple testing, with adjusted *p*-values ≤ 0.05 considered to be significant. Gene set enrichment analysis results are provided for KEGG pathways, Reactome, Gene Ontology, Wikipathways, TRANSFAC, miRTarBase, Human Protein Atlas, CORUM and Human Phenotype Ontology. For the enrichment analysis of significantly specific proteins of the GWAS+ cluster, we provided as input the 22 previously described proteins. For the enrichment analysis of the GWAS+ cluster, we provided as input all genes associated with the EPIN promoters in cluster GWAS+.

### Differential gene expression

We integrated data from EPIN promoters with differential gene expression (DE) from two sources. DE analysis on prostate cancer tumor versus normal was downloaded from GEPIA: http://gepia2.cancer-pku.cn/#degenes, which use the TCGA and GTEx projects databases to compare gene expression between tumor and normal tissues under Limma, both under and over expressed. We used the default thresholds of log2FC of 1 and qvalue cut-off of 0.01. These data covered 84 out of 885 genes encoding for intermediates in PENGUIN and 413 out of 4314 promoter EPINs. DE analysis of RNA-Seq on LHSAR (an immortalized prostate epithelial line overexpressing androgen receptor) versus LNCaP was performed as previously described. Briefly, RNA-seq data were processed using the VIPER pipeline^66^. Reads were aligned to the hg19 human genome built with STAR. FPKM values were calculated with Cufflinks for 20,114 RefSEQ genes included in the VIPER repository. Differential expression analysis was performed with the DESeq2 R package^67^. 15,650 genes with DE data covered 884 of the 885 genes encoding for intermediates in PENGUIN and 3286 genes out of 4314 promoter EPINs.

We annotated whether the EPIN promoters themselves and the genes encoding the intermediate proteins in our data were DE using either of the two databases. We considered as DE those genes passing |log2 fold change|>1 and adjusted *p*-value ≤ 0.01. For the LNCAP/LHSAR dataset, we could compute a Fisher test of enrichment of differentially expressed genes encoding for intermediate proteins within each EPIN promoter versus within the SNP paths (we could not compute this for the GEPIA since we did not have the full dataset of covered genes). The genes that were not passing these filters were considered non-DE and the genes not covered by the two datasets were excluded from the enrichment analysis described next. For each EPIN we calculated the fraction of DE intermediates within the SNP paths and we estimated the enrichment of those compared to the fraction of DE intermediates in the full EPIN network.

To find the enrichment of DE genes in SNP paths (PrCa SNPs in intermediate proteins) compared to those in the entire EPIN, we computed as enrichment the ratio of Fraction1 / Fraction2, where:

Fraction1 = (number of DE intermediates within SNP paths)/(number of covered intermediates within SNP paths), and

Fraction2 = (number of DE intermediates the EPIN)/(number of covered intermediates in the EPIN).

We report the EPIN genes passing enrichment (“enrichment_DE_deseq_SNP.bs.DBP.path”) > 1.

### pQTL look-up

We downloaded summary statistics with genome-wide association between SNPs and 4907 proteins reported in the deCODE study^68^ and annotated with pQTL association the SNPs we identified falling either in enhancer binding sites or in node genomic locations. The deCODE pQTL summary statistics data contained information on 4907 proteins and 186 (201 PrCa SNPs out of the 213 PrCa SNPs we looked up were in the data and 186 also matched by alleles). 808 out of the 4314 genes promoters (“EPIN_promoters”) and 278 out of the 885 gene intermediates (in total 997 out of 4918 genes promoters and coding for intermediates in our networks) have information on associations with their respective coded proteins covered by the pQTL deCODE data.

### Gene dependency and gene effect metrics

*Gene Effect* and *Gene Dependency* metrics were downloaded from the DepMap portal (https://depmap.org/portal/). We used both the RNAi^69^ and CRISPR^70^ datasets.

### Reporting summary

Further information on research design is available in the Nature Portfolio Reporting Summary linked to this article.

### Supplementary information

Supplementary Information

Peer Review File

Description of Additional Supplementary Files

Supplementary Data 1–12

Reporting Summary

### Source data

Source Data**

## Data Availability

RefSeq hg19 from UCSC Genome Browser is available at the following URL: http://genome.ucsc.edu/cgi-bin/hgTables?hgsid=694977049_xUU5i1QkIJ50dj5miBt9wkAYuxN3&clade=mammal&org=&db=hg19&hgta_group=genes&hgta_track=knownGene&hgta_table=knownGene&hgta_regionType=genome&position=&hgta_outputType=selectedFields&hgta_outFileName=knownGene.gtf. All EPINs and related statistics can be downloaded through the PENGUIN web service at https://penguin.life.bsc.es/. All the raw listed in Table 3, as well as the corresponding processed and metadata for LHSAR and LNCaP related to H3K27ac (HiChIP) and RNAseq have been deposited in GEO (GSE235245). The data can also be downloaded from our github repository (https://github.com/bsc-life/penguin_software/tree/main/data). CTCF ChIP-Seq data used in this work comes from ENCODE^21^ with references GSM2827202, GSM2827203 for LNCaP and GSM2825573, GSM2825574 for the human epithelial cells or prostate that we use to infer CTCF-bindings in LHSAR GSM2825573, GSM2825574. All the datasets used in this manuscript are: HiChIP H3K27ac (5 replicates for LNCaP and 3 replicates for LHSAR), RNA-seq (2 replicates for LNCaP and 2 replicates for LHSAR), ChIP-seq H3K27ac (1 replicate for LNCaP and 2 replicates for LHSAR), ChIP-seq CTCF (2 replicates for LNCaP and 2 replicates for LHSAR). CRISPR/Cas9 knockout and RNAi screens conducted in prostate cancer LNCaP cells were downloaded from the DepMap database (https://depmap.org/, DepMap ID: ACH-000977). Source data are provided with this paper.

## Code availability

Source code of the related to the PENGUIN protocol is available at github: 10.5281/zenodo.10036678. Source code of the related to the PENGUIN web service is available at github: 10.5281/zenodo.10036730. R (v.4.2.0) and Python were extensively used to analyze data and create plots. biomart / ensembl from biomaRt package Ensembl hg19 data for overlaps of SNPs with intermediates.
