# Supplementary material for: The PENGUIN approach to reconstruct protein interactions at enhancer-promoter regions and its application to prostate cancer
Source: Nat Commun. 2023 Dec 6;14:8084. doi: 10.1038/s41467-023-43767-1 (PMC10700545; doi:10.1038/s41467-023-43767-1)
Supplement: Supplementary file 3 — Description of Additional Supplementary Files [file 41467_2023_43767_MOESM3_ESM.pdf]

Description of additional supplementary files

File name: Supplementary Data 1-12

Description: Additional analyses and source data.
