# Supplementary material for: The PENGUIN approach to reconstruct protein interactions at enhancer-promoter regions and its application to prostate cancer
Source: Nat Commun. 2023 Dec 6;14:8084. doi: 10.1038/s41467-023-43767-1 (PMC10700545; doi:10.1038/s41467-023-43767-1)
Supplement: Supplementary file 5 — Reporting Summary [file 41467_2023_43767_MOESM5_ESM.pdf]

nature portfolio

Corresponding author(s): Gian Gaetano Tartaglia
Claudia Giambartolomei
Davide Cirillo

Last updated by author(s): 11/01/2023

Reporting Summary

Nature Portfolio wishes to improve the reproducibility of the work that we publish. This form provides structure for consistency and transparency in reporting. For further information on Nature Portfolio policies, see our Editorial Policies and the Editorial Policy Checklist.

Statistics

For all statistical analyses, confirm that the following items are present in the figure legend, table legend, main text, or Methods section.

n/a	Confirmed	
☐	☒	The exact sample size (*n*) for each experimental group/condition, given as a discrete number and unit of measurement
☐	☒	A statement on whether measurements were taken from distinct samples or whether the same sample was measured repeatedly
☐	☒	The statistical test(s) used AND whether they are one- or two-sided<br>*Only common tests should be described solely by name; describe more complex techniques in the Methods section.*
☐	☒	A description of all covariates tested
☐	☒	A description of any assumptions or corrections, such as tests of normality and adjustment for multiple comparisons
☐	☒	A full description of the statistical parameters including central tendency (e.g. means) or other basic estimates (e.g. regression coefficient) AND variation (e.g. standard deviation) or associated estimates of uncertainty (e.g. confidence intervals)
☐	☒	For null hypothesis testing, the test statistic (e.g. *F*, *t*, *r*) with confidence intervals, effect sizes, degrees of freedom and *P* value noted<br>*Give P values as exact values whenever suitable.*
☒	☐	For Bayesian analysis, information on the choice of priors and Markov chain Monte Carlo settings
☐	☒	For hierarchical and complex designs, identification of the appropriate level for tests and full reporting of outcomes
☒	☐	Estimates of effect sizes (e.g. Cohen's *d*, Pearson's *r*), indicating how they were calculated

Our web collection on statistics for biologists contains articles on many of the points above.

Software and code

Policy information about availability of computer code

Data collection	The data collected in this study was obtained from previous publications (Giambartolomei et al. 2021) and dedicated databases (e.g., binding motifs from JASPAR (Fornes et al. 2019)). HiChIP data was genertated following the procedure described in Mumbach et al. 2016.
Data analysis	HiC-Pro (Servant et al. 2015) was used to map the HiCHIP trimmed reads and extract unique interactions, then refined with FitHiChIP (Bhattacharyya et al. 2019). TADbit (Serra et al. 2017) was used for normalization comparison. FIMO (Grant et al. 2011) was used to identified DNA binding motifs. VIPER pipeline (Cornwell et al. 2018) was used for RNASeq analysis. g:Profiler (Raudvere et al. 2019) was used for biomedical annotations enrichment analysis. All other computational analyses were performed using standard, open-source Python and R libraries.<br><br>Source code of the related to the PENGUIN protocol is available at github: https://github.com/bsc-life/penguin_software https://doi.org/10.5281/zenodo.10036678<br>Source code of the related to the PENGUIN web service is available at github: https://github.com/bsc-life/penguin_analytics https://doi.org/10.5281/zenodo.10036730

For manuscripts utilizing custom algorithms or software that are central to the research but not yet described in published literature, software must be made available to editors and reviewers. We strongly encourage code deposition in a community repository (e.g. GitHub). See the Nature Portfolio guidelines for submitting code & software for further information.

Data

Policy information about availability of data

All manuscripts must include a data availability statement. This statement should provide the following information, where applicable:

- Accession codes, unique identifiers, or web links for publicly available datasets
- A description of any restrictions on data availability
- For clinical datasets or third party data, please ensure that the statement adheres to our policy

RefSeq hg19 from UCSC Genome Browser is available at the following URL:
http://genome.ucsc.edu/cgi-bin/hgTables?hgsid=694977049_xUU5i1QkIJ50dj5miBt9wkAYuxN3&clade=mammal&org=&db=hg19&hgta_group=genes&hgta_track=knownGene&hgta_table=knownGene&hgta_regionType=genome&position=&hgta_outputType=selectedFields&hgta_outFileName=knownGene.gtf

All EPINs and related statistics can be downloaded through the PENGUIN web service at https://penguin.life.bsc.es/

All the raw listed in Table 3, as well as the corresponding processed and metadata for LHSAR and LNCaP related to H3K27ac (HiChIP) and RNAseq have been deposited in GEO (number GSE235245 [https://www.ncbi.nlm.nih.gov/geo/query/acc.cgi?acc=GSE235245]). The data can also be downloaded from our github repository (https://github.com/bsc-life/penguin_software/tree/main/data). CTCF ChIP-Seq data used in this work comes from ENCODE61 with references GSM2827202 [https://www.ncbi.nlm.nih.gov/geo/query/acc.cgi?acc=GSM2827202], GSM2827203 [https://www.ncbi.nlm.nih.gov/geo/query/acc.cgi?acc=GSM2827203] for LNCaP and GSM2825573 [https://www.ncbi.nlm.nih.gov/geo/query/acc.cgi?acc=GSM2825573], GSM2825574 [https://www.ncbi.nlm.nih.gov/geo/query/acc.cgi?acc=GSM2825574] for the human epithelial cells or prostate that we use to infer CTCF-bindings in LHSAR GSM2825573 [https://www.ncbi.nlm.nih.gov/geo/query/acc.cgi?acc=GSM2825573], GSM2825574 [https://www.ncbi.nlm.nih.gov/geo/query/acc.cgi?acc=GSM2825574]. All the datasets used in this manuscript are: HiChIP H3K27ac (5 replicates for LNCaP and 3 replicates for LHSAR), RNA-seq (2 replicates for LNCaP and 2 replicates for LHSAR), ChIP-seq H3K27ac (1 replicate for LNCaP and 2 replicates for LHSAR), ChIP-seq CTCF (2 replicates for LNCaP and 2 replicates for LHSAR). CRISPR/Cas9 knockout and RNAi screens conducted in prostate cancer LNCaP cells were downloaded from the DepMap database (https://depmap.org/, DepMap ID: ACH-000977).

Research involving human participants, their data, or biological material

Policy information about studies with human participants or human data. See also policy information about sex, gender (identity/presentation), and sexual orientation and race, ethnicity and racism.

Reporting on sex and gender	Sex and gender are not considered in this study as it focuses on prostate cancer and uses immortalized cell lines.
Reporting on race, ethnicity, or other socially relevant groupings	Does not apply.
Population characteristics	Does not apply.
Recruitment	Does not apply.
Ethics oversight	Does not apply.

Note that full information on the approval of the study protocol must also be provided in the manuscript.

Field-specific reporting

Please select the one below that is the best fit for your research. If you are not sure, read the appropriate sections before making your selection.

☒ Life sciences ☐ Behavioural & social sciences ☐ Ecological, evolutionary & environmental sciences

For a reference copy of the document with all sections, see nature.com/documents/nr-reporting-summary-flat.pdf

Life sciences study design

All studies must disclose on these points even when the disclosure is negative.

Sample size	H3K27ac HiChIP / ChIP was performed as previously described (https://pubmed.ncbi.nlm.nih.gov/29909987/). 10 million cells were used as this is the standard for these experiments (https://pubmed.ncbi.nlm.nih.gov/34822763/).
Data exclusions	No HiChIP data was excluded, escept non-significant hits as described https://pubmed.ncbi.nlm.nih.gov/34822763/
Replication	Data consists of H3K27Ac HiChIP on LNCAP across 5 biological replicates (https://pubmed.ncbi.nlm.nih.gov/34822763/) and H3K27Ac HiChIP on LHSAR across 3 replicates, thus surpassing the typical minimum accepted number of replicates of two. LHSAR is a control and 3 replicates are OK (i.e. we are not calling the target genes). All replications were successful.

Randomization	Does not apply to this field. Loop calling is what is used instead.
Blinding	Does not apply. Loop calling is what is used instead.

Reporting for specific materials, systems and methods

We require information from authors about some types of materials, experimental systems and methods used in many studies. Here, indicate whether each material, system or method listed is relevant to your study. If you are not sure if a list item applies to your research, read the appropriate section before selecting a response.

Materials & experimental systems

n/a	Involved in the study	
☐	☒	Antibodies
☐	☒	Eukaryotic cell lines
☒	☐	Palaeontology and archaeology
☒	☐	Animals and other organisms
☒	☐	Clinical data
☒	☐	Dual use research of concern
☒	☐	Plants

Methods

n/a	Involved in the study	
☐	☒	ChIP-seq
☒	☐	Flow cytometry
☒	☐	MRI-based neuroimaging

Antibodies

Antibodies used	H3K27ac antibody (DiAGenode, C1541019). We used antibodies targeting H3K27Ac as previously described (https://pubmed.ncbi.nlm.nih.gov/34822763/)
Validation	H3K27ac antibody (DiAGenode, C1541019. We used antibodies targeting H3K27Ac as previously described (https://pubmed.ncbi.nlm.nih.gov/34822763/)

Eukaryotic cell lines

Policy information about cell lines and Sex and Gender in Research

Cell line source(s)	LNCaP and LHSAR human (male) derived cell lines.
Authentication	Cell lines were authenticated via PCR.
Mycoplasma contamination	All cell lines tested negative for Mycoplasma contamination.
Commonly misidentified lines (See ICLAC register)	Does not apply.

Plants

Seed stocks	Does not apply.
Novel plant genotypes	Does not apply.
Authentication	Does not apply.

ChIP-seq

Data deposition

☒ Confirm that both raw and final processed data have been deposited in a public database such as GEO.

☒ Confirm that you have deposited or provided access to graph files (e.g. BED files) for the called peaks.

Data access links *May remain private before publication.*	*For "Initial submission" or "Revised version" documents, provide reviewer access links. For your "Final submission" document, provide a link to the deposited data.*
Files in database submission	*Provide a list of all files available in the database submission.*

Genome browser session
(e.g. UCSC)

http://genome.ucsc.edu/cgi-bin/hgTables?hgsid=694977049_xUU5i1QkIJ50dj5miBt9wkAYuxN3&clade=mammal&org=&db=hg19&hgta_group=genes&hgta_track=knownGene&hgta_table=knownGene&hgta_regionType=genome&position=&hgta_outputType=selectedFields&hgta_outFileName=knownGene.gtf

Methodology

Replicates	H3K27Ac HiChIP on LNCaP was performed across 5 biological replicates as previously described (https://pubmed.ncbi.nlm.nih.gov/34822763/) H3K27Ac HiChIP on LHSAR was performed across 3 biological replicates.
Sequencing depth	H3K27Ac HiChIP on LNCaP includes 1 billion reads as previously described (https://pubmed.ncbi.nlm.nih.gov/34822763/); H3K27Ac HiChIP on LHSAR includes 309 million reads.
Antibodies	We used antibodies targeting H3K27Ac as previously described (https://pubmed.ncbi.nlm.nih.gov/34822763/)
Peak calling parameters	The alignment, processing and loop calling from raw fastq files (paired-end data) was performed as previously described (https://pubmed.ncbi.nlm.nih.gov/34822763/)
Data quality	We identified 49,565 significant interactions (FitHiChIP, FDR<0.01) for LNCaP, and 12,053 for LHSAR.
Software	HiC-Pro (https://pubmed.ncbi.nlm.nih.gov/26619908/) was used to map the HiCHIP trimmed reads and extract unique interactions, then refined with FitHiChIP (https://pubmed.ncbi.nlm.nih.gov/31530818/).
