# Supplementary material for: The PENGUIN approach to reconstruct protein interactions at enhancer-promoter regions and its application to prostate cancer
Source: Nat Commun. 2023 Dec 6;14:8084. doi: 10.1038/s41467-023-43767-1 (PMC10700545; doi:10.1038/s41467-023-43767-1)
Supplement: Supplementary file 2 — Peer Review File [file 41467_2023_43767_MOESM2_ESM.pdf]

nature portfolio

Peer Review File

The PENGUIN approach to reconstruct protein interactions at enhancer-promoter regions and its application to prostate cancer

Open Access This file is licensed under a Creative Commons Attribution 4.0 International License, which permits use, sharing, adaptation, distribution and reproduction in any medium or format, as long as you give appropriate credit to the original author(s) and the source, provide a link to the Creative Commons license, and indicate if changes were made. In the cases where the authors are anonymous, such as is the case for the reports of anonymous peer reviewers, author attribution should be to 'Anonymous Referee' followed by a clear attribution to the source work. The images or other third party material in this file are included in the article's Creative Commons license, unless indicated otherwise in a credit line to the material. If material is not included in the article's Creative Commons license and your intended use is not permitted by statutory regulation or exceeds the permitted use, you will need to obtain permission directly from the copyright holder. To view a copy of this license, visit http://creativecommons.org/licenses/by/4.0/.

REVIEWER COMMENTS

Reviewer #1 (Remarks to the Author):

Armaos and Serra et al. present a really interesting paper that presents a new integrative genomics method called PENGUIN to build protein-protein interaction networks with the additional regulatory layer of chromatin interactions. I think this is a really nice approach and I think the method has a lot of interesting applications to tease out disease mechanisms, especially when looking at variants from non-coding regions.

Interesting method aside, the manuscript is reasonably difficult to read and can be much more concise. The writing itself is fine, however I kept asking "What is the actual focus on the paper?". The way I interpreted the manuscript was that it was actually two separate studies; one being the PENGUIN method and the other being the network of the prostate cancer cell-line. As far as I am aware, the manuscript does not introduce any new data from a previous study (Giambartolomei et al. 2021), so the real innovation is the PENGUIN method. If the paper is rewritten to focus on PENGUIN as a generalised approach, it would make it far more concise and easier to read. I would also suggest complimenting the analysis with an independent dataset (perhaps a non-cancer dataset) which would highlight the broad applicability of the workflow. There is a lot of data out there that could be used in a similar way as the prostate cancer cell-line. Of course, the opposite is also true, focusing specifically results as they relate to prostate cancer, however this would be less interesting to the general audience that Nature Communications aims to target.

Focusing on the the method, I have a few queries regarding the data and design decisions that was used to construct the networks:
- Why is hg19 being used for this analysis? You're missing a lot of very good enhancer information contained in hg38 resources. Given that most of the interaction methods were developed in their previous 2021 paper (Giambartolomei et al. 2021), I expect to see some innovation in the computational methods. While previous versions often have a place in clinical genomics, I see no reason to use hg19, especially given new regulatory element resources such as ENCODE.
- What is a promoter? I am not really a fan of taking non-informed ranges as the promoter, especially when there is some good promoter-specific information out there. Prostate cancer is very well studied so that information should be available. For example, could the authors use CAGE data from Takayama et al. 2015 instead? Would give a really good indication of which promoter is expressed and remove arbitrary length cut-offs such as 500bp.
- Gene expression is mentioned, but where is this coming from? I see that Giambartolomei et al. 2021 used FPKM that is converted to transcripts per kilobase million (TPM), which I would have significant concerns with (use TPM from the start!)
- In your analysis using PENGUIN you don't actually have a baseline because you are describing cancer/cell line-specific data. I realise this method is fine without a base-line comparison, but it would be interesting to see the contrast between a normal prostate tissue dataset? If similar interactions come up, then results would require a different interpretation
- If the expectation is that DNA-protein/TF binding is implicated in disregulation of prostate cancer, then trans-eQTLs (as opposed to cis-eQTLs) would be a really interesting dataset to use for interpretation. trans-eQTLs are often driven by distant TF disregulation (Albert et al. 2018; https://elifesciences.org/articles/35471), rather than local (i.e. cis-) events. Based on your hypothesis, this would be a perfect dataset to include in your PENGUIN method

Regarding the results themselves:
- Correct me if I am wrong, but GWAS catalogue variants are lead-SNPs and therefore indicate a "region" of association, rather than fine-mapped SNPs? I would caution using those SNPs as a basis for looking at enrichment. If a PrCa GWAS is available, then assessing polygenic risk would be a nice way of prioritising important variants? Or perhaps taking all variants within the GWAS SNP region and using a more targeted association test?
- I need more convincing when it comes to the enrichment of CTCF binding sites in the network. As mentioned above, GWAS SNPs used are unlikely to indicate any disease association so enrichment in CTCF binding sites may not have the biological impact that you might expect. Also, CTCF binding sites are everywhere, but *some* are involved in domain formation. Further classifying CTCF sites to ones that are likely to impact regulation or 3D structure would lead to more intepretable results.

- What is the background/universal set of genes/proteins that you are using for gene set enrichment? Gene-set enrichment ALWAYS picks up cancer-related genes. This is because most of the gene-sets are developed from cancer gene expression sets, so there is a massive bias towards them. A control analysis, such as the one suggested above with 'normal' prostate cancer data, would be more convincing.

Overall, I think this is a really nice application of a hypothesis-driven multiomics integration workflow, which is surely needed in the current literature. I hope these suggestions help improve the manuscript before publication

Best regards,

Associate Professor Jimmy Breen
Chief Data Scientist, Black Ochre Data Labs, Telethon Kids Institute
Associate Professor of Indigenous Genomics, Australian National University

Reviewer #2 (Remarks to the Author):

This manuscript established a new approach, called PENGUIN, which was used to identify protein-protein interactions (PPI) in E-P interactions in prostate cancer. Indeed, the authors integrated H3K27ac-HiChIP with a tissue-specific PPI network and gene expression to design this novel approach. This study has presented a good research tool for scientists. Through this tool, key factors that may play a role in transcriptional regulation of prostate cancer will easily be confirmed , and distinct molecular cascades potentially affected by prostate cancer SNPs at E-P contacts can also be identified , opening up new directions to identify molecular targets for disease treatment.

Reviewer #3 (Remarks to the Author):

Armaos et. al. presented a method PENGUIN to identify the PPI interactions supported by HiChIP data (which the authors termed as EPIN) and perform unsupervised clustering to identify a group of promoters with similar EPINs. They observed the enrichment of PrCa-specific GWAS SNPs in the identified P-E networks, particularly in cluster 8. The approach is quite interesting and promises to be useful. However, the utility of the PPI integration and the output causal genes and SNPs need to be thoroughly benchmarked with the existing studies. I, therefore, request a major revision of the manuscript addressing the following comments.

Major Comments

1. The authors used 5 Kb as the lower threshold of interaction distance. Usually, HiChIP or Hi-C interactions below 10 Kb are not reliable. What fraction of HiChIP interactions and what fractions of DNA binding proteins in PPI are within 10 Kb? Does a change of this distance (from 5 Kb to 10 Kb) alter the proposed PPI-based clustering, specifically cluster 8?
2. The authors used a separate normalization scheme (Fig. S5) compared to ICE. The authors need to elaborate on the normalizing equations in Fig. S5 and also compare ICE and the proposed normalization scheme in terms of the Pearson correlation between genomic distance and normalized contact count.
3. It would be interesting to see if the authors employed clustering using HiChIP interactions only and not using the reference PPI and binding proteins. Do the highly connected cluster (and the P-E nodes) from the HiChIP interactions highly overlap with the proposed GWAS+ cluster 8? It may highlight the contribution of the reference PPIs in identifying the functional HiChIP interactions.
4. Table S3, Figs. S1 and S2 show that cluster 8 contains the lowest promoter nodes but the highest number of edges, higher average enhancer connectivity, and higher enhancer-binding proteins. The proposed clustering approach, in effect, identifies highly connected regions. I request the authors to add the following information for cluster 8:

a. Whether the enhancers in cluster 8 are actually parts of one or more super-enhancers.
b. Whether the promoters and enhancers in cluster 8 highly overlap with significant GWAS SNPs (p-value < 5e-8; without any fine mapping).
5. The authors need to show the overlap of causal SNPs and genes reported by PENGUIN with their previous approach to identifying causal genes and SNPs from HiChIP (Giambartolomei et al. 2021). Although they have mentioned putative causal loci and genes (page 10), a comprehensive comparison would be useful.
6. The authors need to benchmark PENGUIN in terms of the causal genes and SNPs reported with the existing approaches for prioritizing genes and SNPs (ABC score, Dey et al. 2022).
7. On pages 9-10, the authors mention that identifying DEGs even in intermediate nodes is useful, while some known oncogenes (like MYC) may not be DEG. Given that such biological significance of many genes may not be known, the authors need to formally characterize the set of genes to be used after DEG analysis.
8. The web server mentioned is not loading.
9. The authors need to describe the column names in individual supplementary excel sheets. The fields are not properly explained.

Minor Comments

1. Page 8, lines 273-280: Figs. 1A-1C should be referred to as Figs. 3A-3C.
2. Table S3, 2nd column: n_networks while the 2nd column in Table 2: number of genes – contradiction.

Reviewer #4 (Remarks to the Author):

The manuscript by Armaos et al. presents an interesting tool called PENGUIN (Promoter Enhancer Guided Interaction Network) which integrates HiChIP-H3K27ac, tissue-specific protein-protein interaction network, gene expression and TF binding motif datasets. However, there are some issues that need to be addressed. They are as follows:

1. Since most of the downstream and comparative analyses were performed on the clusters, it is essential to discuss and address the robustness of the clusters. It needs to be clarified how the number of clusters or k was chosen after hierarchical clustering was performed, i.e., how they came up with 8 clusters in Figure 1/Figure S1A? Typically the number of clusters depends upon where the cut is performed on the dendrogram. So, please elaborate on how this decision was made. Were the models compared to other k?

2. Since it is widely known and reported that hierarchical clustering works poorly with the mixed data types (which have been used for the development of the tool) and missing datatypes (which is quite possible in the next-gen sequencing datasets), the authors should comment on the choice of using hierarchical clustering. Please make proper comparisons with other clustering methods, including k-means, NMF.

3. It is very alarming that the authors have not shared the scripts/code during the review process. I recommend that the authors share the scripts so that anyone and everyone can go over them and understand the approach methodically.

4. Moreover, the webserver "https://penguin.life.bsc.es/" cannot reproduce the results reported in Figure S6. I ran it on Chrome, Firefox and Safari (on macbook), but it still gives a blank page after submitting the query on default values. I recommend that the authors keep the web server up and running so that it can be (extensively) tested by others too.

5. One of the other drawbacks of the paper is the use of only one example to suggest the efficacy of the tool. The authors should it perform a similar analysis for other cancer or other disease types.

6. The authors suggest that the functional relationship between most of the SNPs and PrCa is

unknown and that PENGUIN (with the overlap analysis with PAINTOR results and other GWAS catalog datasets) can find the potentially causal SNPs. Can authors please validate (using CRISPRi as suggested by authors) these top candidates reported in S10 that has not been reported before? I would like to know whether these hits are true or false positives suggesting that the tool is useful.

6. That comes to the following query. Under the method section, the authors included the PrCA SNPs above the genome-wide p-value threshold. It is not a reasonable practice in GWAS analysis as this increases the false positive rate. Can the authors please include the number of SNPs that passed the threshold vs not, and how will this affect the results reported in table S10?

7. Can the authors please compare their results with the baseline and other tools, such as tools developed by Ratnakumar et al. and Dey et al. and focus the analyses around the EPIN regions? For baseline analysis, the authors can use the same datasets used to develop PENGUIN and do an overlap analysis suggesting that the mere intersection of datasets will have little power compared to a robust statistical analysis.

8. Another recommendation would be to use more examples like Myc in the main figure.

9. This comment is regarding the RNA-seq analysis performed. The authors of Tophat/Cufflinks have themselves suggested avoiding the use of Cufflinks for the quantification of gene expression. It is because FPKM (or RPKM for paired-end datasets) requires all the samples to have the same amount of mRNA/cell across all the samples, which in practice, does not hold. For quantification use RSEM, Kallisto (for transcript-level quantifications) or count file output from STAR are generally recommended. Both will output. Next, it is not recommended to use FPKM or RPKM units for DE analysis using DESeq2. If the author wants to perform the DE analysis using DESeq2, they should use the counts file.

Reviewer #1

Armaos and Serra et al. present a really interesting paper that presents a new integrative genomics method called PENGUIN to build protein-protein interaction networks with the additional regulatory layer of chromatin interactions. I think this is a really nice approach and I think the method has a lot of interesting applications to tease out disease mechanisms, especially when looking at variants from non-coding regions.

We thank the Reviewer for their valuable feedback and have taken each point into consideration as detailed below. We have revised the text accordingly, incorporating the suggested analyses.

Interesting method aside, the manuscript is reasonably difficult to read and can be much more concise. The writing itself is fine, however I kept asking "What is the actual focus on the paper?". The way I interpreted the manuscript was that it was actually two separate studies; one being the PENGUIN method and the other being the network of the prostate cancer cell-line. As far as I am aware, the manuscript does not introduce any new data from a previous study (Giambartolomei and Seo et al. 2021), so the real innovation is the PENGUIN method. If the paper is rewritten to focus on PENGUIN as a generalised approach, it would make it far more concise and easier to read. I would also suggest complimenting the analysis with an independent dataset (perhaps a non-cancer dataset) which would highlight the broad applicability of the workflow. There is a lot of data out there that could be used in a similar way as the prostate cancer cell-line. Of course, the opposite is also true, focusing specifically results as they relate to prostate cancer, however this would be less interesting to the general audience that Nature Communications aims to target.

We acknowledge and appreciate the Reviewer's observation regarding the need for clearer differentiation between the method and its application in the text. To address this concern, we have undertaken a comprehensive revision of the abstract, introduction, and conclusions sections. Additionally, we have restructured the results section and introduced a new figure (**Figure 1)** to clarify the input and output, introducing new subsections to enhance clarity and coherence. Additionally, to increase clarity of the work's focus, we have refined the manuscript's title.

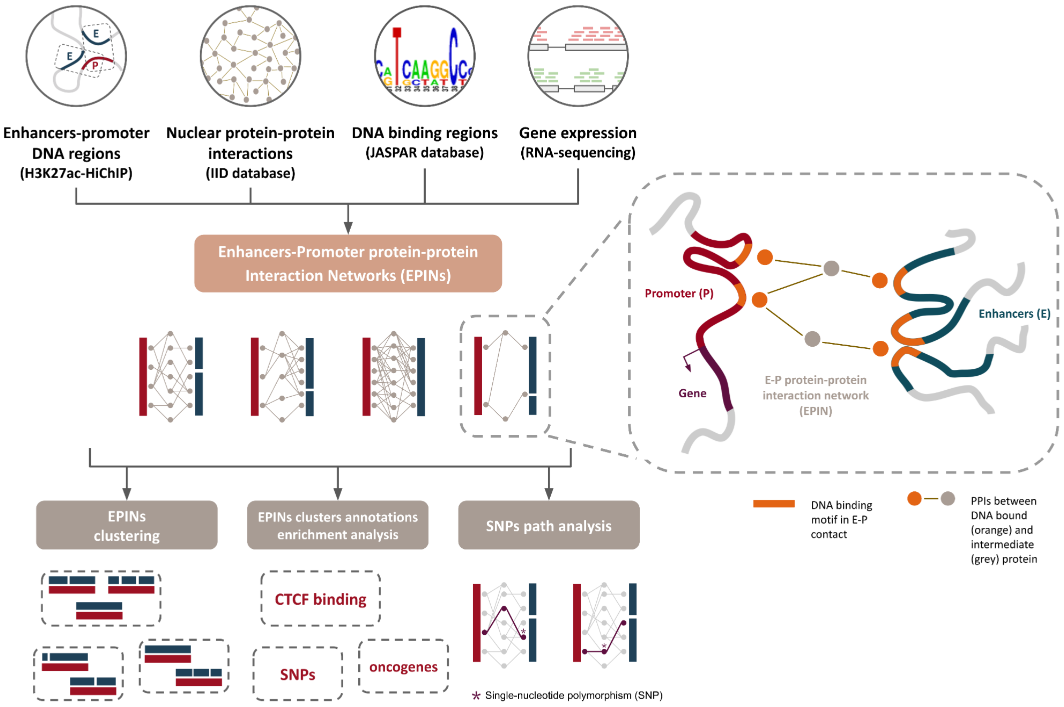

Figure 1. General overview of the PENGUIN workflow and downstream analyses. PENGUIN input consists of HiChIP data (in this work, H3K27ac in LNCaP or LHSAR cell lines), tissue-specific nuclear protein-protein interactions, PPIs (in this work, cancer and normal prostate PPIs from IID database), curated DNA-binding motifs (in this work, motifs from JASPAR database), and gene expression profiles (in this work, RNA-sequencing data in LNCaP or LHSAR cell line). PENGUIN output consists of Enhancer-Promoter protein-protein Interaction Networks (EPINs). Downstream analyses are designed to address specific questions related to prostate cancer (PrCa), namely the identification of clusters of promoters based on EPIN similarity, their enrichment in distinct annotations (CTCF binding from ChIP-seq peaks, PrCa-associated SNPs, and PrCa oncogenes), and finally the formulation of mechanistic hypothesis based on SNPs path analysis. In the inset, we report a schematic representation of an enhancer-promoter protein-protein interaction network (EPIN) reconstructed with PENGUIN for a given E-P contact detected by H3K27ac-HiChIP. Promoter and enhancer DNA binding motifs found in HiChIP regions after enhancer prioritization and the corresponding bound proteins are indicated in orange; their physical interactions with other factors of the EPIN (in gray) are represented as gray lines.

Focusing on the method, I have a few queries regarding the data and design decisions that was used to construct the networks: 1) *Why is hg19 being used for this analysis? You're missing a lot of very good enhancer information contained in hg38 resources. Given that most of the interaction methods were developed in their previous 2021 paper (Giambartolomei and Seo et al. 2021), I expect to see some innovation in the computational methods. While previous versions often have a place in clinical genomics, I see no reason to use hg19, especially given new regulatory element resources such as ENCODE.*

We agree with the Reviewer's point regarding the use of the hg19 reference genome in our analysis. While we initially opted for hg19 to facilitate quality control and comparison with other datasets generated by our lab, we recognize that starting the analysis with a more recent reference genome would have been preferable. However, it is important to emphasize that our study relies solely on enhancers defined by our

own HiChIP experiments, rather than relying on annotated enhancers or external definitions from ENCODE. As a result, any changes in regulatory element annotations would not impact our findings. Most importantly, it is true that hg19 may not encompass certain DNA loci containing enhancers. To assess this possibility, we conducted an analysis to determine the number of annotated enhancers in hg38 that would be lost if mapped to the hg19 reference genome. We obtained the complete list of annotated enhancers from Ensembl in hg38 (Ensemble Regulation v108) and performed the mapping using the UCSC liftover tool to convert them to the hg19 reference genome. Out of the 267,862 enhancers present in the hg38 resources, only 621 ended unmapped. It is worth noting that the majority of these unsuccessful conversions were labeled as "partial," "duplicated," or "split." This conservative estimate indicates that the mapping errors represent at most 0.23% of the total. Consequently, we are confident that the overall analysis is not significantly affected by the choice of an older reference genome.

We have added to **Methods**:

> It is important to emphasize that our study relies solely on enhancers defined by our own HiChIP experiments, rather than relying on annotated enhancers or external definitions from ENCODE.

2) *What is a promoter? I am not really a fan of taking non-informed ranges as the promoter, especially when there is some good promoter-specific information out there. Prostate cancer is very well studied so that information should be available. For example, could the authors use CAGE data from Takayama et al. 2015 instead? Would give a really good indication of which promoter is expressed and remove arbitrary length cut-offs such as 500bp.*

We thank the Reviewer for bringing up this point. H3K27Ac, utilized in the HiChIP method, is indeed a marker of active promoters and enhancers. We annotate the HiChIP loops from the promoter-perspective using the region of active promoters (from Chip-Sequencing data) that overlap promoter regions as defined by the RefSeq database. The objective is to subsequently functionally characterize these genes into EPIN networks based on associated protein interactions, rather than identifying active versus inactive promoters. We have clarified in different sections of the paper. For example:
In the **introduction**:

> ...high-resolution chromatin interaction maps enriched for a marker of active E-P activity (H3K27ac-HiChiP)…

In the **Results**:

> ...high-resolution chromatin interaction maps that capture active promoter-enhancer interactions, highlighting the dynamic nature of gene regulation…

3) *Gene expression is mentioned, but where is this coming from? I see that Giambartolomei and Seo et al. 2021 used FPKM that isconverted to transcripts per kilobase million (TPM), which I would have significant concerns with (use TPM from the start!)*

We thank the Reviewer for this remark. The expression data comes, indeed, from Giambartolomei and Seo et al. 2021 (doi:10.1016/j.ajhg.2021.11.007). In our new article the FPKM value is used as a filter in the PENGUIN pipelines to make sure that the genes we are considering are sufficiently expressed. To ensure that the proteins included in the network analysis had a minimum level of expression in the system, a gene

expression threshold was applied to filter PPI intermediates and DBP. The purpose of this step is to ensure that the genes encoding the proteins in the network show at least some level of expression. It is important to note that this threshold is not overly stringent, as demonstrated in Figure S4, panel C. In this study, we set the threshold at 0.03. We provide users with the flexibility to adjust this filter in the web interface, allowing them to increase the stringency if desired. We also provide the raw data in case the user would like to explore this aspect in a different analyses,
In order to clarify this point we added this sentence in the corresponding section of methods ("Gene expression data"):

> Depending on the dataset, this expression lower-bound may be modified in different use cases, for instance based on specific insights or based on a differential analysis between conditions. In this work, we used FPKM instead of more direct measures as we set our threshold very low and did not want to enrich our dataset with very long, virtually unexpressed, transcripts.

4) *In your analysis using PENGUIN you don't actually have a baseline because you are describing cancer/cell line-specific data. I realize this method is fine without a base-line comparison, but it would be interesting to see the contrast between a normal prostate tissue dataset? If similar interactions come up, then results would require a different interpretation*

We appreciate the insightful comment raised by the Reviewer. In response, we have made the decision to utilize a benign human prostate epithelial cell line (LHSAR) as our baseline for comparison with LNCaP cells. To ensure a comprehensive analysis, we performed HiChIP experimental data generation specifically for LHSAR cells, as there were no existing datasets available. Subsequently, we applied the PPI clustering procedure to further explore the functional relationships within the acquired data (**Figure 2**). To compare the network reconstruction between LNCaP and LHSAR, we employed hierarchical clustering to identify clusters of EPINs based on their edges. As the selection of an exact number of clusters in a given tree could be considered an important variable in our analysis, we examined various cluster numbers (4, 8, 16). The LHSAR HiChIP data were deposited in GEO and in our GitHub.

For LNCaP, the following features were used to annotate the clusters:

- CTCF experimental sites retrieved from ChIP-seq on LNCaP cell line from the ENCODE project
- Causal SNPs (95% confidence interval) from the fine-mapping tool Paintor on prostate cancer
- GWAS Catalog entries related to prostate cancer

Regarding LHSAR, the following features were utilized:

- CTCF experimental sites retrieved from ChIP-Atlas for prostate epithelial cells (due to the unavailability of exact same LHSAR cell line CTCF ChIP-seq data). Two distinct datasets, SRX3322103 and SRX3322104, were employed, along with their independently derived union of binding sites, with a stringency of q value <= 1e-10
- Causal SNPs (95% confidence interval) from the fine-mapping tool Paintor on prostate cancer
- GWAS Catalog entries related to prostate cancer

As an evaluation metric for the effectiveness of our approach, we used an independent set of known oncogenes in prostate cancer. Within a given clustering, we determined the number of clusters enriched in given features (e.g. GWAS SNPs and/or CTCF binding sites). Next, inside these enriched clusters, we

counted the fraction of EPINs whose promoter gene is a known oncogene. To normalize this fraction, we compared it to the expected fraction of oncogenes, which is calculated as the fraction of oncogenes within our total set of EPIN genes (refer to the figure below).

In the case of LNCaP, the partitioning in 8 / 16 clusters resulted in the identification of 1 / 2 clusters with a significant enrichment in oncogenes. **By contrast, in the control cell line LHSAR, a non-significant fraction of oncogenes was found only in 1 cluster out of 16 clusters, with enrichment in one type of signal in PrCa-associated SNPs (GWAS Catalog) and not in CTCF binding sites.**

Thus, we added to the main text:

> Our analysis did not reveal any cluster enrichment in GWAS and CTCF within the benign prostate control LHSAR. Moreover, we did not observe a significant increase in the number of identified oncogenes in LHSAR (**Figure 2B**). These results lead us to conclude that PENGUIN, along with the integration of intermediate PPI networks, significantly enhances the identification of candidate PrCa-related SNPs affecting key elements in chromatin architecture.

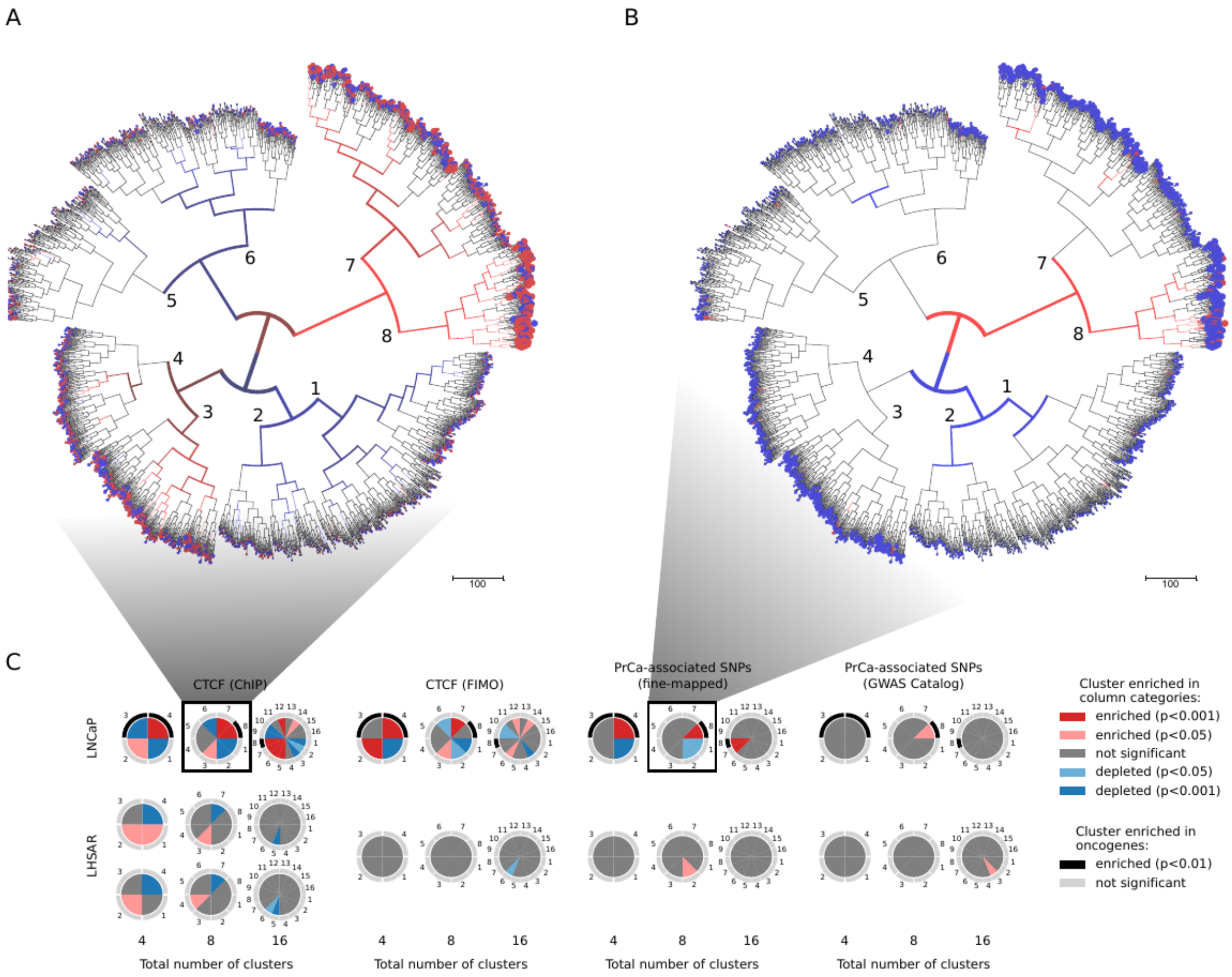

Figure 2. Clustering of the promoters originating the PENGUIN reconstructed EPINs. Clustering is based on edge composition of the EPINs. Leaf radius is proportional to network size. Color code (two-sided Fisher's exact test): red, enriched; blue, depleted; The figure is generated using ETE3 68. (A) Enrichment of PrCa SNPs in enhancers. We identified one PrCa SNP enriched cluster (GWAS+; cluster 8), and multiple PrCa SNP depleted (GWAS-; clusters 1, 2) and neutral (GWAS=; clusters 3, 4, 5, 6, 7) clusters. (B) Enrichment of CTCF ChIP-seq binding sites. We identified multiple CTCF enriched (CTCF+; clusters 3, 7, 8), depleted (CTCF-; clusters 1, 2, 6) and neutral (CTCF=; clusters 4, 5) clusters. (**C**) Clustering analysis on LNCaP (Top) and LHSAR (bottom) reconstructed EPINs. Pie-charts represent clustering results for a distinct total number of clusters used to partition the hierarchical clustering tree (4, 8, 16). Numbered pie-slices represent the different clusters and their color gradients encode the significance of enrichment (shades of red), depletion (shades of blue) or neutral (gray) of the overlap with distinct annotations (ChIP-Seq CTCF peaks, predicted CTCF binding sites by FIMO, PrCa-associated SNPs from fine-mapping and GWAS). Clusters significantly enriched with previously known oncogenes are annotated with black arcs. All enrichments have been estimated using two-sided Fisher's exact test.

We have added the following description to **Methods**:

> CTCF ChIP-seq peaks for LNCaP cell line were retrieved from ENCODE[51] project (https://www.encodeproject.org/) for the same Genome assembly, hg19 (GEO references: *GSM2827202* and *GSM2827203*). Overlaps of the CTCF binding sites with enhancer and promoter anchors allowed a 10 kb gap between them. Since CTCF ChiP-seq peaks for LHSAR cell line were not available in ENCODE, we retrieved from ChIP Atlas (https://chip-atlas.org/) two distinct sets (GEO references: *GSM2825573* and *GSM2825574*) of CTCF peaks (of same Genome assembly hg19) for prostate epithelial cells at a q-value of 1e-10 (Table 3). We used these two sets independently and in concatenation when comparing the clustering results between LNCaP and LHSAR. These narrow peaks were mapped on the enhancer regions using the python package *PyRanges* (see "E-P contacts" section). For both cases, LNCaP and LHSAR, the narrow peaks were considered as the CTCF binding sites.

5) *If the expectation is that DNA-protein/TF binding is implicated in disregulation of prostate cancer, then trans-eQTLs (as opposed to cis-eQTLs) would be a really interesting dataset to use for interpretation. trans-eQTLs are often driven by distant TF disregulation (Albert et al. 2018; https://elifesciences.org/articles/35471), rather than local (i.e. cis-) events. Based on your hypothesis, this would be a perfect dataset to include in your PENGUIN method*

We thank the reviewer for suggesting the use of *trans*-eQTLs. Indeed these are specific hypotheses we look for in the SNP paths analyses. Specifically, the reasoning behind a *trans* associations is that a SNP, located outside the gene encoding a protein close-by, can affect the expression of a distant gene, often located in a different chromosome, via chromosomal conformational mechanisms, through multiple mechanisms. For example, the SNP can alter microRNAs that in turn alter mRNA stability of a set of distant genes. One possible mechanism is that the SNP is located in genes encoding transcriptions factors that regulate expression of other physically distant genes, either as a direct consequence of the altered binding affinity due to the presence of a SNP (**Figure 3B**), or because an altered expression of a gene is altering in turn other genes within the biological cascade represented by the PPI link (**Figure 3C**).

For this reason, we note that we had reported pQTL associations in **Tables S10 and S11** describing SNPs in pathways, using protein levels (pQTL) instead of gene expression levels (eQTL) already, specifically:

EPIN_promoters_pval_pqtl: lists the pQTL p-value of the association of the SNP with the protein levels encoded by the EPIN_promoter
EPIN_promoters_seqids_pqtl: lists the identity of the proteins by SeqIDs for the association of the SNP with the protein levels encoded by the EPIN_promoter
Intermediate_pathways_pval_pqtl: lists the pQTL p-value of the association of the SNP with the protein levels encoded by the proteins in intermediates
Intermediate_pathways_seqids_pqtl: lists the pQTL p-value of the association of the SNP with the protein levels encoded by the proteins in intermediates
pqtl_proteins: lists the identity of the proteins by SeqIDs for the association of the SNP with the protein levels encoded by the proteins in intermediates

We now add specifically trans-eQTL hotspots comparison. In the analysis suggested by the Reviewer, we searched for enrichment within our GWAS+ cluster of SNPs altering many genes in *trans* ("hotspot regions"), to learn whether this mechanism was observed more in our cluster associated with PrCa. The paper suggested by the Reviewer is unfortunately on yeast, which exhibit different enhancer-promoter architectures from human genome (e.g. Kyrchanova et al., Int J Mol Sci. 2021 PMID: 33445415). So, we used the trans-eQTLs reported from the largest eQTL study available in humans (large-scale meta-analysis in up to 31,684 blood samples from 37 eQTLGen Consortium cohorts in Whole Blood) to be able to have power for a trans-QTL analyses, and looked at regions controlling more than 3 genes and defined these "hotspots". We did not find there was enrichment of hotspots in any particular cluster. While these results are intriguing, exploring them further was beyond the scope of this project.

We have added this to the main text:

Methods:
Trans-eQTL hotspots
We retrieved trans-eQTLs reported in the largest meta-analysis with up to 31,684 blood samples from 37 eQTLGen Consortium cohorts in whole blood in 22. We grouped enhancers by collapsing when they were separated by less than 20 kb, thereby creating 'enhancer clusters'. To qualify as a trans-eQTL hotspot, the enhancer clusters had to contain a SNP associated with at least 3 different genes. We quantified the normalized mutual information (NMI) between the hotspot-related enhancer clusters and our 8 EPIN clusters. In order to infer deviation from expected by chance and estimate an empirical p-value, we randomized 10 thousand times the association between each enhancer and its corresponding EPIN cluster and computed the NMI between each randomized EPIN clustering and the observed hotspot-related enhancer clustering. Additionally, we checked if a given cluster was significantly enriched in trans-eQTL hotspots. For this purpose we applied a Fisher test to our pool of enhancers comparing the two contingencies, inside/outside a given cluster, and inside/outside a trans-eQTL hotspot.

Results
To explore the potential connection between our clustering approach and the presence of trans-eQTLs, we used the trans-eQTLs reported from the largest eQTL study available (large-scale meta-analysis in up to 31,684 blood samples from 37 eQTLGen Consortium cohorts in Whole Blood, 22) and defined a region an 'eQTL hotspot' when associated to more than 3

genes (**Methods**). We observed an enrichment of eQTL hotspots across all clusters (**Figure 5SE**, empirical p-value <0.0001), but not specifically for cluster PrCA GWAS+ (**Figure 5SF**).

Accordingly, we added **Figure 5S:**

E

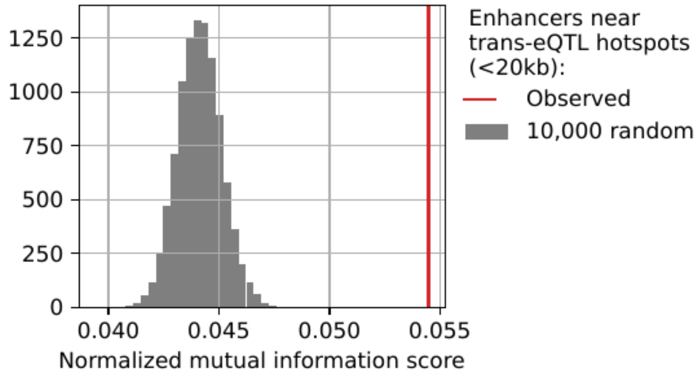

F

Cluster number	Odd-ratio	p-value
cluster 1	1.1	0.65
cluster 2	1	1
cluster 3	0.8	0.35
cluster 4	1.2	0.38
cluster 5	1.2	0.61
cluster 6	0.7	0.2
cluster 7	1.1	0.58
cluster 8	0.8	0.42

Figure S5 (**E**) Relationship between trans-eQTL hotspots and the 8 clusters using the concept of normalized mutual information. We focused on enhancers derived from our EPINs, which were associated with trans-eQTL hotspots located within a proximity of less than 20kb. A relatively weak correlation coefficient of 0.0546 if found between the 8 clusters and the hotspots defined by their proximity to trans-eQTL hotspots. Randoms were generated by shuffling the association between enhancers and EPIN clusters. (**F**) We investigated whether a specific cluster exhibits a significant enrichment of trans-eQTL hotspots. For this employed a Fisher test, comparing two contingencies within our list of enhancers: those within or outside a given cluster, and those within or outside a trans-eQTL hotspot.

6) *Correct me if I am wrong, but GWAS catalogue variants are lead-SNPs and therefore indicate a "region" of association, rather than fine-mapped SNPs? I would caution using those SNPs as a basis for looking at enrichment. If a PrCa GWAS is available, then assessing polygenic risk would be a nice way of prioritising important variants? Or perhaps taking all variants within the GWAS SNP region and using a more targeted association test?*

We apologize for any confusion caused by our use of GWAS SNPs in different contexts.
We agree with the reviewer that lead SNPs represent genomic regions. For this reason, we had utilized PrCa SNPs within a credible set obtained after fine-mapping 137 previously associated regions using PAINTOR, a probabilistic method that incorporates GWAS summary statistics and LD structure to provide a confidence set of SNPs which are more likely to be causal. To support our conclusions on enrichments of PrCa annotations as a sensitivity analysis, we have used GWAS from another dataset passing a genome-wide p-value threshold. We have clarified that this is used as an annotation and in no way intended to be an exhaustive list of causal SNPs for PrCa. This approach contextualizes a SNP that has been previously associated with PrCa with any method of choice within the E-P looping realm.

The method we report in this paper, PENGUIN, identifies enrichment of these fine-mapped SNPs in a particular cluster. To identify potential SNP paths (genes and networks) affected by the two specific hypotheses (**Figure 3B,C**). We use the information on association of SNPs from fine-mapping as an annotation to our clusters (to say it explicitly, we look for annotations in defined regions, either enhancers with a TF binding site or gene bodies of specific intermediates, and link to other genes in a network). We tested specifically the correlation (Pearson correlation) between the number of PrCa SNPs we have analyzed and the number identified from Tables S10 and S11. There was no significant correlation.

We thank the reviewer for the suggestion of the targeted approach and have clarified the text accordingly. Specifically, we clarified different sections of **Method** and have added the suggested specification in **Discussion**. We have added **Figure S11** illustrating the location of the SNPs we identify in our analyses (Tables S10 and S11).

In **Methods**:

PrCa SNPs

To explore enrichment of SNPs associated to PrCa across the identified clusters, and to identify the SNP paths, we used the previously reported 95% credible set[11] from fine-mapping 137 previously-associated PrCa regions using a Bayesian statistical method PAINTOR [59] employing the largest PrCa genome-wide association studies (GWAS) (N = 79,148 cases and 61,106 controls)[60]. This set was composed of 5,412 distinct SNPs (rsid). We will refer to these as PrCa SNPs. Note that this set also includes SNPs that do not reach genome-wide-filters of p-value significance. We illustrate the location of the associated PrCa regions and number of PrCa SNPs in **Figure S11**. We did not find a significant correlation between the number of PrCa SNPs in the regions and the number of PrCa SNPs we prioritized in this work (Pearson r=0.2, p-value=0.06 and Pearson r=0.1, p-value=0.3 for **Tables S10** and **S11**, respectively). We mapped the SNP location to prioritized enhancer regions anchor locations with a window of 10 kb. 518 out of 5,412 overlap our prioritized enhancer regions; 18 of them overlap our promoter regions. In total 218 prioritized enhancers and 14 promoters overlap a PrCa SNP.

In **Discussion**:

In this work, we use a targeted approach and use the information on association of SNPs from fine-mapping as an annotation to our clusters. Specifically, we identify potential SNP paths (genes and networks) in defined PrCa associated regions, either enhancers with a TF binding site or gene bodies of specific intermediates, and link to other genes in a network. This approach contextualizes a SNP that has been previously associated with PrCa within the E-P looping realm.

7) *I need more convincing when it comes to the enrichment of CTCF binding sites in the network. As mentioned above, GWAS SNPs used are unlikely to indicate any disease association so enrichment in CTCF binding sites may not have the biological impact that you might expect. Also, CTCF binding sites are everywhere, but *some* are involved in domain formation. Further classifying CTCF sites to ones that are likely to impact regulation or 3D structure would lead to more intepretable results.*

We thank the Reviewer for this comment; this is a very important point that we hope has now been clarified in the text. In our study, we utilized CTCF ChIP-seq peaks (within a 10kb window) in the LNCaP cell line.

Specifically, we focused on CTCF peaks that are symmetrically present in both a promoter region and an enhancer region, exhibiting significant chromatin interaction. This approach allows us to target CTCF bindings that are likely involved in the formation or maintenance of chromatin loops. While it is true that CTCF can be recruited for various structural purposes, such as promoting promoter-enhancer contacts or insulating genome regions, our methodology is designed to specifically capture CTCF sites associated with chromatin looping (the H3K27ac used in our HiChIP is an epigenetic mark associated to both active promoter and active enhancers). This very directed approach reduces the possibilities of alternative functions for our CTCF dataset.

To address these previously unclear points, we have now modified the corresponding **Method** section to highlight two key aspects: our utilization of lineage-specific CTCF peaks and our definition of CTCF-positive EPINs, which require the presence of a CTCF peaks at both ends of the enhancer-promoter loop:

> For the enrichment in CTCF binding, we used CTCF peaks from an external dataset but in the same cell line (see **CTCF ChiP-Seq peaks).** We considered an EPIN to be CTCF-positive (CTCF+), if a CTCF peak was found in a 10 kb region around its promoter and around 10kb of at least one of its enhancer regions.

We have also replaced several mentions of “CTCF binding sites” by “CTCF peaks”.

8) *What is the background/universal set of genes/proteins that you are using for gene set enrichment? Gene-set enrichment ALWAYS picks up cancer-related genes. This is because most of the gene-sets are developed from cancer gene expression sets, so there is a massive bias towards them. A control analysis, such as the one suggested above with 'normal' prostate cancer data, would be more convincing.*

We agree with the Reviewer on the importance of the background set of proteins for the gene set enrichment. Previously, we used the default configuration of g:Profiler for this matter (i.e. ‘Only annotated genes’), which as stated by the Reviewer may introduce a bias towards cancer-related genes. We have now changed the background of the analysis presented as **Table S9** to the very limited set of 751 unique intermediate proteins forming the promoter networks. Accordingly, we have also changed the background of the analysis presented as **Table S5** to the 4314 genes associated with the clustered EPINs. The choice of such backgrounds has been made following suggestions from the original publication of the g:Profiler web service (Raudvere et al. 2019):

> *‘"By default, g:GOSt uses the set of all annotated protein-coding genes as a background. In some experiments however, only a subset of genes or proteins is measured. For example, the targeted sequencing of only disease specific genes would imply enrichment of that disease association. Statistically, for these cases it is recommended and sometimes necessary to use custom background information when calculating the statistical enrichment significance. The custom background should include a list of genes that were actually measured during the biological experiment, such as all genes in the sequencing panel. This option allows us to calculate a more precise evaluation of functional enrichment."*

Even considering such a limited background for the analysis presented in **Table S9**, we obtain meaningful enrichments (with lower statistical significance values as a result of changing the background) in GWAS+ cluster (cluster 8):

> KEGG Prostate cancer pathway (KEGG:05215) appears highly enriched (adjusted p-value = 1.27e-2) together with other pathways related to tumors such as Colorectal cancer (KEGG:05210,

adjusted p-value = 3.20e-5) Pancreatic cancer (KEGG:05212, adjusted p-value = 9.54e-4) and Breast cancer (KEGG:05224, adjusted p-value = 7.06e-4). KEGG pathway KEGG:04919 (Thyroid hormone signaling pathway) is an additional highly enriched pathway (adjusted p-value = 2.57e-4). Thyroid hormones have been previously described as modulators of prostate cancer risk [24–27]. Pathway KEGG:05200 (called Pathways in cancer) appears as the fourth most enriched KEGG concept (adjusted p-value= 3.63e-4). Other classical tumorigenic pathways, such as Wnt signaling pathway (KEGG:04310, adjusted p-value = 1.27e-2) and TGF-beta signaling pathway (KEGG:04350, adjusted p-value = 8.21e-4) appear to be enriched. In this regard, recent studies analyzed the involvement of Wnt signaling in the proliferation of prostate cancer cells [28,29], as well as the involvement and TGF-beta signaling [30,31].

Furthermore, we examined the functional enrichment of significantly central proteins across all other clusters. This analysis was conducted to facilitate functional comparisons across different clusters (**Methods** 'Functional gene set enrichment analysis'). This analysis revealed no enrichments for clusters 1, 2, 4, 5, and 6 (cluster 5 does not have significantly central proteins). This observation can be attributed to the higher number of central proteins in these clusters (365 in cluster 1, 283 in cluster 2, and 318 in cluster 6) compared to the other clusters (3 in cluster 3, 7 in cluster 7, and 22 in cluster 8). Despite having a similar number of significantly central proteins to cluster 8 (30 proteins), cluster 4 does not show any enrichment.

Moreover, of the clusters presenting enrichments (i.e., clusters 3 and 7), only cluster 7 presents enrichments related to those observed in cluster 8 (for example, KEGG prostate cancer pathway is enriched, adjusted p-value = 2.041e-2; **Figure S8**). As commented, cluster 7 presents only 7 significantly central intermediate proteins (CREBBP, CTNNB1, GSK3B, KAT5, MAPK1, PIN1, SMAD2), out of which, 6 overlap with those significantly central in cluster 8 (only PIN1 is absent).

Overall, we understand the concerns of the Reviewer on the importance of the choice of background for the gene set enrichment analysis and we updated the configuration of the analysis accordingly to avoid such an obvious potential bias. We thank the Reviewer for the suggestion.

Overall, I think this is a really nice application of a hypothesis-driven multiomics integration workflow, which is surely needed in the current literature. I hope these suggestions help improve the manuscript before publication

We thank the reviewer very much indeed.

Reviewer #2

This manuscript established a new approach, called PENGUIN, which was used to identify protein-protein interactions (PPI) in E-P interactions in prostate cancer. Indeed, the authors integrated H3K27ac-HiChIP with a tissue-specific PPI network and gene expression to design this novel approach. This study has presented a good research tool for scientists. Through this tool, key factors that may play a role in transcriptional regulation of prostate cancer will easily be confirmed , and distinct molecular cascades potentially affected by prostate cancer SNPs at E-P contacts can also be identified , opening up new directions to identify molecular targets for disease treatment.

We thank the reviewer for the positive support on the manuscript and PENGUIN tool.

Reviewer #3

Armaos et. al. presented a method PENGUIN to identify the PPI interactions supported by HiChIP data (which the authors termed as EPIN) and perform unsupervised clustering to identify a group of promoters with similar EPINs. They observed the enrichment of PrCa-specific GWAS SNPs in the identified P-E networks, particularly in cluster 8. The approach is quite interesting and promises to be useful. However, the utility of the PPI integration and the output causal genes and SNPs need to be thoroughly benchmarked with the existing studies. I, therefore, request a major revision of the manuscript addressing the following comments.

We thank the Reviewer for the nice remark. Following their suggestions we have carried our new analyses and modified the text accordingly.

Major Comments

1. The authors used 5 Kb as the lower threshold of interaction distance. Usually, HiChIP or Hi-C interactions below 10 Kb are not reliable. What fraction of HiChIP interactions and what fractions of DNA binding proteins in PPI are within 10 Kb? Does a change of this distance (from 5 Kb to 10 Kb) alter the proposed PPI-based clustering, specifically cluster 8?

We thank the Reviewer for pointing this out. We called HiChIP interactions at a 5 kb resolution as previously described (Fulco et al. 2020, doi:10.1038/s41588-019-0538-0; Giambartolomei and Seo et al. 2021, doi:10.1016/j.ajhg.2021.11.007; Bhattacharyya et al. 2019, doi:10.1038/s41467-019-11950-y) and then extending these interacting 5 kb bins by 5 kb on each side, resulting in the mentioned 15 kb bins. Thus, when we define that interacting fragments should be at least 5 kb apart, from the point of view of the originally detected HiChIP interaction, this means a distance of 15kb (5kb apart plus twice 5kb for each fragment). The construction of our 15kb interacting bins was explained in the first section of the Methods, but we have now added a sentence to relate it with the minimal distance allowed between interacting fragments:

> We note that the enhancer anchors at this stage of the analysis are of length 15 kb, due to 5 kb resolution of the HiChIP data analysis and additional 5 kb padding added to anchors on either side.

2. The authors used a separate normalization scheme (Fig. S5) compared to ICE. The authors need to elaborate on the normalizing equations in Fig. S5 and also compare ICE and the proposed normalization scheme in terms of the Pearson correlation between genomic distance and normalized contact count.

We thank the Reviewer for this comment. Our primary objective is to optimize the normalization strategy while maximizing the utilization of our dataset. This objective becomes particularly challenging due to the unique characteristics of our dataset. Our dataset comprises, on the enhancers sides, 15 kb regions divided into smaller 1 kb regions. While on the promoter side, our specific focus is on the 1 kb region surrounding the promoter (TSS-1kb). To effectively normalize the data using methods like ICE or other known normalization strategies, we face the requirement of averaging the biases observed in the two bins that overlap with our promoter regions (as the 1 kb of the promoter region will not exactly match the 1kb genomic binning). ICE or related method have another flaw regarding our dataset, it assumes that all bins in the genome should have the same total amount of interaction. This assumption cannot be satisfied in our HiChIP dataset. It is true that our interaction matrix can be filtered considering only bins with enough signal, and ICE biases can be computed in this reduced genomic matrix. However our normalization does not need a global genomic scope, PENGUIN only needs 1 kb prioritized bins within each enhancer independently

from observations in other parts of the genome. These are the considerations that brought us to implement our new *ad-hoc* normalization strategy taking into account the specific characteristics of datasets and protocol .

We employed TADbit 1 (Serra et al. 2017) to run ICE normalization on our Hi-C data at a resolution of 1 kb, applying a filter to exclude bins with less than 100 di-tags. The choice of bin filtering is crucial for ICE normalization, as setting it too low may include sparsely populated regions in the Hi-C matrix, while setting it too high may excessively filter out bins of interest. In our case, we opted for a relatively low filtering value (1,000 is recommended - Rao et a. 2014,), which resulted in the loss of approximately half of the bins we initially targeted. Despite this, the resulting matrix remained too sparse for the ICE algorithm to converge adequately, with a convergence score fluctuating between 3 and 6 after 100 iterations, instead of the expected value below 0.0001 - (Imakaev et al. 2012). To address these challenges, we performed ICE normalization using various filtering values, ranging from 50 counts per bin to 1,000 counts per bin. After careful evaluation, we determined that setting the filter to 100 di-tags per bin provided the best trade-off between sparsity, the number of included bins, and the correlation with genomic distance. Additionally, we utilized the ICE biases per bin to estimate expected interaction counts. These biases were averaged over the two bins overlapping with the 1 kb promoter region and over the 15 bins within the 1 kb enhancer region (numpy *nanmean*). This approach enabled the rescue of a substantial number of enhancers that would have otherwise been discarded. We observed notable improvement compared to the raw data, with a correlation with genomic distance that is comparable to our normalization method. Notably, by varying the amount of included data, not averaging ICE biases, or employing stricter bin selection criteria, we achieved higher correlations. For instance, by maintaining the same filtering threshold of 100 di-tags per bin but not averaging biases, the correlation increased to 0.65. However, it is important to highlight that such approaches resulted in a rapid loss of data, ranging from 70% to 87% of the data utilized in our normalization method. Finally, the results are shown in **Figure S1E**. We observe an improvement with respect to the raw data, and a value of correlation with genomic distance comparable to the one from our normalization. As mentioned we tried different values for filtering low-count bins in the matrix. Our specific normalization was showing almost always a better correlation with genomic distance, and a much higher number of usable regions (see image below)

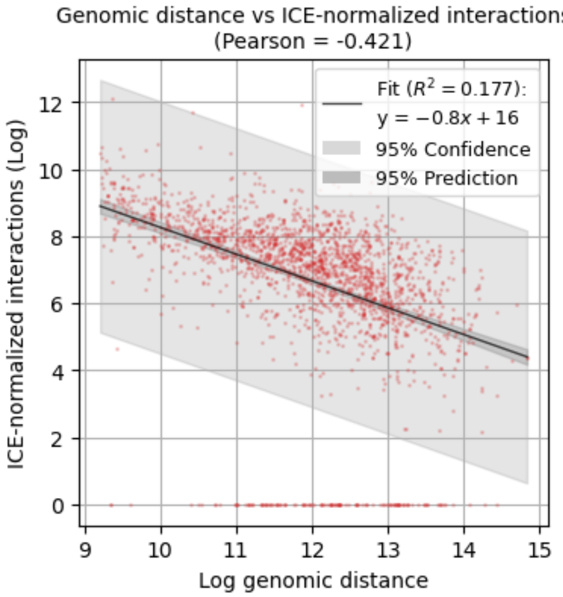

Correlation between ICE-normalized data and log genomic distance as in ***Figure S1E*** *but using only bins with data, without averaging over neighboring bins.*

Note that we tried the ICE normalization with several values of filtering ranging from 10 interactions/bin to 1,000 interactions/bin, and the best trade-off in terms of sparsity *vs* number of included bins (as well as the correlation with genomic distance) was found when setting this filter to 100 di-tags per bin (see figure below and **Figure S1E** showing only the best tradeoff state).

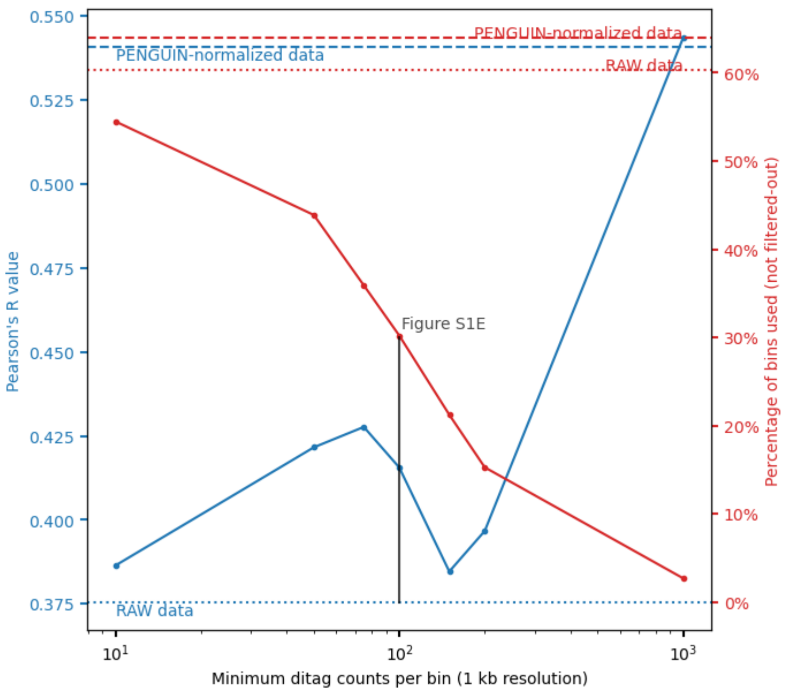

Comparison of different results of ICE normalization with different starting dataset depending on the filtering of bins in the interaction matrix, from requesting a minimum of 10 di-tags per 1kb bin to 1,000. Left, blue, axis and plots show the Pearson correlation between log genomic distance and the log number of interactions (normalized by ICE full line, normalized by our PENGUIN protocol dashed line, or not normalized dotted line). Right, red axis and plots show the percentage of data kept for analysis after bin filtering and selection of relevant P-E pairs.

In the best configuration of the ICE normalization (**Figure S1E**) we observe an improvement with respect to the raw data, and a value of correlation with genomic distance comparable to the one from our normalization. As mentioned we tried different values for filtering low-count bins in the matrix. Our specific normalization was showing almost always a better correlation with genomic distance, and a much higher number of usable regions (see image below).

To reflect this study (and we thank the Reviewer for the suggestion), we added this paragraph in the corresponding methods section:

> In order to compare with standard normalization procedure we applied the ICE normalization52 to our dataset (using TADbit[55] 1 kb resolution; filtering bins with less than 100 di-tags - 75% of the genome lost even using a threshold 10 times below the recommended[53]). Because of the sparsity of the genomic matrix the normalization did not fully converge (ICE was not able to completely balance the average di-tag counts per bin[52]). Next we applied the following normalization to our loops dataset, with few modification in order to rescue as much signal as possible: 1- in the promoter site, as our definition of promoter is exact (TSS to TSS +1 kb), we corrected using the

average of the two bins spanning over this 1 kb region 2- on the enhancer site, as most of the 1 kb loci were excluded by the normalization filter we also averaged the ICE bias over the whole region. Even with these modifications, only half the original data was recovered. However the correlation between genomic distance and number of interactions was significantly improved with respect to raw data. Overall, the correlation value observed with ICE was similar to the one measured for our normalization (**Figure S1E**). We believe however that, for this dataset and for our methodology, our normalization procedure represents an improvement as it considers the exact promoter regions (not partially overlapping 1 kb bins) and minimizes the loss of promoter-enhancer data.

The normalized profile of interactions was finally used to prioritize most interacting 1 kb loci on the 15 kb enhancer (**Figure S1F**). The selected 1 kb regions are referred to as prioritized enhancer regions.

Besides the comparison with ICE we have just explained, we have also done extra analyses, not shown in the manuscript, which consist in the comparison of CTCF enrichment in prioritized EPINs with the complete EPINs (no *ad-hoc* normalization and prioritization). In both cases, we divided the EPINs into 8 clusters and considered the cluster showing the most enrichment in CTCF. While the most enriched cluster using the prioritized EPINs showed an Odd Ratio of 3.29 (p-value=3e-20), the most enriched cluster using data considering all enhancer region (no normalization and subsequent prioritization) had a Odd Ratio of 1.34 (p-value=8e-4).

3. It would be interesting to see if the authors employed clustering using HiChIP interactions only and not using the reference PPI and binding proteins. Do the highly connected cluster (and the P-E nodes) from the HiChIP interactions highly overlap with the proposed GWAS+ cluster 8? It may highlight the contribution of the reference PPIs in identifying the functional HiChIP interactions.

This is an excellent point that allows us to actually show the power of our approach. Accordingly, we repeated our clustering, this time, based solely on the list of enhancer IDs (names based on their genomic coordinates) in each EPIN (**Figure S7**). These results show that the use of intermediate PPI networks increases the number of oncogenes identified. Thus, the information conveyed by the PPI network is relevant for EPIN classification and to their association with phenotype.

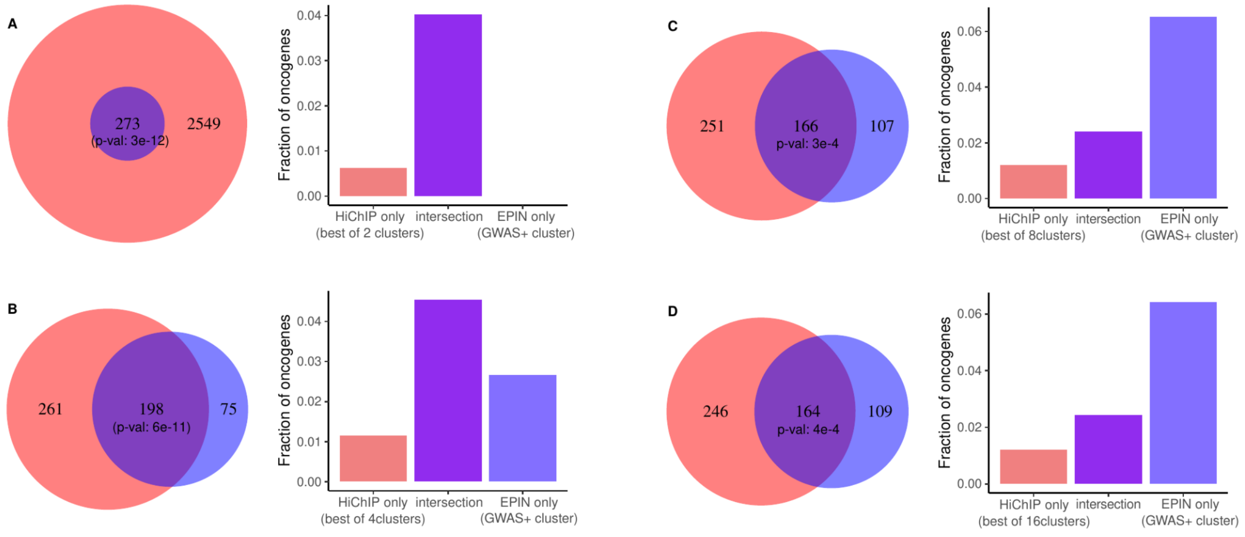

Figure S7. Comparison between clustering based on full EPINs (blue) and using only HiChIP data (no intermediate PPI network) (red). For each clustering strategy, only the cluster most enriched in PrCa SNPs and CTCF peaks is used in the comparison. The comparison is conducted in terms of the proportion of known PrCa oncogenes in the two sets, considering various cluster numbers within the red set (2, 4, 8, and 16 clusters), and only one cluster set (8 clusters for the blue set). Each panel (**A-D**) illustrates a Venn diagram showing the intersection (purple) between the red set and the blue set, and the corresponding fraction of oncogenes as a bar plot. The fraction of oncogenes that are unique to the red set ("HiChIP only") is consistently lower than the fraction of oncogenes that are unique to the blue set ("EPIN only"). Moreover, when compared with 8 and 16 clusters of the red set, the fraction of oncogenes of the "EPIN only" subset is higher than the intersection, indicating a relative gain in oncogenes retrieval when PENGUIN is employed. The significance of the intersection was estimated with a hypergeometric test considering the union of the two sets as the background.

4. Table S3, Figs. S1 and S2 show that cluster 8 contains the lowest promoter nodes but the highest number of edges, higher average enhancer connectivity, and higher enhancer-binding proteins. The proposed clustering approach, in effect, identifies highly connected regions. I request the authors to add the following information for cluster 8:

a. Whether the enhancers in cluster 8 are actually parts of one or more super-enhancers.

Our analysis primarily focuses on the promoter region, which serves as the anchor for defining EPINs. While some enhancers within an EPIN may belong to a super-enhancer cluster, each enhancer is identified based on chromatin contact detected through HiChIP. It is worth mentioning that even if some of our enhancers exhibit characteristics of super-enhancers, our specific definition ensures that enhancers are distinct regions of approximately 15 kb, with a minimum separation of another 5 kb. Consequently, it becomes unlikely for a super-enhancer to span across multiple enhancers within our dataset. We have shown this in **Figure S4G**.

Detailed information regarding the number of enhancers per EPIN promoter can be found in **Table S1** that have been used to generate **Figure S4G**.

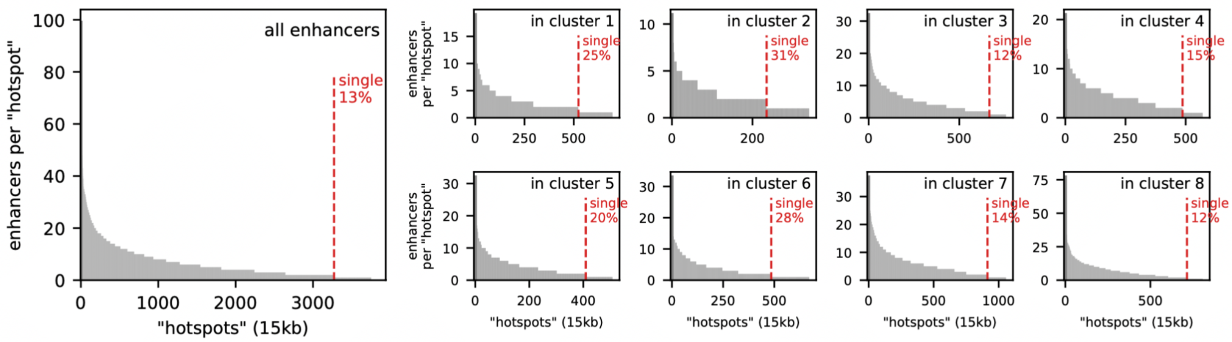

Figure S4G. Number of prioritized enhancers per enhancer hotspots. Hotspots are defined as groups of enhancers separated by less than 15kb. Dotted red line shows the proportion of enhancers that are isolated. The different panels show enhancers in the whole genome (left), and in each of our 8 defined clusters (smaller panes on the right).

We have added to the main text:

> We then assessed whether PENGUIN clustering was influenced by super-enhancer-like regions sharing target promoters in given clusters. Although the distribution of enhancers per hotspots is similar among our 8 clusters (**Figure S4G**), the GWAS+ cluster has fewer single enhancers (enhancer at more than 15 kb from any other enhancer). The average number of promoters targeted by each hotspot for all our 3,752 defined enhancer hotspots was 1.83 promoters targeted per hotspot. When measured considering only the promoters in given EPIN clusters, the values were: 1.29 for cluster 1, 1.28 for cluster 2, 1.25 for cluster 3, 1.24 for cluster 4, 1.22 for cluster 5, 1.21 for cluster 6, 1.34 for cluster 7 and 1.27 for cluster 8. In this case, values were very similar between EPIN clusters.

b. Whether the promoters and enhancers in cluster 8 highly overlap with significant GWAS SNPs (p-value < 5e-8; without any fine mapping).

Accordingly, we performed the cluster Fisher test enrichment and found that Cluster 8 exhibits significant enrichment in GWAS (p-value < 5e-8; without any fine mapping). The odds ratio is 4.24, with a p-value of 1e-06. The GWAS SNPs list comprises a total of 20,155 SNPs.

We thank the reviewer for this remark and have added this analysis to **Methods** and **Table S3B**.

5. The authors need to show the overlap of causal SNPs and genes reported by PENGUIN with their previous approach to identifying causal genes and SNPs from HiChIP (Giambartolomei and Seo et al. 2021). Although they have mentioned putative causal loci and genes (page 10), a comprehensive comparison would be useful.

Table S7 from Giambartolomei and Seo et al. identified 665 genes with H3K27ac HiChIP in prostate cell lines identifies risk genes for prostate cancer susceptibility: The American Journal of Human Genetics Accordingly, we performed comparative analyses of genes and SNPs identified through HiChIP and PENGUIN methodologies. In terms of genes, we found an overlap of 23 genes, considering a total of 665 genes from HiChIP, 34 genes from PENGUIN, and a background of 4,314 EPINs. This overlap demonstrated a significant enrichment of 10^{-11} (hypergeometric test), indicating strong agreement between the two approaches. Regarding SNPs, we observed an overlap of 17 SNPs, considering 1,818 SNPs identified through HiChIP and 36 SNPs identified through PENGUIN, using a background of PAINTOR SNPs. This overlap also showed a significant enrichment of 0.05 (hypergeometric test), further supporting the concordance between the two methods. Notably, the PENGUIN analysis reveals additional SNPs present in intermediate nodes (**Table S9**) that cannot be identified through HiChIP alone, demonstrating the added value of the PENGUIN approach. These findings highlight the complementarity and enhanced sensitivity of PENGUIN in capturing genetic variations within the regulatory landscape compared to HiChIP alone.

6. The authors need to benchmark PENGUIN in terms of the causal genes and SNPs reported with the existing approaches for prioritizing genes and SNPs (ABC score, Dey et al. 2022).

We thank the Reviewer for raising this point. The Activity-By-Contact (ABC) model links an enhancer region to its supposed target gene. This is done through a metric that links Hi-C interactions with the H3K27ac mark obtained from a ChIP-seq experiment. The ABC score thus allows to filter Hi-C interactions using H3K27ac marks and to focus on interaction potentially occurring between active promoters and active

enhancers. In order to verify the sensibility and specificity of their ABC-score the authors have benchmarked against HiChIP-H3K27ac and found that experimentally tested enhancer–gene interactions H3K27ac ChIP-seq for the ABC model performs similarly as having H3K27ac ChIP-Seq (Figure 3a in Fulco et al.). Thus, the HiChIP-H3K27ac, the experiment that we are using here, is the experimental alternative to the computation of the ABC-score. In other words, we are working on experimentally validated Interactions between histones with H3K27ac marks, while ABC is computationally predicting interactions in a cell-type.

We did nonetheless check whether the genes we prioritized are also contained within the published results of the ABC model. We considered the genes covered in both ABC and PENGUIN (3,619). Interestingly, 25 out of 29 genes from Table S10 considered in both analyses were identified in both PENGUIN and as positive predictions of the ABC model (ABC score ≥ 0.022), similarly, 9 out of 10 intermediate genes from Table S11 were identified in both the analyses, indicating a high concordance between ABC and enhancer-promoters from HiChIP, also as previously reported in Giambartolomei and Seo et al.

For the SNP comparison, we could not find a proper set for comparison with the methods mentioned here. We could not directly use the original Dey et al., the author who devised SNP-to-gene linking strategies (including the ABC score from Fulco et al.), because SNPs were not assessed other than for only a small set of phenotypes associated with autoimmune disease and blood traits. The follow-up paper, Gazal et al. with Dey as co-author ("Combining SNP-to-gene linking strategies to identify disease genes and assess disease omnigenicity", Gazal et al.), links a SNP to disease using multiple epigenetic information across different cell lines outside of our focused hypothesis of cell and disease specific enhancer-promoter CTCF-mediated pathways, so we do not believe is an appropriate comparison for our results. For this reason, we added validation of the enhancer region containing the PrCa SNPs using CRISPR (**Figure 4).**

We have added to the **Discussion**:

> For example, we have used as input enhancer-promoter loops cell-specific H3K27Ac HiChIP experiments (strict calling of loops and prioritization), to maximize our true positives in the input data. The input for the PENGUIN clustering approach can also be constituted by enhancer-promoter links measured from other experimental methods aside from HiChIP or even using computational methods. We leave this for subsequent analyses.

7. On pages 9-10, the authors mention that identifying DEGs even in intermediate nodes is useful, while some known oncogenes (like MYC) may not be DEG. Given that such biological significance of many genes may not be known, the authors need to formally characterize the set of genes to be used after DEG analysis.

We believe the reviewer is referring to the following statement: "Finally, at the level of intermediate proteins, we also found some encoded by genes reported to be differentially expressed. We observed that the mean proportion of intermediates that are differentially expressed is on average 40% (**Figure S2**)." However, it seems that this point may not be of significant concern to us. While we do observe enrichments in oncogenes, in this context, we are simply stating that we observe an enrichment in differentially expressed genes (DEGs). We are not implying that all DEGs are oncogenes or expecting to find them exclusively in that category.

8. The web server mentioned is not loading.

We apologize and have fixed a problem on the Virtual Machine.

9. The authors need to describe the column names in individual supplementary excel sheets. The fields are not properly explained.

Thank you, the labels are now in the Supplementary Materials.

Minor Comments

1. Page 8, lines 273-280: Figs. 1A-1C should be referred to as Figs. 3A-3C.

We have changed that in the new version of the manuscript.

2. Table S3, 2nd column: n_networks while the 2nd column in Table 2: number of genes – contradiction.

We have changed that accordingly.

Reviewer #4

The manuscript by Armaos et al. presents an interesting tool called PENGUIN (Promoter Enhancer Guided Interaction Network) which integrates HiChIP-H3K27ac, tissue-specific protein-protein interaction network, gene expression and TF binding motif datasets. However, there are some issues that need to be addressed. They are as follows:

We thank the Reviewer for acknowledging that the PENGUIN tool is of interest. We hope to have provided compelling evidence on its usefulness by replying to the points raised.

1. Since most of the downstream and comparative analyses were performed on the clusters, it is essential to discuss and address the robustness of the clusters. It needs to be clarified how the number of clusters or k was chosen after hierarchical clustering was performed, i.e., how they came up with 8 clusters in Figure 1/Figure S1A? Typically the number of clusters depends upon where the cut is performed on the dendrogram. So, please elaborate on how this decision was made. Were the models compared to other k?

The hypothesis of the manuscript is that EPIN structures are relevant to biology. Either as a biological marker, or because they do represent a specific biological function. To answer this question we have performed an exploratory analysis without prior on what number of clusters to expect. This analysis is based on hierarchical clustering, and in this analysis, we observed that we started to find significant enrichment for CTCF in EPINs when our dendrogram was split in 8. Following our analysis we then observed that this split also allows us to detect significant differences for relevant features, like associated SNPs or oncogenes. Thanks to the comments of this and other reviewers we have now added more evidence that supports our choice in the number of clusters (see **Figures 1C** and **Figure S7**).

We believe that having the possibility to use CTCF enrichment to delimit families of EPINs is more powerful than any other metric. Even if our split in 8 clusters is not optimal in any or some of the different metrics available like the silhouette coefficient, Davies-Bouldin Index, Calinski-Harabasz criterion or clustering-coefficient; our split is validated as it shows a consistent (down the hierarchy) and significant enrichment in a relevant biological feature.

Motivated by this comment and that of **Referee 1** (**point 4**), we made the decision to utilize a benign human prostate epithelial cell line (LHSAR) as our baseline for comparison with LNCaP cells. To ensure a comprehensive analysis, we performed HiChIP experimental data generation specifically for LHSAR cells, as there were no existing datasets available. Subsequently, we applied the PPI clustering procedure to further explore the functional relationships within the acquired data (**Figure 2**). In the case of LNCaP, the partitioning in 8 / 16 clusters resulted in the identification of 1 / 2 clusters with a significant enrichment in oncogenes. By contrast, in the control cell line LHSAR, a non-significant fraction of oncogenes was found only in 1 cluster out of 16 clusters, with enrichment in one type of GWAS signal (Catalog) and not in CTCF binding sites. These results lead us to conclude that PENGUIN, along with the integration of intermediate PPI networks, significantly enhances the identification of candidate PrCa-related SNPs.

2. Since it is widely known and reported that hierarchical clustering works poorly with the mixed data types (which have been used for the development of the tool) and missing datatypes (which is quite possible in the next-gen sequencing datasets), the authors should comment on the choice of using hierarchical clustering. Please make proper comparisons with other clustering methods, including k-means, NMF.

As mentioned in our previous answer, we use hierarchical clustering as it allows us to test for feature enrichment all the hierarchy of the proposed splits. We could apply this methodology on successive selections of K centroids but this would result in conflicts in the dendrogram representation which helped us in the exploratory analysis that we are presenting here.

On the specific point raised by the reviewer about the use of mixed datatype for the clustering, we would like to mention that our clustering is based on the list of edges that are present in each EPINs. There are therefore no missing datatypes (no NGS data involved) nor mixed datatypes. We use a single value representative of EPIN similarities by counting the presence/absence of equivalent edges (same protein-protein interaction). The inclusion of other datatypes (e.g. GWAS SNPs, or CTCF binding sites) is used on the clustering already performed to test for enrichment or depletion (fisher test). We made this point clear now in the Clustering EPIN section:

> We defined EPIN clusters by taking into account their edge content. Each edge consists of an individual pairwise PPI as defined previously. We collected the full universe of edges using all existent edges between all promoter EPINs (the union graph). Then we computed the distance between EPINs by counting the number edges shared over the total number of edges in our predefined universe of edges. Finally we performed clustering using this distance matrix from all possible combinations of EPIN pairs. The clustering was performed using Ward's linkage method. Each leaf in the obtained cluster represents a promoter EPIN.

3. It is very alarming that the authors have not shared the scripts/code during the review process. I recommend that the authors share the scripts so that anyone and everyone can go over them and understand the approach methodically.
We completely agree with the reviewer, The links to our github repositories were accidentally lost during the preparation of the manuscript. We have now added them back.

We completely agree with the reviewer, and we actually deleted these links by accident before submission. They are now back at the end of the manuscript:

> Source code of the related to the PENGUIN protocol is available at github: https://github.com/bsc-life/penguin_software
> Source code of the related to the PENGUIN web service is available at github: https://github.com/bsc-life/penguin_analytics

4. Moreover, the webserver https://penguin.life.bsc.es/ cannot reproduce the results reported in Figure S6. I ran it on Chrome, Firefox and Safari (on macbook), but it still gives a blank page after submitting the query on default values. I recommend that the authors keep the web server up and running so that it can be (extensively) tested by others too.

We are sorry for the inconvenience. The web server was down as a result of a power outage affecting the server hosting the corresponding Virtual Machine. We have restarted the VM accordingly.

5. One of the other drawbacks of the paper is the use of only one example to suggest the efficacy of the tool. The authors should it perform a similar analysis for other cancer or other disease types.

We thank the Reviewer for their remark. At the time of reviewing, the web-server was unfortunately down. We provide the entire set of all promoters in the web-server, and the user can plot any gene of choice directly from the web-server.

In any case, we provide additional examples in **Figure S10A,B.**

The choice to concentrate our efforts on applying our method exclusively to prostate cancer stems primarily from the pressing need for research in this field, as prostate cancer is the second most frequently diagnosed cancer in men. Additionally, it aligns with the expertise of our team and, significantly, the presence of exceptionally high quality data, enabling us to highlight the significance of multi-omics data acquisition in conducting comprehensive studies on cancer.

Nevertheless, we recognize the importance of demonstrating the applicability of PENGUIN in different scenarios. Thus, we embarked on conducting the following supplementary analyses: (1) we implemented the identical workflow utilized in LNCaP on LHSAR, a non-cancerous prostate epithelial cell line, and (2) we inferred the influence of varying the EPIN networks by using protein-protein interactions definitions from different cancers. This last analysis represents an alternative to repeating the whole analysis in another cancer type given that PPI networks represent the core information within our workflow and also given the existing unavailability of comprehensive multi-omics data for all the cancers considered.

We have now extended part of the analysis to another (non-cancer) cell line, LHSAR showing that the E-P networks are highly specific. We are aware that this only partially answer the point raised here by the Reviewer, but we are currently limited by the amount of available data (i.e. Hi-ChIP etc).

To support our main claim that the information in protein-protein networks allows us to identify disease-specific networks, which is the most important ingredient of our model (see **Figure S6**), we performed additional statistics on the PPIs of different cancer cells. In essence, using the Jaccard index to measure the overlap between PPI networks, we can show that the interactions are highly cell specific.

We added to the main text:

> Despite the high similarity in PPIs between LHSAR and LNCaP cells (Jaccard index of 0.85), their clustering based on H3K27Ac HiChIP data revealed distinct EPINs (**Figure 2B**). This finding highlights the sensitivity of our method in capturing subtle differences within EPINs. To further validate this, we conducted additional statistical analyses on PPIs across different cancer cell types. By examining the overlap between PPI networks, we discovered significant variations that were highly specific to each cell type (**Figure S6**). This observation not only reinforces the reliability of the differences found in LHSAR and LNCaP cells but also suggests that our results can be expected in other cellular contexts provided the required H3K27ac-HiChIP information, which is currently unavailable in most cases.

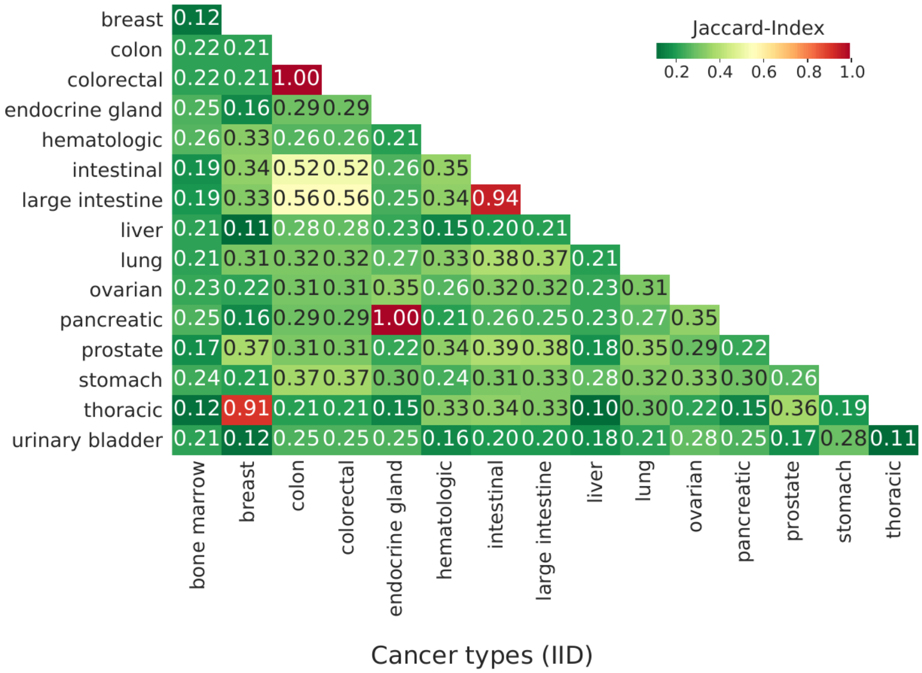

Figure S6. PPI networks comparison. Statistical analyses on PPIs across cancer cell types available at http://iid.ophid.utoronto.ca/. Using the Jaccard index we studied the overlap between PPI networks observing significant variations that were highly specific to each cell type. The results show that the PPIs used in PENGUIN vary significantly depending on the cell types of interest.

This analysis shows the low levels of overlap between cancer types in the IID database (**Methods**). Together with our recent analysis in **Figure S6**, we can expect that applying PENGUIN to other cancer type will result in highly specific results.

6. The authors suggest that the functional relationship between most of the SNPs and PrCa is unknown and that PENGUIN (with the overlap analysis with PAINTOR results and other GWAS catalog datasets) can find the potentially causal SNPs. Can authors please validate (using CRISPRi as suggested by authors) these top candidates reported in S10 that has not been reported before? I would like to know whether these hits are true or false positives suggesting that the tool is useful.

This is a great point and we are very grateful for the suggestion. Fortunately, such validation is possible through reported pooled genome-wide CRISPR/Cas9 knockout and RNAi screens conducted in prostate cancer LNCaP cells, available in the DepMap database (https://depmap.org/, DepMap ID: ACH-000977).

We added this to the main text:

> To establish the biological significance of the identified SNPs, we leveraged data from previous pooled genome-wide CRISPR/Cas9 knockout and RNAi screens conducted in prostate cancer LNCaP cells, available in the DepMap database (https://depmap.org/, DepMap ID: ACH-000977). These screens provide essentiality scores, which quantify the relevance of specific gene networks to the proliferation of LNCaP cells. In our analysis, we retrieved essentiality scores for genes in prostate tissue from DepMap and compared three distinct gene sets: (1) the genes (EPIN promoters) prioritized in **Table S10**, (2) all genes (EPIN promoters) included in our analysis, and

(3) all genes available in the DepMap database. Remarkably, we observed significant differences in the essentiality scores (Z-scores) among these sets, with lower Z-scores indicating a higher degree of gene essentiality (**Figure 4A**). This analysis aligns with the RNAi findings, demonstrating a significant decrease in essential scores for genes containing the SNPs listed in **Table S10** (**Figure 4B**). Furthermore, the GSEA analysis unveiled a noteworthy enrichment (p-value = 0.0017) for these EPIN promoters that harbor intermediate nodes with SNPs at their genomic location (as indicated in the supplementary **Table S10**) (**Figure 4C**). Among the top essential genes, the CRISPR/Cas9 and RNAi screens prioritize the following ones : GATA2-AS1, CASZ1, MYC, KRT8, GTPBP4-AS1, MFN2, CTBP2, and ID2.

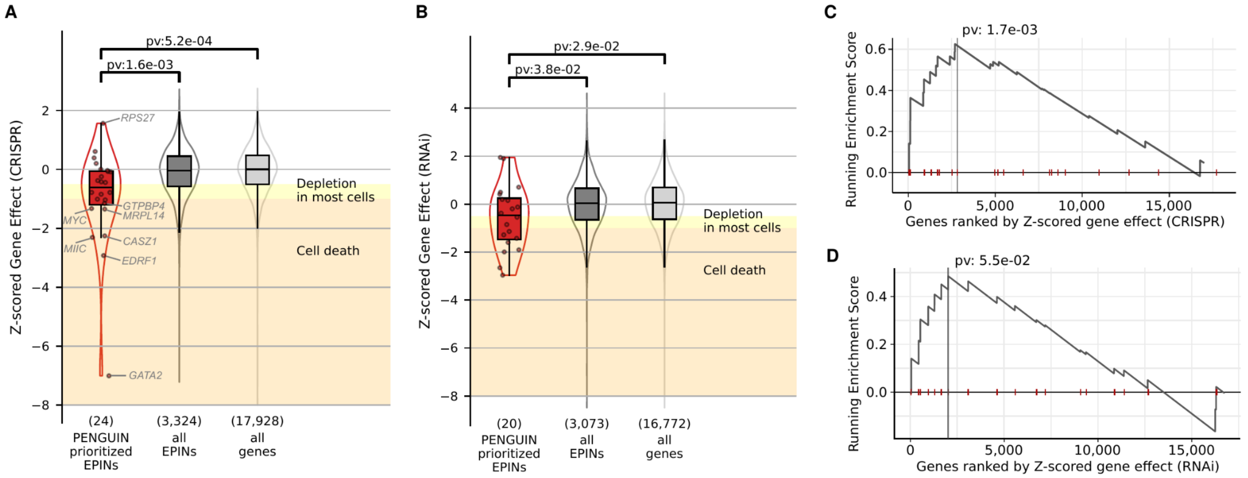

Figure 4. Validation of SNPs prioritized by PENGUIN. CRISPR/Cas9 knockout and RNAi screens provide Z- scores to quantify the relevance of a specific gene network to proliferation of LNCaP cells. (**A**) CRISPR/Cas9 knockout analysis indicates that intermediate SNPs prioritized by PENGUIN occur in genes essential for LNCaP (significance calculated with Mann-Whitney test). Genes with the strongest effect are displayed. (**B**) RNAi analysis shows milder but significantly consistent results with CRISPR/Cas9 knockout. (**C**) Gene Set Enrichment Analysis (GSEA) indicates that SNPs prioritized by PENGUIN occur in the most essential genes identified by CRISPR/Cas9 knockouts. (**D**) GSEA indicates that SNPs prioritized by PENGUIN occur in the most essential ones based on the RNAi screen. for C and D, the statistical significance of the enrichment of a gene set within the ranked gene list is reported.

6. That comes to the following query. Under the method section, the authors included the PrCA SNPs above the genome-wide p-value threshold. It is not a reasonable practice in GWAS analysis as this increases the false positive rate. Can the authors please include the number of SNPs that passed the threshold vs not, and how will this affect the results reported in table S10?

This is a misunderstanding that we have now clarified the text in different points.

PrCA SNPs are not identified with a genome-wide p-value threshold, rather, the PrCa SNPs are defined using a Bayesian fine-mapping tool which identifies 95% credible set as described under Methods section **PrCa SNPs** and reported in the previous Giambartolomei and Seo et al., AJHG 2021 paper. This paper had previously reported 5,412 distinct SNPs (rsid) identified with fine-mapping. In all of our analyses where we report SNPs, we also make sure to list the full information on the GWAS p-value (column “PVAL_PrCa”, column C and column G in n Tables S10 and Table S11, respectively). In the Methods we report now:

PrCa SNPs

To explore enrichment of SNPs associated to PrCa across the identified clusters, and to identify the SNP paths, we used the previously reported 95% credible set[11] from fine-mapping 137 previously-associated PrCa regions using a Bayesian statistical method PAINTOR [59] employing the largest PrCa genome-wide association studies (GWAS) (N = 79,148 cases and 61,106 controls)[60]. This set was composed of 5,412 distinct SNPs (rsid). We will refer to these as PrCa SNPs. Note that this set also includes SNPs that do not reach genome-wide-filters of p-value significance. We illustrate the location of the associated PrCa regions and number of PrCa SNPs in **Figure S11**. We did not find a significant correlation between the number of PrCa SNPs in the regions and the number of PrCa SNPs we prioritized in this work (Pearson r=0.2, p-value=0.06 and Pearson r=0.1, p-value=0.3 for **Tables S10** and **S11**, respectively). We mapped the SNP location to prioritized enhancer regions anchor locations with a window of 10 kb. 518 out of 5,412 overlap our prioritized enhancer regions; 18 of them overlap our promoter regions. In total 218 prioritized enhancers and 14 promoters overlap a PrCa SNP.

And

SNP paths (PrCa SNPs in enhancer binding motifs)
A path in a network is a sequence of edges joining a sequence of nodes. We detected PrCa SNPs located in the DNA binding motifs in the enhancers, and identified the corresponding SNP paths (linked edges and nodes) for each EPIN promoter. For SNP paths analyses and the web-browser, we used all PrCa SNPs in the 95% credible set. There were 36 PrCa SNPs falling in enhancer binding motifs across clusters 3, 4, 5, 6, 7, 8. To report the most interesting cases in the Tables and Results, we used the subset of those passing genome-wide significance of p-value for PrCa association < 5e-8. There were 15 PrCa SNPs falling in enhancer binding motifs across clusters 3, 5, 6, 7, 8.

Table S10 reports 36 PrCa SNPs falling within 60 enhancer DNA binding motifs across clusters 3, 4, 5, 6, 7, 8. This links 34 unique promoters with PrCa SNPs in enhancer binding motifs. To report the most interesting cases in the Tables and Results, we used the subset of those passing genome-wide significance of p-value for PrCa association < 5e-8. There were 15 PrCa SNPs falling in enhancer binding motifs across clusters 3, 5, 6, 7, 8.

7. Can the authors please compare their results with the baseline and other tools, such as tools developed by Ratnakumar et al. and Dey et al. and focus the analyses around the EPIN regions? For baseline analysis, the authors can use the same datasets used to develop PENGUIN and do an overlap analysis suggesting that the mere intersection of datasets will have little power compared to a robust statistical analysis.

We thank the Reviewer for making these great points.

We now produced and include the following analyses in the manuscript:

(1) We have now added comparisons to baseline analyses (section "**Baseline comparisons and assessment of PENGUIN specificity")** as suggested by the Reviewer. We compare the mere intersection of the different datasets (E-P regions and PPI), with our EPIN approach. We compare the mere intersection between information from HiCHIP and from PPI., illustrating that a mere

intersection of datasets is performing worse with respect to enrichments of PrCa related annotations**.**

(2) We have clarified that we utilize PrCa annotations such as previously-associated PrCa SNPs and PrCa oncogenes to validate the link between PrCa and EPIN regions.

(3) Following the remark of this Reviewer and that of Reviewer 1, we have introduced a baseline comparison with another cell line (**see response to Reviewer 1 Question 4**). We compared the results in this benign prostate epithelial cell line (LHSAR) with our results in the LNCaP cell line. To ensure a comprehensive analysis, we performed HiChIP and H3K27ac-ChIP-Seq experimental data generation specifically for LHSAR cells, as there were no existing datasets available. Our EPIN analysis in LHSAR resulted as expected. LHSAR EPIN clusters were found to be enriched in CTCF, which can be seen as a positive control of our ability to detect biologically relevant signals. While the absence of any cluster enrichment in GWAS or specific oncogenes represents a validation of a negative control (**Figure S7**).

(4) We observed how PPI networks, and especially intermediate nodes, increase our power to detect PrCA-related genes. We repeated PENGUIN clustering, based solely on the list of enhancer IDs (names based on their genomic coordinates) in each EPIN (see **response to Reviewer 3 Question 3**). We found that the use of intermediate PPI networks increases the number of oncogenes identified. Thus, the information conveyed by the PPI network is relevant for EPIN classification and to their association with phenotype.

The approach from Ratnakumar et al., links GWAS Prostate cancer SNPs to a PPI gene by identifying genes that exhibit an excess of protein-protein interactions among GWAS hits in the networks. The authors' methodology is not specific for E-P mechanisms as PENGUIN. However we identified genes in common across PENGUIN and Table S2 of Ratnakumar et al. (after selecting for Europeans, genes considered in common, and GWAS Prostate cancer SNPs). 2 genes out of 20 from our **Table S10** ("SEC11C" "CTBP2") and 5 genes out of 20 from our **Table S11** ("CDKN1B" "BCL2" "CTBP2" "CHD3" "KDM2A").

Dey et al. (Cell Genom 2, 2022) propose SNP-to-gene linking strategies and devise the S2G score, which incorporates ten different functional approaches such as eQTLs, enhancer-gene links (including the ABC score from Fulco et al.), and PPI networks involving enhancer-related genes. On the other hand, PENGUIN links PrCa SNPs to genes within enhancer-promoter regions using the PPI network. Notably, in the Dey at al. analyses, enhancer-gene-linking strategies, including the ABC score, were found to be highly informative for the analyzed traits. Importantly, the integration of PPI networks with enhancer-promoter links exhibited substantial enrichment for disease heritability, aligning with our findings in prostate cancer. The authors assess a small set of phenotypes associated with autoimmune disease and blood traits, impeding a direct comparison of our prioritized PrCa SNPs with the Dey et al. analyses. On the other hand the ABC paper from Fulco et al. reports links between enhancer elements and promoters. Although this is out of scope for this paper, we have checked overlaps of the genes we have reported with the LNCaP-specific ABC score (**as mentioned in the response to Reviewer 3, Question 6**). Out of the common genes considered in ABC and PENGUIN (N genes = 3,619), 25 out of 29 genes identified in PENGUIN in Table S10 also have a high ABC score, and 9 out of 10 intermediate genes identified by PENGUIN in Table S11 also have a high ABC score. This points to a high concordance between ABC and enhancer-promoters from HiChIP, also as previously reported in Giambartolomei and Seo et al.

We believe the analyses listed above demonstrate that PENGUIN, and particularly the inclusion of intermediate PPIs, significantly aids the identification of factors related to PrCa.

8. Another recommendation would be to use more examples like Myc in the main figure.

We have indeed added new examples that can now be found in the main text and also in **Figure. S10A,B.**

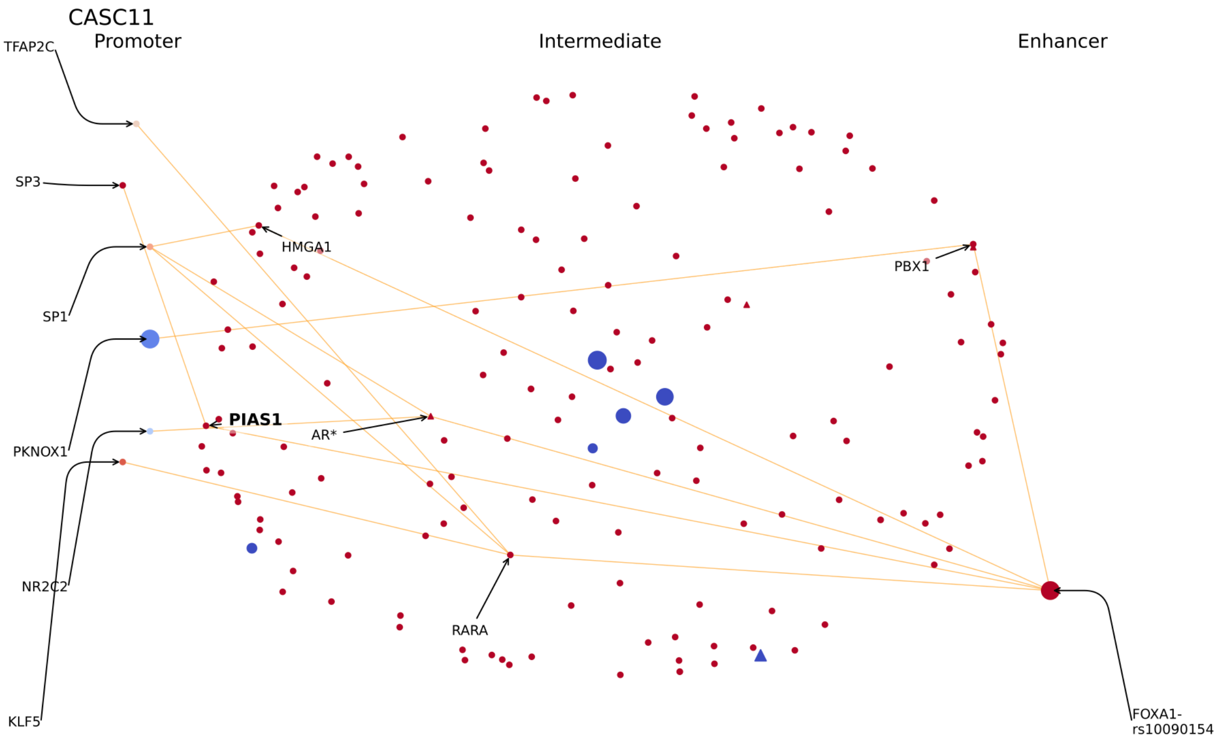

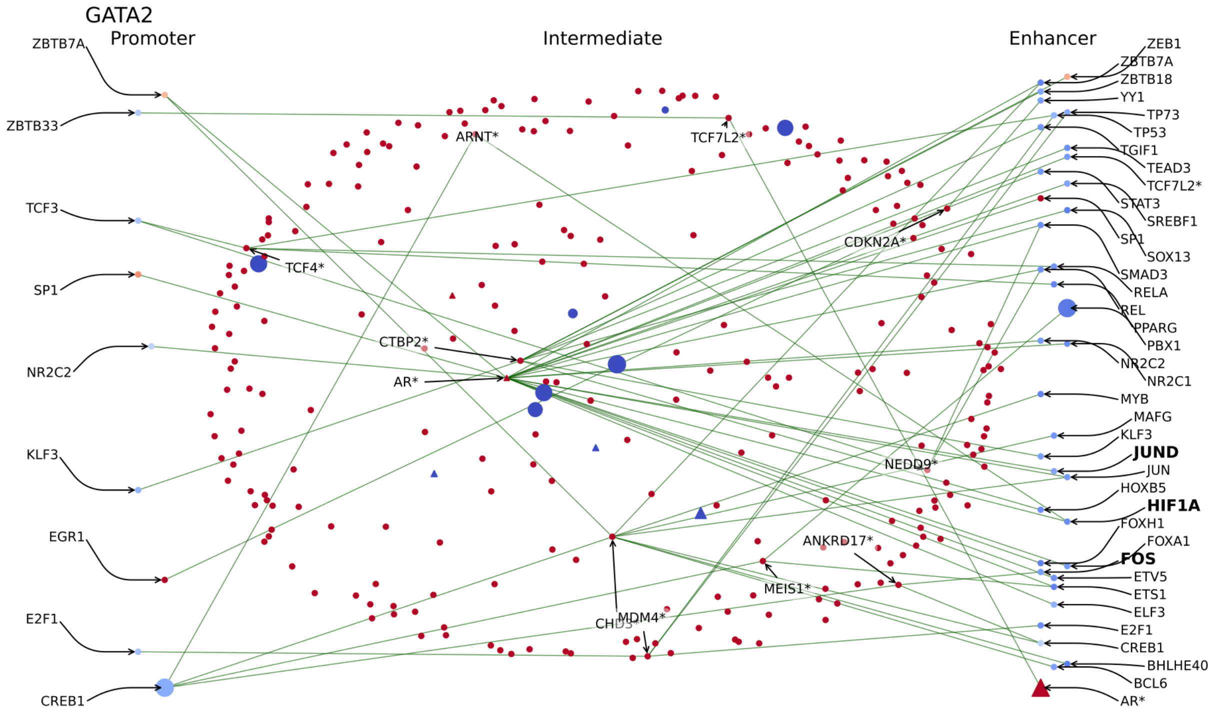

We report two additional examples, the EPINs for the promoters of CASC11 (**Figure S10A**) and GATA2 (**Figure S10B**). Yellow lines represent edges of the EPIN subnetwork starting from DNB bound to en enhancer anchor and whose binding site overlaps PrCa-associated SNPs (FOXA1 - rs10090154), while green lines represent the EPIN subnetwork with edges passing through nodes with PrCa-associated SNPs overlapping their genomic location.

9. This comment is regarding the RNA-seq analysis performed. The authors of Tophat/Cufflinks have themselves suggested avoiding the use of Cufflinks for the quantification of gene expression. It is because FPKM (or RPKM for paired-end datasets) requires all the samples to have the same amount of mRNA/cell across all the samples, which in practice, does not hold. For quantification use RSEM, Kallisto (for transcript-level quantifications) or count file output from STAR are generally recommended. Both will output. Next, it is not recommended to use FPKM or RPKM units for DE analysis using DESeq2. If the author wants to perform the DE analysis using DESeq2, they should use the counts file.

We agree with the Reviewer's general comments on the use of FPKM or RPKM for DESeq2 differential expression analysis. However here we just wanted to exclude from our dataset genes that we know were not expressed (not significantly less). Under this perspective, not considering gene length would not have been fair, as by chance , the probability of registering reads from long silent genes is much higher than from short silent genes. Our Supplementary figure 4C clearly shows that at our level of confidence very few genes would be even affected by the choice of the metric (direct counts or FPKM), we are basically removing genes with no RNA-seq counts.
In order to clarify this point we added this sentence in the corresponding section of methods ("Gene expression data"):

> Depending on the dataset, this expression lower-bound may be modified in different use cases, for instance based on specific insights or based on a differential analysis between conditions. In this

work, we used FPKM instead of more direct measures as we set our threshold very low and did not want to enrich our dataset with very long, virtually unexpressed, transcripts.

We have added Table descriptions to the Supplementary Materials.

REVIEWER COMMENTS

Reviewer #1 (Remarks to the Author):

This reviewed submission is fantastic and they authors have done a great job in responding to the concerns and queries raised.

The only additional suggestion I had was the response to my 2nd query in the original peer review, discussing the promoters used in the manuscript.

Perhaps this misinterpreted my suggestion. I wasn't really worried about the activity of promoters, rather the size of the promoter taken (i.e. the promoter is not always 500bp from TSS - it could be 5kb from the TSS). The FANTOM5 CAGE data that I suggested would give you the exact size range of the promoter to use, rather than using an arbitrary size

Reviewer #3 (Remarks to the Author):

The authors have adequately addressed my comments. I do not have any more comments and recommend accepting the manuscript.

Reviewer #4 (Remarks to the Author):

I would like to thank the authors (Armaos et al.) for answering the questions. However, there are major concerns with the robustness of the model or method, the use of correct metrics for the quantification of gene expression, and comparisons with other published methods. They are as follows:

1. To my 1st comment, the authors argue that the significant enrichment for CTCF in EPINs for cluster 8 is more powerful or meaningful than any other metric, including silhouette coefficient, Davies-Bouldin Index, Calinski-Harabasz criterion or clustering-coefficient. This leads to the following questions:

a) It needs to be clarified why CTCF enrichment would be that powerful than any properly defined statistical metric.

b) It is possible that the signals or observation they found for cluster 8 is just by "chance" and may not apply to other datasets. If the research community uses the tool, how will the users come up with the correct number of clusters or k? Similar to p-value hacking or cherry picking, it seems an exploratory signal hacking tool (without sound methodology or statistical robustness) where the results may vary as the k will change and the fitting of the best k (without the use of any statistical principles) is left to the disposal of the users. It will hence lead to many false-positives observations/conclusions/answers.

c) Since the authors are experts in Prostate cancer, it is possible that there is an inherent prior knowledge and bias that leads them to choose k = 8. What about the researchers who would like to use it on an entirely new dataset without any prior knowledge? Will this tool be helpful in that case? The efficacy of the tool is in question.

2. To my 7th comment, the authors need to perform a proper and in-depth comparison with the tools such as ABC scores, Dey et al. Even if these tools link SNP to the genes, the authors can use SNP within enhancers to connect it to the promoters of the genes. Moreover, the calculation of ABC scores suggested in their rebuttal is not shown in the Supp table S10 or S11.

2. To my 9th comment, it is still not clear how Supp Figure 4C (comparison of the number of edges v.s. the total clusters) answers the question using the choice of correct RNA-seq units

(FPKM/TPM/normalized counts). How the gene expression is normalized is still an issue. The basic assumption of DESeq2 is that the gene expression dataset follows a negative binomial distribution, whereas FPKM normalized datasets do not.

Reviewer #1 (Remarks to the Author):

This reviewed submission is fantastic and they authors have done a great job in responding to the concerns and queries raised.

Thank you so much for the 'fantastic'! We are so excited for the new results obtained and very much looking forward to having them published.

The only additional suggestion I had was the response to my 2nd query in the original peer review, discussing the promoters used in the manuscript.
X
Perhaps this misinterpreted my suggestion. ***I wasn't really worried about the activity of promoters, rather the size of the promoter taken*** *(i.e. the promoter is not always 500bp from TSS - it could be 5kb from the TSS). The FANTOM5 CAGE data that I suggested would give you the exact size range of the promoter to use, rather than using an arbitrary size*

We thank the reviewer for the great positive feedback.

Regarding the additional suggestion, the reviewer points out that the length of the promoter could be different from what we have used in the PENGUIN analyses, which is +/- 500bp from the TSS. The reviewer mentions promoter 5kb from their TSS, and we believe that these cases are relatively rare, the general convention is to consider promoters to be between 100 and 1,000 nucleotides long (1) . The reviewer mentions the work leading to the FANTOM5 CAGE database, however, these same authors used a 1kb window, +/- 500bp from the TSS, to define the promoter regions and to scan for TFBS (2) following the same protocol as ours. We have added this reference to our method section to support our choice:

> We categorized interactions by overlapping anchors with transcription start sites (TSS) and enhancers identified by H3K27ac ChIP-seq as previously described [11]. Briefly, we first extended anchors by 5 kb on either side; we defined promoter regions around the TSS (+/- 500 bases) [52] using RefSeq hg19 (see **Data Availability**); we defined enhancer regions using regions from H3K27ac ChipSeq in the same cell. Specifically, these were 49,638 and 53,561 enhancer

Finally we agree with the reviewer that our definition of promoter can be improved. Adding cell specificity with CAGE data on our cell-line to infer the exact TSS positions. Unfortunately no CAGE data is currently available for our LHSAR or LNCaP cell lines (not in FANTOM 5 nor in FANTOM 6).

Reviewer #3 (Remarks to the Author):

The authors have adequately addressed my comments. I do not have any more comments and recommend accepting the manuscript.

We thank the reviewer for the positive recommendation.

Reviewer #4 (Remarks to the Author):

I would like to thank the authors (Armaos et al.) for answering the questions. However, there are major concerns with the robustness of the model or method, the use of correct metrics for the quantification of gene expression, and comparisons with other published methods. They are as follows:

1. To my 1st comment, the authors argue that the significant enrichment for CTCF in EPINs for cluster 8 is more powerful or meaningful than any other metric, including silhouette coefficient, Davies-Bouldin Index, Calinski-Harabasz criterion or clustering-coefficient. This leads to the following questions:

a) It needs to be clarified why CTCF enrichment would be that powerful than any properly defined statistical metric.

Thank you for this remark. We are happy to provide more details regarding the significance of CTCF enrichment as a metric in our study. To clarify, our analysis within the EPINs framework revealed that GWAS and CTCF signals, coming from two independent datasets and not used to build the clusters, are both uniquely enriched in what we identify as 'cluster 8'. CTCF, and GWAS, are not selection criteria, they are identification criteria. We further corroborated the importance of this co-enrichment by comparing LNCAP with a control cell line, LSHAR. This control cell line shows an enrichment in CTCF signal in clusters without GWAS enrichment (**Figure 2**). The robustness of our approach is further supported by the fact that the identified SNPs play a crucial role in LNCAP cell proliferation, which was validated using CRISPR technology (**Figure 4**).

In further detail, CTCF serves as a vital structural protein with conserved functions across the animal kingdom. Its inclusion in our study not only aligns with current literature but also provides biological interpretability to chromatin structures. The utilization of CTCF as a marker is therefore highly justified, given its central role in controlling chromatin conformation. This is substantiated by numerous studies that have employed CTCF to validate and characterize a variety of chromatin structures, such as topologically associated domains (TADs) (3), lamina-associated domains (LADs) (4), chromatin loops (5), transcriptional condensates (in instances where CTCF is absent) (6), and conserved chromatin interacting modules (7).

Regarding the query about whether the use of CTCF is more powerful or meaningful, than the use of other metrics, the reviewer proposes the silhouette coefficient, Davies-Bouldin Index, Calinski-Harabasz criterion and the clustering-coefficient. These metrics are few of many available cluster validity metrics (CVIs) [here a comparison of 30 different CVIs (8)]. CVIs have two major problems: 1- they are not robust to noise or cluster overlap, and even if, in ideal cases, CVIs tend to give similar results, it is recommended to use several CVIs to increase robustness. And with different predictions on the best partitioning, the final decision is entirely left up to the user's expertise. Best partitioning solutions are not evolving in a linear space and one cannot average on these predictions. 2- CVIs use the same data as input to the clustering algorithm. Using external validation data should always be preferred, this is how CVIs are benchmarked, and this is what CTCF enrichment represents in the context of this work.

In order to exemplify these problems with our data, we have run different CVIs on our main LnCAP cluster (shown in **Figure 2**). We have used the proposed silhouette coefficient, Davies-Bouldin Index and Calinski-Harabasz criterion, but not the clustering-coefficient (we only found implementations to be applied on graphs (9). We also added a more recent metric called GAP (10) and its derivative (11).

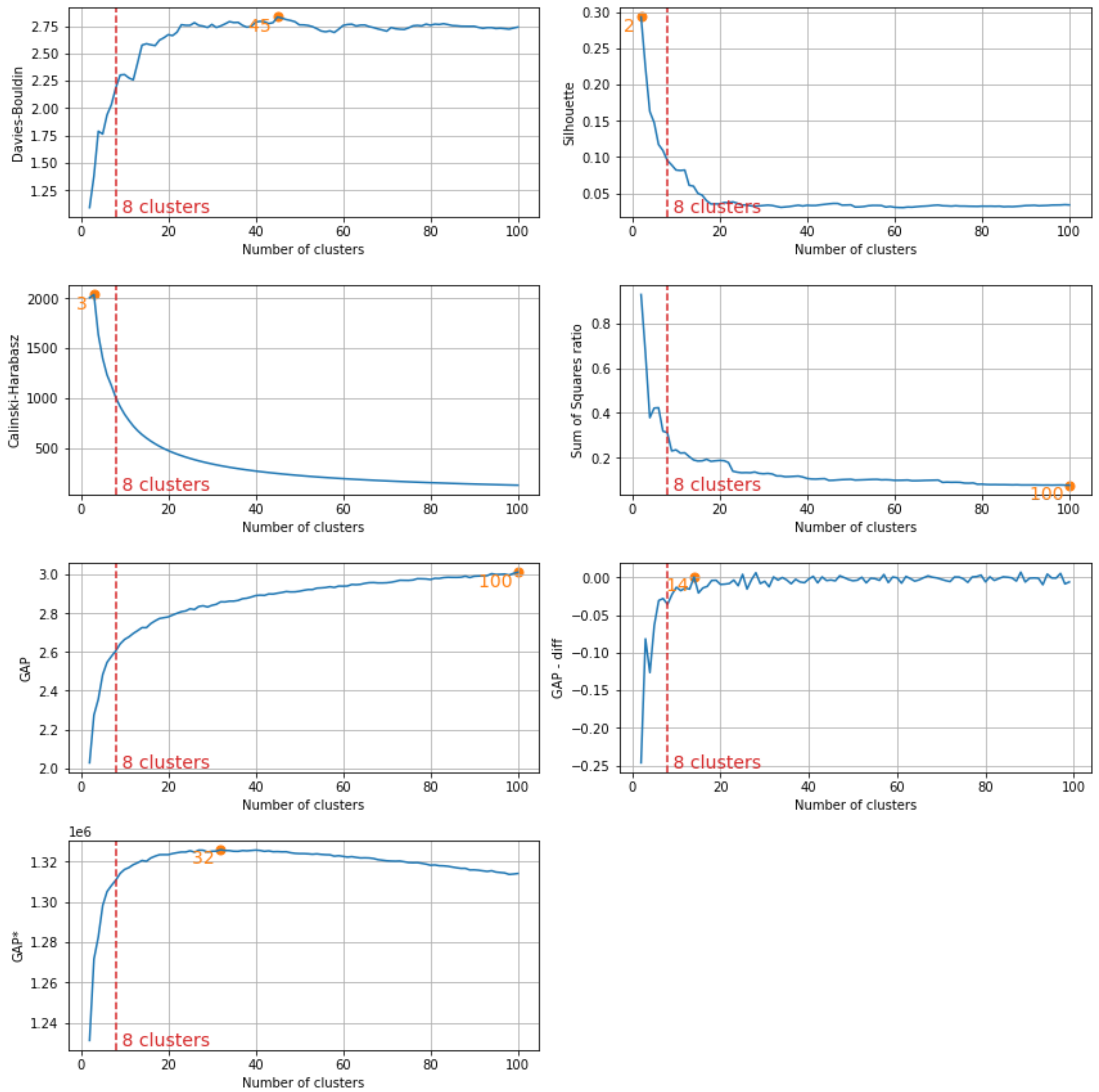

Cluster validity indexes: In each panel we represent the CVIs calculated on the LNCAPdataset for a number of clusters between 2 and 100. In red the 8 clusters that we defined manually based on CTCF enrichment, in orange we show the best number of clusters for each index. The indexes we use are: Davies-Bouldin (12), Silhouette (13) , Calinski-Harabasz (14), a simple sum of squared differences within cluster divided by total, GAP (10), GAP-diff (first positive difference between GAP value at k and GAP value at k+1 considering the expected error) and GAP* implemented to correct the overestimation of the number of clusters (11).

According to these results, we found that depending on the CVI used, the best clustering occurred for 2, 3, 14, 32, 45, and 100 partitions. **The 8 clusters that we defined based on CTCF and GWAS enrichment stand in the elbow of the distributions, away from the less stable parts of the clustering.** According to these results we can argue that 8 clusters is a relatively fair choice.

However, our partitioning protocol based on CTCF and GWAS enrichment is more biologically meaningful and robust (two completely independent experimental dataset showing a statistically significant enrichment in the

same partition). In order to explain better the reasoning behind our choice of 8 clusters we have now added a more detailed explanation in the manuscript in the "*Characterization of PrCa clusters identified by PENGUIN*" section of the ***Results***.

b) It is possible that the signals or observation they found for cluster 8 is just by "chance" and may not apply to other datasets. If the research community uses the tool, how will the users come up with the correct number of clusters or k? Similar to p-value hacking or cherry picking, it seems an exploratory signal hacking tool (without sound methodology or statistical robustness) where the results may vary as the k will change and the fitting of the best k (without the use of any statistical principles) is left to the disposal of the users. It will hence lead to many false-positives observations/conclusions/answers.

We appreciate the reviewer's question about the potential for "chance" findings or false positives arising from our methodology. In the original submission of our manuscript, **Figure 2** already highlighted the decline of both CTCF and GWAS enrichment as we move down the hierarchy of cluster 8. To make this point more explicit, we have elaborated on these enrichments in our previous response. We have demonstrated multimodal enrichment in oncogenes, prostate-related SNPs, and CTCF across 4, 8, and 16 clusters, always in a cluster that is either a parent or descendent node of our focal GWAS+ cluster 8 (as illustrated in **Figure 2C**). We have also included **Table S3B** to detail all our statistical tests with 4, 8, and 16 clusters for both LNCaP and LHSAR.

It is important to note that users of PENGUIN would similarly be looking for associations between hierarchical clustering of EPINs and a feature of interest. We stress that we found significant associations with prostate SNPs and oncogenes in the LNCaP dataset but observed no such trends in the LHSAR dataset. Therefore, our methodology is not reliant on pre-defining a specific clustering level to identify meaningful associations. The grouping or partitioning of EPINs is utilized merely to characterize those EPINs most strongly associated with prostate cancer, not to establish a one-size-fits-all clustering approach.

We would like to underscore that, in addition to the findings originally presented in the initial version of our manuscript, our previous rebuttal also provided evidence supporting the definition of cluster 8. Specifically, we demonstrated the enrichment of essential genes within this cluster, as documented in **Table S10** and **Figure 4.** It is noteworthy that this validation of our partition definition emerged from one of the concerns raised by the reviewer. We appreciate the thorough evaluation of our work and the opportunity to clarify the robustness of our findings.

Finally, we understand the reviewer's concern about statistical robustness, but none of the metrics the reviewer proposes are helpful in this sense. These are just guiding tools to find most differentiated clusters, none of these tools can be associated with statistical power or significance. Hence the variety of results associated with their use. In general terms to demonstrate the robustness and reproducibility of a clustering we would recommend, for example, to use methods based on bootstrapping as *pvclust* (15) or on Monte Carlo procedure as *SigClust2* (16). Note that this would not answer the reviewer's question about the best partitioning, but show how robust a given partition is. We run *SigClust2* with the following parameters: *n_min=150, alpha=0.05.* These two parameters are meant to reduce computational demand and relate to the minimum cluster size to test (*n_min*) and the *alpha* to limit the number of tests performed by the family-wise error rate (FWER) to only those already significant.

We found that our clustering is very robust at all the levels tested, and this includes the *GWAS+* cluster and its 7 sister clusters.

Cluster	Normalized p-value based on 2-means cluster index
1	0.000000e+00
2	0.000000e+00
3	7.804422e-69
4	6.257220e-272
5	6.760082e-77
6	1.025465e-70
7	1.850507e-106
8	1.597306e-77

We have added a sentence in the corresponding method section (**Clustering EPINs**) to summarize this result:

> method. Each leaf in the obtained cluster represents a promoter EPIN. In order to assess the robustness of this result we applied the *SigClust2* MonteCarlo procedure [58] on our clustering with the following parameters: *n_min=150, alpha=0.05*. We found that the first eight partitions of our hierarchical clustering were very robust with the following Normalized p-values (2-means cluster index): 0, 0, 8e-69, 6e-272, 7e-77, 1e-70, 2e-106 and 2e-77 for clusters 1 to 8 respectively (according to the labeling in **Figure 2**).

Note that we have not been able to use the *pvclust* algorithm on our data because of the size of the dataset, but we know by experience that *SigClust2* is more conservative in terms of calling a cluster significant.

We hope this clarifies any concerns regarding the statistical robustness and applicability of our method.

c) Since the authors are experts in Prostate cancer, it is possible that there is an inherent prior knowledge and bias that leads them to choose k = 8. What about the researchers who would like to use it on an entirely new dataset without any prior knowledge? Will this tool be helpful in that case? The efficacy of the tool is in question.

We appreciate the reviewer's question regarding the potential bias in our choice of k=8 due to our expertise in prostate cancer. We would like to emphasize that the PENGUIN method itself is not influenced by any disease-specific or prostate cancer-specific knowledge. The crux of the PENGUIN approach is to cluster EPIN structures. After completing this clustering, researchers can then validate their hypotheses by looking for significant enrichments or correlations within these clusters.

In the latest revision of our manuscript, we provided a counterexample using the LHSAR dataset, where no significant correlation between EPIN structure and any prostate cancer specific phenotype was observed. Researchers utilizing PENGUIN can similarly probe their EPIN structures and look for correlations with features most relevant to their study. In such cases, we recommend a deeper investigation of the identified most relevant EPINs to explore their components and the observed associations further. Importantly, no preset value of k is necessary for this process.

Furthermore, if researchers are interested in broader-scale associations, they can identify clusters enriched in a particular feature and then characterize those clusters in terms of common PPI pathways, presence of SNPs, observed differential expression etc., regardless of what k is chosen. The choice of k merely facilitates grouping structurally similar EPINs that relate to a given feature.

We hope this clarifies any concerns regarding the tool's efficacy and its adaptability to various research needs. This version aims to clarify that the tool is versatile and can be used in various research settings, not just those focused on prostate cancer.

2. To my 7th comment, the authors need to perform a proper and in-depth comparison with the tools such as ABC scores, Dey et al. Even if these tools link SNP to the genes, the authors can use SNP within enhancers to connect it to the promoters of the genes. Moreover, the calculation of ABC scores suggested in their rebuttal is not shown in the Supp table S10 or S11.

We appreciate the reviewer's continued engagement with our work and the suggestion to perform in-depth comparisons with previously published tools. Regarding the 7th comment, while we understand the importance of comparative analyses, we maintain that a direct comparison between Activity-By-Contact (ABC) and HiChIP experiment to link enhancers to promoters is beyond the scope of this paper, but it would be interesting in future analyses to apply PENGUIN with different input data including other methods linking enhancers to promoters. PENGUIN relies on experimentally validated enhancer-promoter links derived from H3K27ac-HiChIP, which is cell-specific and generally considered more reliable than computational predictions. ABC, on the other hand, employs a computational method to infer enhancer-promoter interactions.

However, to accommodate the suggestion, we have now included an analysis that examines overlaps between the SNPs listed in **Table S10** and enhancers connected to promoters as per ABC data and add this to Methods and Discussion. We also calculated the corresponding scores for those enhancer-promoter links that are concordant with ABC.

Turning to the query about comparisons with Dey et al., we recognize that their work provides valuable SNP-to-gene linking strategies that include ABC scoring and is focused on a narrow set of phenotypes. In a related paper by Gazal et al., with Dey as a co-author, a more extensive SNP-to-gene mapping approach is used. We have addressed this in Discussion. To address this point, we have compared our cell-specific results in **Table S10** with those produced by the cS2G strategy outlined in the Gazal et al. paper.

Specifically, we have added sections copied below in **Results**, the section in **Methods** ("Annotations **of PENGUIN prioritized signals using ABC and cS2G scores)'**, the section copied below in **Discussion**, and new **Table S10**.

In Results

In order to assess the usability of PENGUIN in the absence of HiChIP data we compared our results with Activity-By-Contact (ABC) scores[34]. We overlapped the fine-mapped GWAS SNPs with the enhancers reported in ABC. 17 out of the 36 SNP-gene links reported in **Table S10** overlap an enhancer linked to the same promoter in the ABC score. Three SNP-gene links have high support from the ABC model (ABC score ≥ 0.022), rs55958994-KRT8/KRT18 and rs143499963-DLL1/FAM120B and rs10818488-C5, while rs10090154-MYC/CASC11 have low support (0.0284). Overall these results show a partial overlap when using HiChIP experiments in the PENGUIN approach as opposed to the computational predictions of enhancer-promoter functional contacts to explain the association between SNPs and disease.

Additionally, we used the SNP-Gene-Disease linking strategy cS2G from Gazal and colleagues [35] , to identified 8 SNP-Gene-Disease links with 4 genes (*CTBP2, MYC, ID2, KRT18*) that were also considered in **Table S10**, which represent the links with strong support from multiple epigenetic information across different cell lines.

In Methods - see '**Annotations of PENGUIN prioritized signals using ABC and cS2G scores'**

In Discussion

Previous computational approaches have linked enhancers to gene[34]. In this work, PENGUIN uses information on enhancer-promoter interactions using the HiCHIP experiment. There are other ways that this link could be identified. For example, Activity-By-Contact (ABC), a computational prediction method linking an enhancer region to its supposed target gene[34], could be used instead of HiCHIP observations. We leave this for future explorations. As we note from Results, as well as for HiChIP interactions, PENGUIN is able to complete and extend the information given by solely using enhancer-promoter interactions. In a recent study, Dey et al. [46] demonstrated the benefits of employing strategies that capture both distal and proximal gene regulation in prioritizing autoimmune-disease related genes. Similarly to our findings, the authors found that incorporating enhancer-gene links (including the ABC score from Fulco et al. [34]), and PPI networks are important to link SNPs-to-gene [46].

Other previous computation approaches had the goal of linking SNP-Gene-Disease (cS2G from Gazal and colleagues [35]) by combining information across different cell lines. Additionally, previous studies have incorporated PPI networks with GWAS hits to enhance their analysis [47].

We have added three columns to **Table S10**:

ABC_Score: Activity-By-Contact (ABC) score linking an enhancer region to its supposed target gene.
cS2G_SNP: SNP reported to be linked to the PENGUIN gene by the cS2G SNP-Gene-Disease linking strategies from Gazal and colleagues.
R2_CEU_PENGUIN_SNP_cS2G_SNP: Linkage disequilibrium (measure by R2) between the SNP reported in PENGUIN in the SNP column and the cS2G SNP.

Additionally, the Figure below illustrates that the ABC score for the E-P links reported in ABC that were used in PENGUIN had a higher ABC score (mean ABC score for matched 0.0155 compared to all 0.0038).
For the reasons explained above, we do not think it is necessary to insist on this point any further and have not included this Figure in the current version of the manuscript, but in case the Reviewer thinks this is an important addition we would be happy to do so.

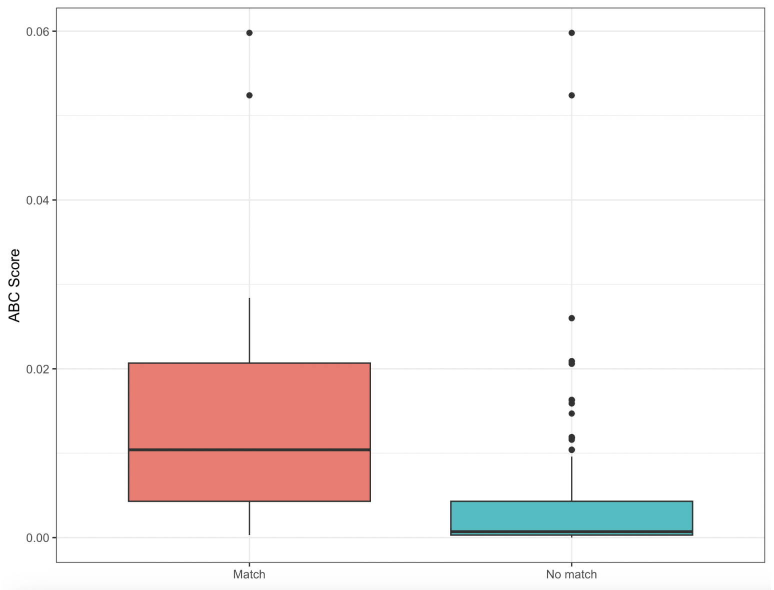

ABC score analysis. Comparison of ABC score in gene-enhancers links that are reported in PENGUIN versus those that are not, using ABC file in LNCaP cell (LNCAP.AllPredictions.txt). Using genes and enhancers considered in both PENGUIN and ABC, of the 199,742 enhancer-promoter links in ABC, 9,567 match the exact enhancer-promoter link in PENGUIN, while 190,175 do not match. We observe that the ABC score for the E-P links reported in ABC that were also matched in PENGUIN had a higher ABC score (mean ABC score for matched 0.0155 compared to all 0.0038).

2. To my 9th comment, it is still not clear how Supp Figure 4C (comparison of the number of edges v.s. the total clusters) answers the question using the choice of correct RNA-seq units (FPKM/TPM/normalized counts). How the gene expression is normalized is still an issue. The basic assumption of DESeq2 is that the gene expression dataset follows a negative binomial distribution, whereas FPKM normalized datasets do not.

We appreciate the Reviewer's insightful comments regarding the choice of RNA-seq units and their potential impact on our analysis. In reference to **Supp Figure 4C,** we concur that the method of normalization is critical for accurate interpretation. In our work, we employed the VIPER pipeline, which uses FPKM as the unit for expression. Consistent with recommended practices, we applied a stringent filtering criterion to remove genes with low expression, discarding those with FPKM values below 0.03. Of the 5,747 genes we filtered out, only 143 had some level of expression (non-zero), **making up about 2.5% of the filtered gene set**.

The Reviewer rightly points out that DESeq2 operates under the assumption that gene expression data follow a negative binomial distribution, a feature not shared by FPKM-normalized datasets (17). While FPKM and TPM units are useful for comparing transcript levels within a single sample (18), they have limitations for cross-sample comparisons and differential expression analyses (19).

We would like to emphasize that our primary focus is on relative expression within individual samples. As such, the choice between FPKM, TPM, or normalized counts would influence only approximately 2.5% of our filtered genes. In this context, we believe that our conclusions remain robust.

We hope this addresses the Reviewer's concerns and provides clarity on our methodology. Thank you for giving us the opportunity to elaborate on this aspect of our work.

1. Maston,G.A., Evans,S.K. and Green,M.R. (2006) Transcriptional regulatory elements in the human genome. *Annu. Rev. Genomics Hum. Genet.*, **7**, 29–59.

2. Vitezic,M., Bertin,N., Andersson,R., Lipovich,L., Kawaji,H., Lassmann,T., Sandelin,A., Heutink,P., Goldowitz,D., Ha,T., *et al.* (2014) CAGE-defined promoter regions of the genes implicated in Rett Syndrome. *BMC Genomics*, **15**, 1177.

3. Dixon,J.R., Selvaraj,S., Yue,F., Kim,A., Li,Y., Shen,Y., Hu,M., Liu,J.S. and Ren,B. (2012) Topological domains in mammalian genomes identified by analysis of chromatin interactions. *Nature*, **485**, 376–380.

4. van Steensel,B. and Belmont,A.S. (2017) Lamina-Associated Domains: Links with Chromosome Architecture, Heterochromatin, and Gene Repression. *Cell*, **169**, 780–791.

5. Rao,S.S.P., Huntley,M.H., Durand,N.C., Stamenova,E.K., Bochkov,I.D., Robinson,J.T., Sanborn,A.L., Machol,I., Omer,A.D., Lander,E.S., *et al.* (2014) A 3D map of the human genome at kilobase resolution reveals principles of chromatin looping. *Cell*, **159**, 1665–1680.

6. Shrinivas,K., Sabari,B.R., Coffey,E.L., Klein,I.A., Boija,A., Zamudio,A.V., Schuijers,J., Hannett,N.M., Sharp,P.A., Young,R.A., *et al.* (2019) Enhancer Features that Drive Formation of Transcriptional Condensates. *Mol. Cell*, **75**, 549–561.e7.

7. Fotuhi Siahpirani,A., Ay,F. and Roy,S. (2016) A multi-task graph-clustering approach for chromosome conformation capture data sets identifies conserved modules of chromosomal interactions. *Genome Biol.*, **17**, 114.

8. Arbelaitz,O., Gurrutxaga,I., Muguerza,J., Pérez,J.M. and Perona,I. (2013) An extensive comparative study of cluster validity indices. *Pattern Recognit.*, **46**, 243–256.

9. Watts,D.J. and Strogatz,S.H. (1998) Collective dynamics of ‘small-world’ networks. *Nature*, **393**, 440–442.

10. Tibshirani,R., Walther,G. and Hastie,T. (2002) Estimating the Number of Clusters in a Data Set Via the Gap Statistic. *J. R. Stat. Soc. Series B Stat. Methodol.*, **63**, 411–423.

11. Mohajer,M., Englmeier,K.-H. and Schmid,V.J. (2011) A comparison of Gap statistic definitions with and without logarithm function. *arXiv [stat.ME]*.

12. Davies,D.L. and Bouldin,D.W. (1979) A cluster separation measure. *IEEE Trans. Pattern Anal. Mach. Intell.*, **1**, 224–227.

13. Rousseeuw,P.J. (1987) Silhouettes: A graphical aid to the interpretation and validation of cluster analysis. *J. Comput. Appl. Math.*, **20**, 53–65.

14. Calinski,T. and Harabasz,J. (1974) A dendrite method for cluster analysis. *Commun. Stat. Theory Methods*, **3**, 1–27.

15. Suzuki,R. and Shimodaira,H. (2006) Pvclust: an R package for assessing the uncertainty in hierarchical clustering. *Bioinformatics*, **22**, 1540–1542.

16. Kimes,P.K., Liu,Y., Neil Hayes,D. and Marron,J.S. (2017) Statistical significance for hierarchical clustering. *Biometrics*, **73**, 811–821.

17. Zhao,Y., Li,M.-C., Konaté,M.M., Chen,L., Das,B., Karlovich,C., Williams,P.M., Evrard,Y.A., Doroshow,J.H. and McShane,L.M. (2021) TPM, FPKM, or Normalized Counts? A Comparative Study of Quantification Measures for the Analysis of RNA-seq Data from the NCI Patient-Derived Models Repository. *J. Transl. Med.*, **19**, 269.

18. Blog,R.-S. (2015) RPKM, FPKM and TPM, clearly explained. *RNA-Seq Blog | Transcriptome Research & Industry News*.

19. How to choose Normalization methods (TPM/RPKM/FPKM) for mRNA expression (2023) *Novogene*.

REVIEWERS' COMMENTS

Reviewer #4 (Remarks to the Author):

The authors have addressed my comments. I do not have any more comments and recommend accepting the manuscript.
